# Approaching Theoretical Performances of Electrocatalytic Hydrogen Peroxide Generation by Cobalt‐Nitrogen Moieties

**DOI:** 10.1002/anie.202301433

**Published:** 2023-04-18

**Authors:** Runjia Lin, Liqun Kang, Karolina Lisowska, Weiying He, Siyu Zhao, Shusaku Hayama, Graham J. Hutchings, Dan J. L. Brett, Furio Corà, Ivan P. Parkin, Guanjie He

**Affiliations:** ^1^ Christopher Ingold Laboratory Department of Chemistry University College London 20 Gordon Street London WC1H 0AJ UK; ^2^ Department of Inorganic Spectroscopy Max-Planck-Institute for Chemical Energy Conversion Stiftstr. 34–36 45470 Mülheim an der Ruhr Germany; ^3^ Department of Chemical Engineering University College London (UCL) London WC1E 7JE UK; ^4^ Diamond Light Source Ltd Diamond House, Harwell Campus Didcot OX11 0DE UK; ^5^ University of Göttingen Institute of Inorganic Chemistry Tamannstrasse 4 37077 Göttingen Germany; ^6^ Max Planck-Cardiff Centre on the Fundamentals of Heterogeneous Catalysis FUNCAT Cardiff Catalysis Institute School of Chemistry Cardiff University Cardiff UK; ^7^ School of Chemistry University of Lincoln Brayford Pool Lincoln LN6 7TS UK

**Keywords:** Cobalt-Nitrogen Moieties, Electrocatalyst, Hydrogen Peroxide, Oxygen Reduction Reaction

## Abstract

Electrocatalytic oxygen reduction reaction (ORR) has been intensively studied for environmentally benign applications. However, insufficient understanding of ORR 2 e^−^‐pathway mechanism at the atomic level inhibits rational design of catalysts with both high activity and selectivity, causing concerns including catalyst degradation due to Fenton reaction or poor efficiency of H_2_O_2_ electrosynthesis. Herein we show that the generally accepted ORR electrocatalyst design based on a Sabatier volcano plot argument optimises activity but is unable to account for the 2 e^−^‐pathway selectivity. Through electrochemical and *operando* spectroscopic studies on a series of CoN_*x*_/carbon nanotube hybrids, a construction‐driven approach based on an extended “dynamic active site saturation” model that aims to create the maximum number of 2 e^−^ ORR sites by directing the secondary ORR electron transfer towards the 2 e^−^ intermediate is proven to be attainable by manipulating O_2_ hydrogenation kinetics.

## Graphical abstract

A series of CoN_*x*_/carbon nanotube hybrids as oxygen reduction electrocatalysts were studied through electrochemical and operando spectroscopic methods. When cathodically biased, a reconstruction in the symmetry of Co−N_*x*_ active sites is discovered, which leads to a shift in the electrocatalytic oxygen reduction reaction selectivity from producing water to hydrogen peroxide.

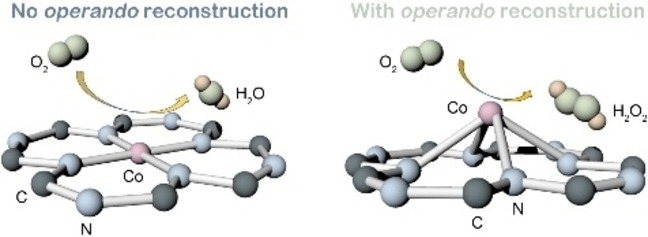

## Introduction

Electrochemical oxygen reduction reaction (ORR), in which O_2_ is reduced to either OH^−^/H_2_O by 4 e^−^‐transfer as required for fuel cell/metal‐air battery applications, or through a 2 e^−^‐pathway to produce green H_2_O_2_, is critical for global electrification and decarbonisation.[^1^, ^2^, ^3^, ^4^] Holding multiple reaction pathways can be a double‐edged sword which raises difficulty in selectivity control.^[5]^ On one hand, many 4 e^−^‐transfer‐dominated catalysts experience serious degradation when peroxide by‐product is accumulated and triggers the destructive Fenton reaction; on the other, low selectivity would lead to poor energy efficiency for H_2_O_2_ generation.[^6^, ^7^] Pt‐based catalysts exhibit excellent 4 e^−^ ORR activity and selectivity but are expensive and unstable for mass utilization.^[8]^ Affordable oxygen‐functionalized carbons show high 2 e^−^‐transfer selectivity but sluggish kinetics.^[9]^ Hence, a trade‐off among activity, selectivity and cost, restricts the current ORR catalyst design. Transition metal (TM) catalysts are promising candidates for solving the deadlock owing to their tuneable catalytic behaviour and abundance. However, TM‐based catalysts suffer from poor stability as fuel cell cathodes or low selectivity when employed for generating H_2_O_2_ due to insufficient understanding on their 2 e^−^‐pathway mechanism at the atomic level.^[10]^ Investigating the precise origin of selectivity towards the 2 e^−^‐pathway is vital not only for boosting H_2_O_2_ production efficiency, but also to enhance 4 e^−^‐catalyst durability by avoiding sites responsible for 2 e^−^ ORR at the synthesis stage.

Herein, we show that the generally accepted Sabatier volcano plot argument optimises ORR activity but is unable to account for the 2 e^−^‐pathway selectivity; an extended “dynamic active site saturation” model that examines in addition the hydrogenation kinetics linked to the OOH* adsorption energy enables us to resolve the activity‐selectivity compromise. Electrochemical and *operando* spectroscopic studies on the ORR process governed by a series of Co−N_*x*_/carbon nanotube hybrids indicate that manipulating O_2_ hydrogenation kinetics can direct the secondary ORR electron transfer step towards the 2 e^−^ intermediate, accordingly maximising the population of the 2 e^−^ ORR sites. Control experiments reveal the O_2_ hydrogenation chemistry is related to a catalyst reconstruction with lower symmetry around the Co active centre induced by applying cathodic potentials. The optimised catalyst exhibits a ≈100% H_2_O_2_ selectivity and an outstanding activity with a high potential of 0.82 V *versus* the reversible hydrogen electrode to reach the ring current density of 1 mA cm^−2^ by using rotating ring‐disk electrode (RRDE) measurement, which is the best‐performing 2 e^−^ ORR electrocatalyst reported to date, and approaches the thermodynamic limit.

## Results and Discussion

**Figure 1:**
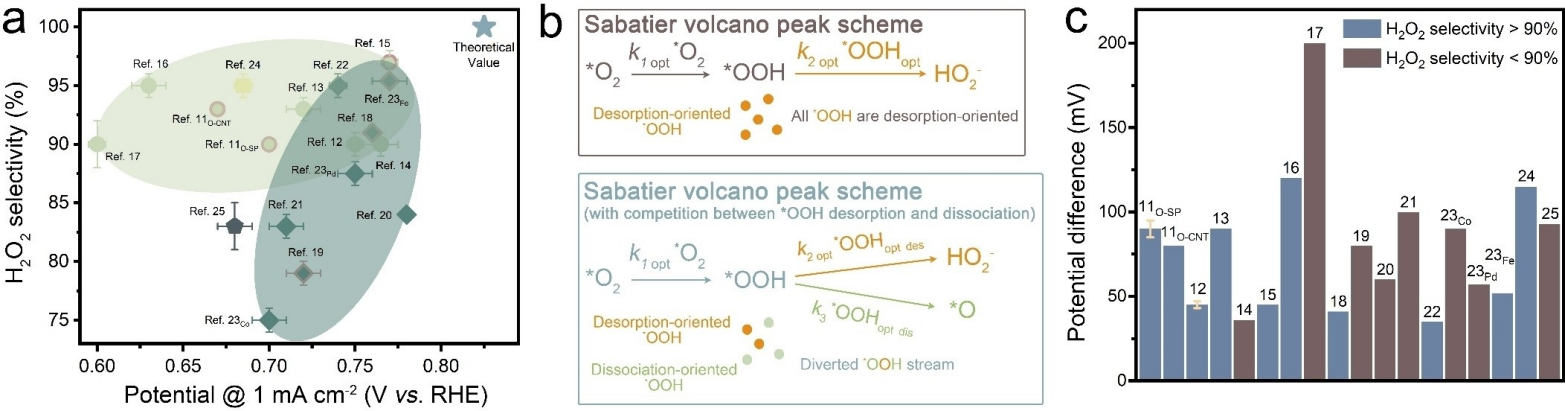
a) Comparison of $E_{{@1\;\mathrm{mA}\;\mathrm{cm}}^{-2}}$ (the potential at which ring current density reaches 1 mA cm^−2^, more discussion in Supporting Information Figure S2) and the corresponding selectivity for H_2_O_2_ electrosynthesis in recently reported state‐of‐the‐art electrocatalysts (in alkaline medium, examined by RRDE system, more information available in Supporting Information Table S1).[^11^, ^12^, ^13^, ^14^, ^15^, ^16^, ^17^, ^18^, ^19^, ^20^, ^21^, ^22^, ^23^, ^24^, ^25^] b) Thermodynamic and kinetic analysis of 2 e^−^‐ORR process based on the well‐accepted Sabatier volcano peak theory and the 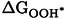 deviation model proposed in this work (assuming the reaction rate of each elementary step is determined by the reaction rate constant (*k*) and the population of specific intermediate.^[26]^ c) Comparison of ${\Delta E}_{{@1\;\mathrm{mA}\;\mathrm{cm}}^{-2}}$ (the potential difference between $E_{{@1\;\mathrm{mA}\;\mathrm{cm}}^{-2}}$ and *E*_onset_) on the electrocatalysts displayed in (a).

Electron transfer to the O_2_ molecule, whether directed to the 2 e^−^ or 4 e^−^ reduction, occurs in multiple elementary steps in which electrons are transferred one at a time. Both 2 e^−^ or 4 e^−^ reductions initiate with the formation of *OOH intermediates in the first step, and are likely differentiated by whether the second electron transfer leads to the formation of HO_2_^−^ intermediates (2 e^−^‐pathway) or dissociation of the O−O bond in the intermediate (4 e^−^‐pathway). A selection of data from cutting‐edge 2 e^−^ ORR electrocatalysts in the recent literature have been collected, on which a statistical analysis has been established to identify possible correlations between atomic features of the active site and selectivity (Figure 1a and Supporting Information Figure S1). Most of the metal‐free catalysts (i.e. refs. [11, 13, 16] in the light green region) tend to exhibit high selectivity but poor kinetic activity while the electrocatalytic behaviour is the other way round for many of the metal‐based catalysts (i.e. refs. [19, 20, 21] in the dark green region), indicating the existence of a compromise between enduing high 2 e^−^ reaction activity and selectivity. Interestingly, none of the reported catalysts reaches the thermodynamic limit of 2 e^−^ ORR performance (blue star in Figure 1a, more information in Supporting Information Figure S2) despite many of them (dots with pink outer shells in Figure 1a), according to computational studies, claim to approach the Sabatier volcano peak (refers to the optimised OOH* adsorption energy for O_2_ to H_2_O_2_ conversion predicted on the basis of thermodynamic analysis,[^11^, ^19^] more information in Supporting Information Figure S1b) by either tailoring the active centres directly[^11^, ^14^] or their coordination environments.[^15^, ^19^] The notable gap between the observed experimental results and the thermodynamic limit challenges the correctness of the “volcano plot peak worship” catalyst design approach meanwhile demands the study of 2 e^−^ ORR from a comprehensive understanding of kinetic processes. We have recently proposed an alternative “dynamic active site saturation” theory^[27]^ according to which it is believed the worse‐than‐expected 2 e^−^ ORR activity can be attributed to the competing 2 e^−^ and 4 e^−^ reaction pathways (more specifically, the competitions between OOH* desorption and dissociation reactions). A schematic diagram of the possible reaction rate evolution of each electron transfer step during the ORR is given in Figure 1b, where *k*_1 opt_ *O_2_ and *k*_2 opt_ *OOH_opt_ (brown box) represent the O_2_* hydrogenation and OOH* desorption rates of a catalyst which complies with the Sabatier principle for the 2 e^−^ reaction pathway. When taking the competition between OOH* desorption and dissociation into account (blue box, in which *k*_1 opt_ *O_2_, *k*_2 opt_ *OOH_opt des_ and *k*_3_ *OOH_opt dis_ represent the rates of O_2_* hydrogenation, OOH* desorption and dissociation, respectively), optimised H_2_O_2_ production will be attained only if the desorption‐oriented OOH* (orange dots in Figure 1b) outweighs the dissociation‐oriented OOH* (green dots in Figure 1b). Part of the OOH*, Which are required to desorb to maximise H_2_O_2_ selectivity will instead proceed to the 4 e^−^ pathway if the desorption and dissociation steps share comparable rates (which accordingly makes *k*_2 opt_ *OOH_opt des_ smaller than *k*_2 opt_ *OOH_opt_). The H_2_O_2_ generation rate will consequently be restricted by the diminished numbers of desorption‐oriented OOH* despite the catalyst obeying Sabatier principle. In this case, the 2 e^−^ ORR is neither limited by the hydrogenation of O_2_ nor desorption of OOH*, but the competition between the desorption and dissociation of OOH*. This provides credible explanation to the excellent onset (due to the optimal O_2_ hydrogenation and OOH* desorption) but unsatisfactory $E_{{@1\;\mathrm{mA}\;\mathrm{cm}}^{-2}}$ (due to the limited number of OOH* that are prone to be desorbed) of some reported 2 e^−^ ORR catalysts (i.e. refs. [17, 21, 25] in Figure 1c, more discussion available in Supporting Information Note 1). Considering ORR is mass‐transfer rather than kinetics limited, avoiding performance saturation by boosting active site population is not predicted to be as effective as it was for the kinetics‐limited electrocatalytic system in ref. [27]. In other words, saturation of ORR active site can be prevented, should the OOH* desorption/dissociation competition be optimized. It is considered that the OOH* desorption/dissociation competition can be tuned by regulation of 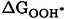 (i.e. alterations in O_2_* hydrogenation rate could potentially lead to a higher population of desorption‐oriented OOH* in Figure 1b blue box scheme, accordingly increasing the rate of OOH* to HO_2_^−^ conversion despite a possible inferior reaction rate constant due to 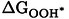 deviation, more discussion available in Supporting Information Note 1). Though the deviation in 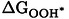 from the optimal Sabatier value would sacrifice the performance of onset potentials, an improved $E_{{@1\;\mathrm{mA}\;\mathrm{cm}}^{-2}}$ will still be attainable if the benefit from the enlarged population of desorption‐oriented OOH* outweighs the reduced kinetic activity. This opens up an exciting opportunity to synergise activity and selectivity by an improved rational design concept for the electrocatalyst.

**Figure 2:**
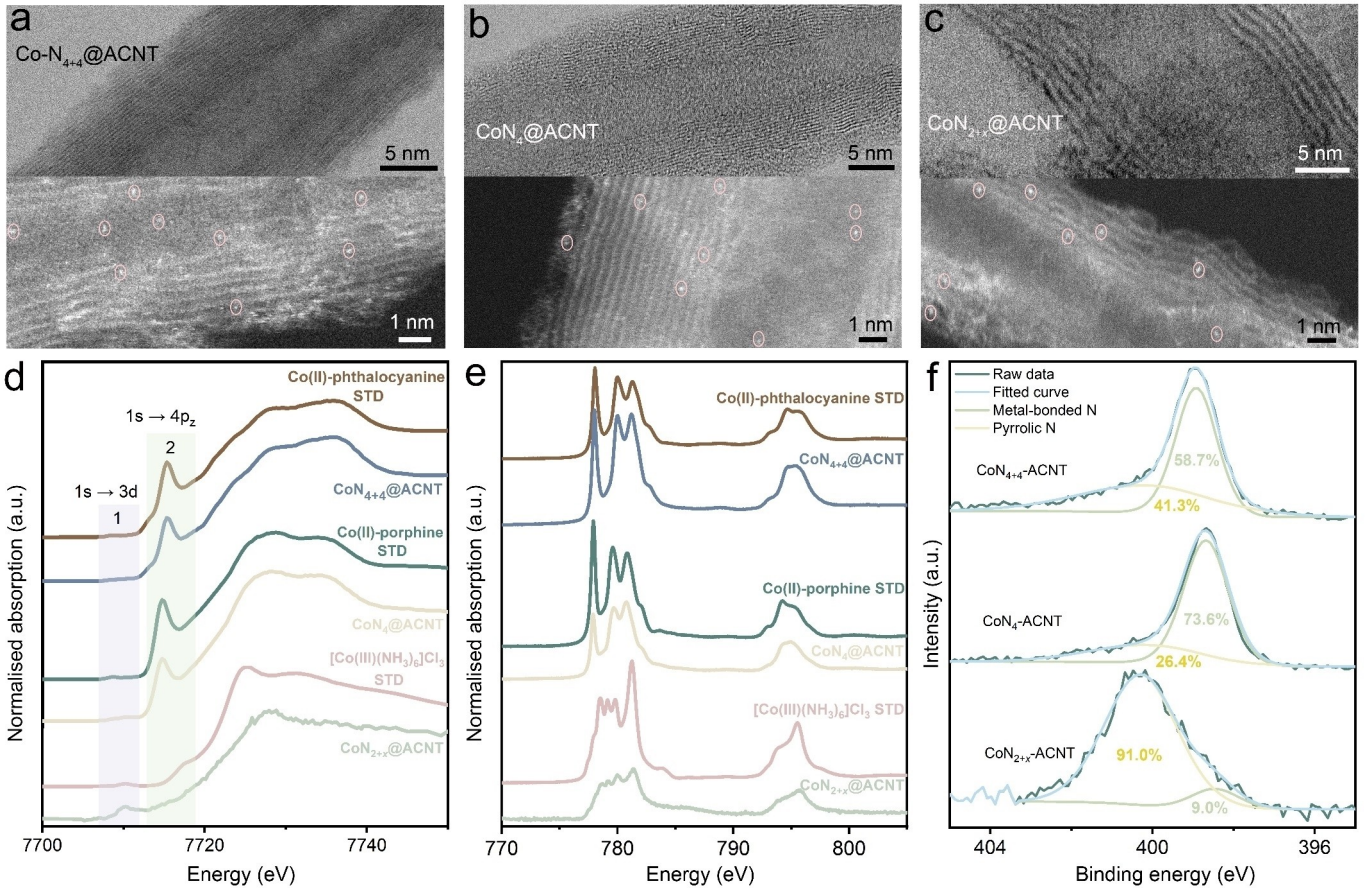
TEM (upper panel) and HAADF‐TEM (lower panel) images of a) CoN_4+4_‐ACNT, b) CoN_4_‐ACNT and c) CoN_2+*x*_‐ACNT. d) Co K‐edge HERFD‐XANES, e) Co L_3_‐edge NEXAFS and f) XPS N 1s spectra of CoN_*x*_‐ACNT and reference samples.

To explore this possibility, a series of CoN_*x*_/annealed carbon nanotube hybrids (denoted as CoN_4+4_‐ACNT, CoN_4_‐ACNT and CoN_2+*x*_‐ACNT) with different Co and N coordination environments were synthesised by heterogenization of different molecular CoN_*x*_ complexes onto CNTs via van der Waals force and hydrogen bonding (more information available in Supporting Information Methodology section). Though the use of cobalt‐based molecular catalyst in ORR can be traced back to 1964^[28]^ and they have been intensively studied afterwards in terms of onset potential and current density comparison,[^29^, ^30^] understanding of the correlation between reaction selectivity‐atomic coordination is still limited and demands comprehensive investigation. Scanning transmission electron microscopy (STEM) was used to investigate the morphological information of the CoN_*x*_‐ACNTs. The CNT framework, which provides mechanical/electron‐conductive support for metal active sites, can be identified in the upper panel of Figures 2a–c. The monodispersed bright dots (highlighted in pink circles) in the high‐angle annular dark‐field (HAADF) STEM images (Figures 2a–c lower panel and Supporting Information Figure S3) indicate the presence of isolated cobalt species in CoN_*x*_‐ACNTs.

To understand physical and chemical properties of as‐prepared electrocatalysts, ex situ X‐ray absorption spectroscopy (XAS) and X‐ray photoelectron spectroscopy (XPS) measurements were performed. The high energy resolution fluorescence detected X‐ray absorption near edge structure (HERFD‐XANES) spectra (Figure 2d, the corresponding first derivative plot is shown in Supporting Information Figure S4) are characterised by two regions: the weak pre‐edge peaks at 7708–7712 eV (labelled as transition region 1) and the strong edge peaks at 7714–7716 eV (labelled as transition region 2). The Co K‐edge spectra for CoN_4+4_‐ACNT and CoN_4_‐ACNT are nearly identical to their Co^II^‐phthalocyanine and Co^II^‐porphine precursors respectively (indicating the molecular structure of the precursors were well‐preserved during the heterogenization process), except the slightly weaker second transitions (7714.7 eV for CoN_4+4_‐ACNT and 7715.3 eV for CoN_4_‐ACNT) due to ACNT encapsulation.^[31]^ The peaks in transition region 1 can be attributed to dipole forbidden 1s to 3d quadrupole transition.^[32]^ The peaks in transition region 2 which have usually been observed in Co−N_4_ structures with square planar environment,[^32^, ^33^] can be assigned to a shake down feature that involves 1s to 4p_*z*_ transition mixed with a 3d character due to a simultaneous ligand to metal charge transfer. As for the CoN_2+*x*_‐ACNT sample, its absorption edge position witnesses a ≈1 eV shift to a higher energy state compared to CoN_4+4_‐ACNT and CoN_4_‐ACNT. Through referencing to XANES of the low spin Co^3+^ in [Co(NH_3_)_6_]Cl_3_, the CoN_2+*x*_‐ACNT catalyst is dominated by low spin octahedral Co^3+^ centres. Similar conclusion can also be reached from Co K_β_ X‐ray emission spectroscopy (XES) measurement as Supporting Information Figure S5 demonstrates. The surface atomic structure was investigated through near edge X‐ray absorption fine structure (NEXAFS) measurements. As illustrated in Figure 2e, no significant shift of the Co L_3_‐edge NEXAFS characteristic peaks of CoN_4+4_‐ACNT, CoN_4_‐ACNT and CoN_2+*x*_‐ACNT can be observed when compared to their corresponding reference samples. The XPS analysis (Supporting Information Figure S6a) agrees with the NEXAFS measurement that the cobalt centres in CoN_2+*x*_‐ACNT show higher oxidation states compared to the Co atoms in CoN_4+4_‐ACNT/CoN_4_‐ACNT. The XPS N 1s spectra (Figure 2f) of all CoN_*x*_‐ACNT samples display two main peaks corresponding to pyrrolic N (≈400.5 eV) and metal‐bonded N (≈399 eV).^[34]^ The CoN_4+4_‐ACNT contains a higher proportion of surface N atoms which are not directly bonded to the Co centres (41.3 at%) compared to that of CoN_4_‐CNT (26.4 at%), consistent with the molecular structure. Extended X‐ray absorption fine structure (EXAFS) studies confirm the coordination environment of CoN_4+4_‐ACNT is in good agreement with that of Co phthalocyanine, which is featured with 4 direct coordinated N atoms and additional N atoms in the second coordination shell (Supporting Information Figures S7). Although CoN_2+*x*_‐ACNT is estimated (from the NEXAFS measurement) to exhibit the highest coordination number of the central metal among the three materials, XPS N 1s spectrum indicates most of its N species (91 at %) are not directly bonded with the central Co. Computational simulation studies in literatures suggest the Co centres in metal‐pyrrole structure are possibly bonded with two pyrrolic N and surrounded with N atoms in polypyrrole chains (whose formation is likely due to the polymerisation of the pyrrole precursor).^[35]^ The stronger Co K‐edge pre‐edge peak in HERFD‐XANES of CoN_2+*x*_‐ACNT compared to that of [Co(NH_3_)_6_]Cl_3_ could be a fingerprint of the Co being in a lower symmetry (i.e. non‐centrosymmetric) environment when coordinated to polypyrrole chains relative to the square planar porphyrin coordination.^[36]^ XPS O 1s (Supporting Information Figure S6b) and O K‐edge NEXAFS (Supporting Information Figure S10) analysis suggest all three catalysts, despite showing minor differences in spectroscopic responses, share similar O species which mainly come from the ACNT substrates.

The physical/chemical results shown above illustrate two important features of all as‐prepared CoN_*x*_‐ACNT catalysts. First, Co species are in single‐atom state. Second, in CoN_4_‐CNT, the central Co^2+^ is bridged by four N atoms; in CoN_4+4_‐CNT, Co^2+^ centre is surrounded by not only first‐shell, but also second‐shell N atoms; as for CoN_2+*x*_‐CNT, its Co is in 3+ oxidation state and directly bonded with two pyrrolic N. The Co−N_2_ moieties are surrounded and influenced by a protein‐like tertiary structure made of polypyrrole chains.

**Figure 3:**
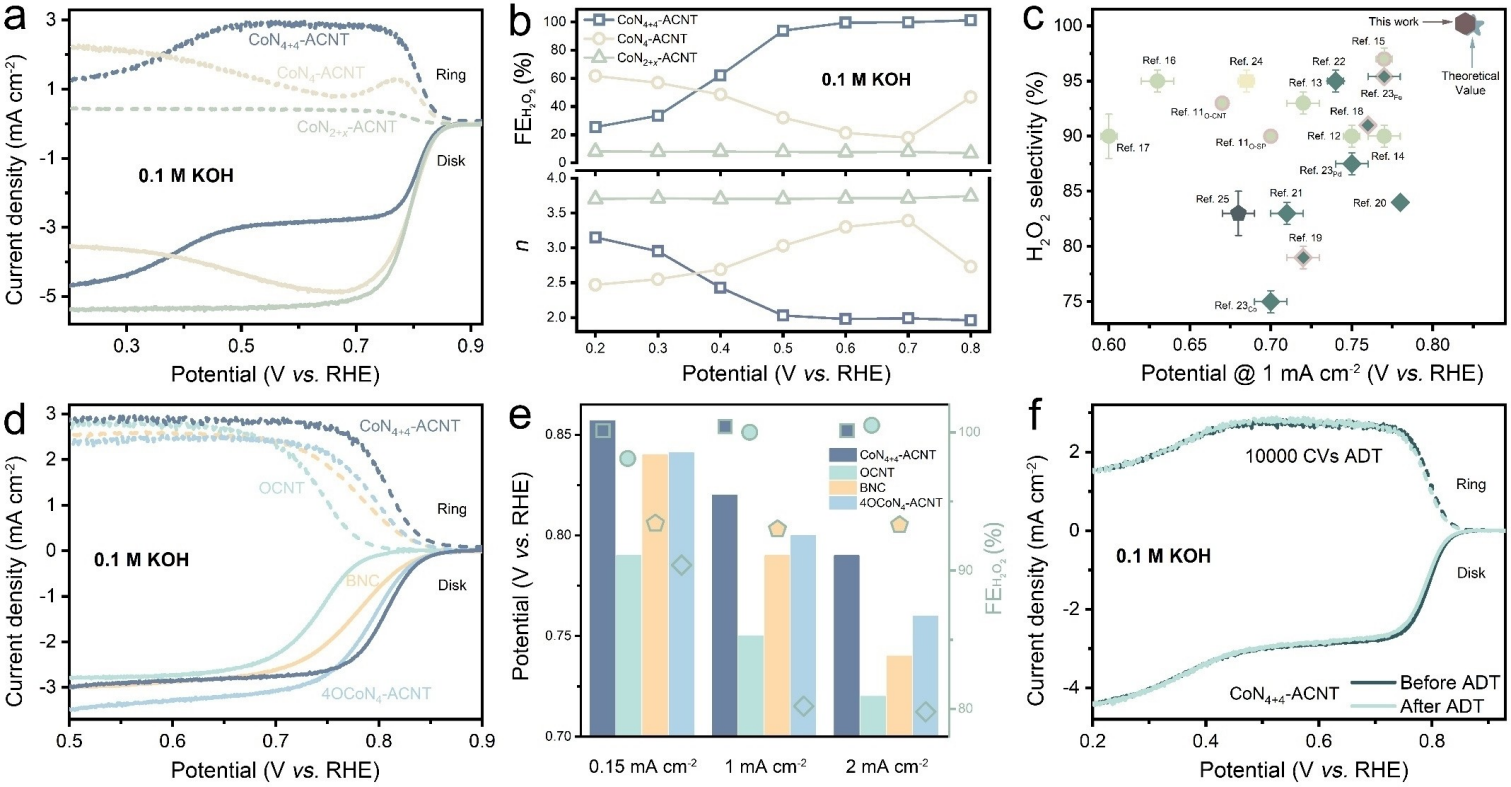
a) Comparison of ORR performance of the CoN_*x*_‐ACNT catalysts at 1600 rpm and their simultaneous H_2_O_2_ detection current densities at the ring electrode in O_2_‐saturated 0.1 M KOH; b) calculated Faraday efficiency towards H_2_O_2_ (${\mathrm{FE}}_{H_{2}O_{2}}$ ) and average number of electrons transferred to each O_2_ molecule (*n*) as a function of the applied potential. c) Comparison of the $E_{{@1\;\mathrm{mA}\;\mathrm{cm}}^{-2}}$ and selectivity for H_2_O_2_ electrosynthesis on CoN_4+4_‐ACNT and previously reported electrocatalysts in alkaline medium examined by RRDE system. Comparison of d) ORR polarization curves and e) activity (columns) and selectivity (dots) for CoN_4+4_‐ACNT and other prepared catalysts that represent the current 2 e^−^ catalyst design mainstream. f) ADT measurement of CoN_4+4_‐ACNT. More electrochemical data is available in Supporting Information Table S4–S16.

Measured in 0.1 M KOH aqueous solution using a rotating ring‐disk electrode system (RRDE, more experimental set‐up details available in in Supporting Information Methodology section), as can be reflected from Figure 3a and b, the CoN_*x*_‐ACNT materials with different Co−N coordination environments show similar ORR onset (≈0.86 V vs. RHE) but greatly varied selectivity, with a ≈100% H_2_O_2_ production Faraday efficiency (denoted as ${\mathrm{FE}}_{H_{2}O_{2}}$ ) and a ≈2 electron transfer number (denoted as *n*) for CoN_4+4_‐ACNT in a wide‐potential region ranging from ≈0.50–0.85 V vs. RHE; mixed selectivity for CoN_4_‐ACNT and high 4 e^−^‐pathway selectivity (${\mathrm{FE}}_{H_{2}O_{2}}$ <10%) for CoN_2+*x*_‐ACNT. A similar selectivity trend can be observed when the tests are performed in a neutral medium (Supporting Information Figure S11). The performance of CoN_4+4_‐ACNT (*E*_onset_=0.857 V vs. RHE, $E_{{@1\;\mathrm{mA}\;\mathrm{cm}}^{-2}}$ =0.82 V vs. RHE in 0.1 M KOH) surpasses all previously reported electrocatalysts for H_2_O_2_ electrosynthesis at both low and high H_2_O_2_ generation rate conditions and nearly reaches the thermodynamic limit (Figure 3c and Supporting Information Figure S12), indicating the successful solution of the “activity‐selectivity dilemma” that puzzles the current 2 e^−^ ORR electrocatalysts design. Bulk H_2_O_2_ electrosynthesis test was also performed with a H‐type cell configuration whose results agree with the RRDE measurement (Supporting Information Figure S13), indicating CoN_4+4_‐ACNT can be a promising candidate as a cathode catalyst for H_2_O_2_ production.

To highlight the superiority of CoN_4+4_‐ACNT over other ORR electrocatalysts, oxygen‐functionalized CNT (denoted as OCNT), boron and nitrogen co‐doped carbon (denoted as BNC), and oxidized CoN_4_‐ACNT (denoted as *x*OCoN_4_‐ACNT) that represent three popular 2 e^−^ ORR electrocatalyst design strategies were synthesised, examined and compared (Figures 3d, e and Supporting Information Figure S14).[^11^, ^19^, ^25^] Consistent with previous reports, carbons that are functionalized by oxygen groups exhibit enhanced 2 e^−^ ORR behaviour.[^11^, ^12^, ^37^] The ≈100% ${\mathrm{FE}}_{H_{2}O_{2}}$ of OCNT (Supporting Information Figure S14a) confirms the effectiveness of the “O‐functionalization” strategy. However, OCNT shows relatively poor activity, requiring 67, 70 and 72 mV higher overpotentials for reaching ring current densities of 0.15, 1 and 2 mA cm^−2^, respectively compared with that of CoN_4+4_‐ACNT (Figure 3e and Supporting Information Figure S14c). Many attempts were made to manufacture more active 2 e^−^ ORR catalysts by heteroatom doping including nitrogen,[^24^, ^38^] boron,[^25^, ^39^] and sulfur.^[22]^ Nevertheless, as confirmed in Figure 3e and Supporting Information Figures S14a and c, BNC demonstrates inferior reaction kinetics compared to CoN_4+4_‐ACNT, particularly at high H_2_O_2_ generation rate (it requires 30 and 50 mV higher overpotentials to reach ring current densities of 1 and 2 mA cm^−2^ and >10% ${\mathrm{FE}}_{H_{2}O_{2}}$ decrease compared to that of CoN_4+4_‐ACNT) despite its excellent onset potential (only 17 mV higher overpotential than that of CoN_4+4_‐ACNT). To address the poor activity of metal‐free 2 e^−^ electrocatalysts, many efforts have been devoted to convert traditional TM‐based 4 e^−^ ORR electrocatalysts to 2 e^−^ structures by tailoring the active centres and/or their surrounding environment.[^7^, ^10^, ^21^, ^29^, ^40^] The most well‐known approach is the oxidation treatment.^[41]^ Therefore, CoN_4_ moieties (whose composites with ACNT show mixed ORR selectivity as indicated in Figure 3b) were treated with HNO_3_ and then its (refers to the HNO_3_‐treated CoN_4_) hybrid with ACNT was employed as an ORR electrocatalyst (Supporting Information Figure S15). The performance of *x*OCoN_4_‐ACNT materials demonstrate a strong dependence on the acid treatment duration (selectivity increases but activity decreases with the extension of acid treatment period) as can be seen in Supporting Information Figures S15a and b. In agreement with the conclusion reached in Figure 1a,[^10^, ^11^, ^41^] there is a competition between activity and selectivity; the modified materials demonstrate better kinetic activities than metal‐free ones but unsatisfactory selectivity (${\mathrm{FE}}_{H_{2}O_{2}}$ <90%). As a result, none of the HNO_3_‐treated catalysts, even the best performing one (4 h of HNO_3_ treatment, denoted as 4OCoN_4_‐ACNT), displays competitive H_2_O_2_ production activity compared to CoN_4+4_‐ACNT. The decreasing ring and disk current densities obtained at the same overpotential by prolonged HNO_3_ treatment (Supporting Information Figures S15d–f) demonstrate that the improvement in ${\mathrm{FE}}_{H_{2}O_{2}}$ is achieved mainly by blocking the 4 e^−^ ORR pathway reaction chain rather than by boosting the 2 e^−^ ORR pathway kinetics. This result highlights the intrinsic activity/selectivity compromise associated with the strategy of electrocatalyst optimisation through a volcano plot argument based on a destruction‐driven approach (i.e. by eliminating non‐2 e^−^ ORR sites).[^19^, ^20^, ^21^, ^41^] Our dynamic active site saturation model, instead, is a construction‐driven approach that aims to create the maximum number of 2 e^−^ ORR sites by directing the secondary ORR electron transfer step towards the 2 e^−^ intermediate. For further clarification, equivalent current density analysis (named as ECD) is performed. Results presented in Supporting Information Figure S16c identify reduced overall ORR activities of CoN_4_‐ACNT after oxidation treatments. That is to say, the ORR process governed by the *x*OCoN_4_‐ACNT series (especially 24OCoN_4_‐ACNT) is restrained by either insufficient numbers of active sites (as implied by the metal loading quantification results displayed in Supporting Information Table S3, uncontrollable harsh oxidation treatment could eradicate part of the pristine metal active sites) or at least one sluggish rate‐determining step within its 2/4 e^−^ reaction chains (due to low activities of the modified active species). The ECD values of CoN_*x*_‐ACNT series catalysts are also evaluated (Supporting Information Figure S16b). Their almost identical ECDs suggest the ECD of a catalyst should be close to the 4 e^−^ ORR mass transfer limitation if it shows full kinetic activity regardless of its selectivity. Hence, research should focus on not only selectivity and onset potential, but also ECD to represent the actual activity when evaluating 2 e^−^ ORR electrocatalysts.

The catalyst durability has been investigated through accelerated degradation test (ADT) analysis, demonstrated in Figure 3f. The ORR linear sweep voltammetry (LSV) of CoN_4+4_‐ACNT remains almost identical after 10 000 cycles of ADT operation. No ${\mathrm{FE}}_{H_{2}O_{2}}$ change can be witnessed (Supporting Information Figure S17) which confirms the superb stability of CoN_4+4_‐ACNT for H_2_O_2_ electrosynthesis. The desirable durability might be attributed to the rigid and very stable Co−N moieties that prevent leaching of cobalt ions (which can behave as an ignitor for Fenton reactions) into the electrolyte.^[19]^ Previous literature suggests disruptive radicals (i.e. OH⋅) can also be generated via photo/electrocatalytic reduction of H_2_O_2_.^[42]^ No reduction current can be observed for CoN_4+4_‐ACNT (in high‐potential region) during H_2_O_2_ reduction reaction (H_2_O_2_RR) tests (Supporting Information Figure S18), inferring the supressed H_2_O_2_RR activity of CoN_4+4_‐ACNT and the suppression in the production of destructive radicals.

**Figure 4:**
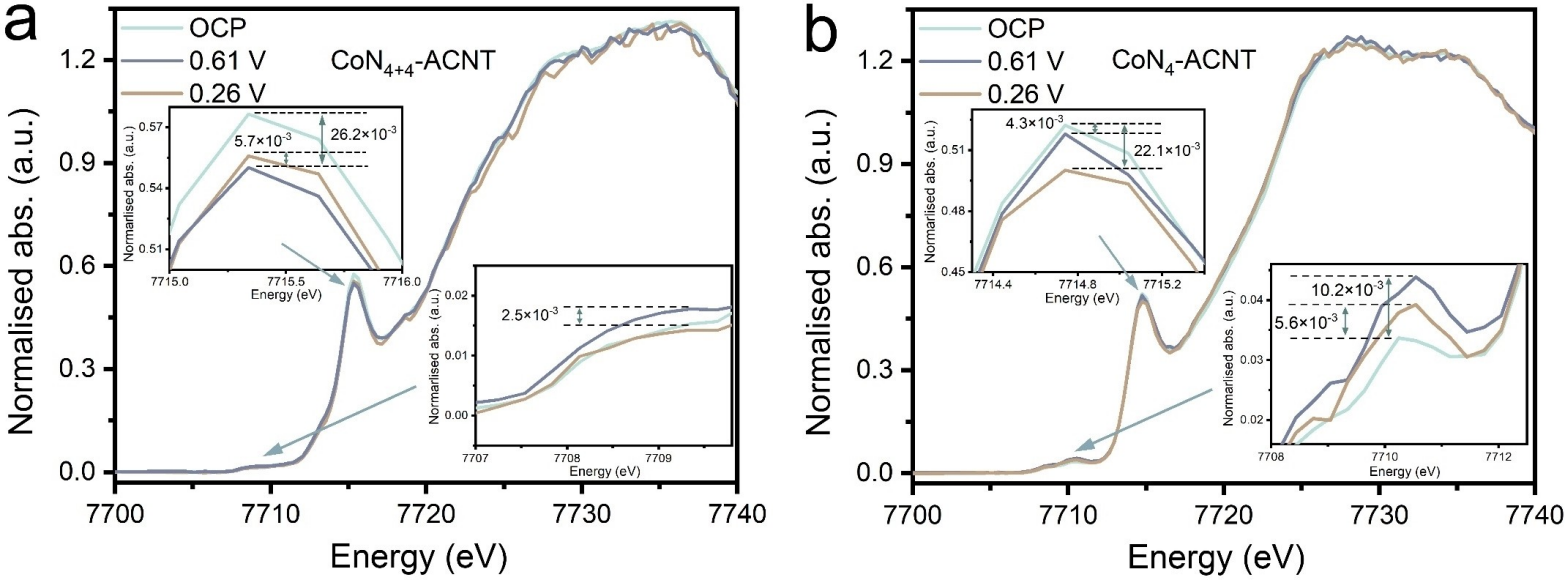
*Operando* HERFD‐XANES Co K‐edge spectra of a) CoN_4+4_‐ACNT and b) CoN_4_‐ACNT acquired under chronoamperometry test at OCP, 0.61 and 0.26 V vs. RHE.

To elucidate the varied selectivity of the CoN_*x*_‐ACNT catalysts, control experiments were conducted and indicated the selectivity variation is irrelevant to neither the interaction between the CoN_*x*_ complexes and the oxygen‐containing carbon substrate nor the metal loading (Supporting Information Figures S19–S22). Attention is then directed to the cobalt‐nitrogen coordination where the three catalysts display the biggest difference. To correlate atomic‐level coordination with ORR selectivity, *operando* HERFD‐XANES Co K‐edge measurement was performed (further information on the experiment setup is available in Supporting Information Figure S23). Acquiring XANES in HERFD mode leads to an enhanced spectral resolution, thus expanding the *operando* study of electrocatalysts beyond valence state observation to coordination environment analysis.^[43]^ However, the price for the higher energy resolution to resolve the subtle changes is the difficulties in the measurement because the signal intensity of HERFD‐XANES is generally more than one order of magnitude weaker than that of the conventional total fluorescence yield (TFY)/partial fluorescence yield (PFY)‐XANES.^[44]^ Hence, HERFD‐XANES normally shows lower signal‐to‐noise ratio (as can be noticed in this work and previous literatures)^[44]^ but the technique is considered reliable (please see Supporting Information Note 2 for more clarifications). The *operando* measurement was first established on CoN_4+4_‐ACNT and CoN_4_‐ACNT owing to their similar Co coordination chemistry but varied selectivity. The study on CoN_2+*x*_‐ACNT (Supporting Information Figure S24) will be discussed in a later section. As the applied voltage moves from open circuit voltage (OCP) to 0.61 and 0.26 V vs. RHE, no obvious edge position shift can be noticed in the 1s to 3d transition region (7708–7712 eV, containing information about structural symmetry and oxidation state^[32]^) nor in the 1s to 4p_*z*_ transition region (7714–7716 eV, reflecting the charge transfer from ligand to metal^[45]^). This indicates the oxidation state of Co in both samples remains stable (and indirectly excludes the adsorption of charged ions on Co). A small intensity alteration for both peaks can be observed as a function of applied voltage (Figures 4a and b); even if small (this may reflect the fact that only a small fraction of the Co sites are modified), the intensity change is reproducible in repeated experiments. Variations in the 1s to 3d transition intensity can be assigned to a change of symmetry around the Co ions upon application of a cathodic bias, such as a movement of Co off the molecular plane to a non‐centrosymmetric position or adsorption of reaction intermediates on the Co centre, resulting in formation of a penta‐coordinated structure which subsequently increases the 1s to 3d transition intensity.[^31^, ^36^] The Co displacement can also cause p and d type orbital hybridisation, accordingly leading to a decrease in 1s to 4p_*z*_ transition intensity.^[31]^ In other words, the catalysts witnessed reconstruction in their coordination environment (more in‐depth computational analysis on the Co displacement phenomenon is available in Supporting Information Figure S25). Different intensity alterations are observed for 1s to 3d or 4p_*z*_ transitions on CoN_4+4_‐ACNT and CoN_4_‐ACNT: referenced to the OCP condition, CoN_4_‐ACNT shows a larger 1s to 4p_*z*_ transition intensity change at 0.61 V than at 0.26 V vs. RHE, while the changes in 1s to 3d transition intensity are the other way around. This observation may imply the geometry evolution (from planar to penta‐coordinated) is induced by multiple factors, for instance Co displacement and adsorption of ORR intermediates on the Co centre. Considering the hydrogenation of *O_2_ is normally regarded as the most thermodynamically unfavourable ORR step, the accumulated intermediate could be *O_2_[^21^, ^22^] (more discussions in Supporting Information Note 3).

In the case of CoN_4_‐ACNT, upon decreasing cathodic bias (from 0.61 to 0.26 V vs. RHE) the sample shows a weakening 1s to 3d transition (lower‐right inset in Figure 4b). We interpret this change as smaller accumulation of *O_2_ and hence a more favourable *O_2_ hydrogenation kinetics at the lower bias. Such observation points to a positive correlation between hydrogenation of *O_2_ and 2 e^−^‐ORR selectivity, that is, a more favourable *O_2_ hydrogenation kinetics could lead to better OOH* desorption selectivity. This gives reasons to the increased ORR 2 e^−^ selectivity of CoN_4_‐ACNT from 0.7 to 0.2 V vs. RHE (Figure 3a). As for CoN_4+4_‐ACNT, when the potential is set at 0.61 V vs. RHE, the 1s to 3d transition intensity growth (referenced to the OCP condition) of CoN_4+4_‐ACNT is much less than that of CoN_4_‐ACNT (lower‐right insets in Figures 4a and b). This indicates less accumulation of *O_2_, accordingly pointing to more favourable *O_2_ hydrogenation kinetics on the prior catalysts. As a result, CoN_4+4_‐ACNT shows higher 2 e^−^‐ORR selectivity than CoN_4_‐ACNT. The above analysis validates the hypothesis that the OOH* desorption/dissociation competition can be tuned by adjusting the *O_2_ hydrogenation kinetics as proposed in Figure 1b. It is noteworthy that the relationship between *O_2_ hydrogenation and OOH* desorption could vary by active site. In the following work, more comprehensive computational studies (especially from reaction dynamic perspective) are required to provide a better understanding on the positive *O_2_ hydrogenation‐OOH* desorption correlation of the molecular catalysts.

The similar 2 e^−^‐ORR onset‐potential of CoN_4+4_‐ACNT and CoN_4_‐ACNT (Figure 3a) implies the identical *O_2_ hydrogenation kinetics of the two catalysts at small overpotentials. To elucidate the reasons behind the varied *O_2_ hydrogenation kinetics of CoN_4+4_‐ACNT and CoN_4+4_‐ACNT at larger overpotentials, attention is then directed to the 1s to 4p_z_ transition. When biased at 0.61 V vs. RHE, CoN_4+4_‐ACNT witnesses a more obvious 1s to 4p_z_ feature intensity fall (referenced to the OCP condition) compared to that of CoN_4_‐ACNT (upper‐left insets in Figures 4a and b), which implies more active “Co centre wandering” phenomena in CoN_4+4_‐ACNT. This can be ascribed to the second‐shell N in CoN_4+4_‐ACNT that facilitates the ligand to metal charge transfer between the first‐shell N and the Co centre (more discussion in Supporting Information Note 4). Interestingly, as can be inferred from Figures 4a and b, the larger the 1s to 4p_*z*_ transition intensity decrease, the smaller the 1s to 3d transition intensity increase (except the CoN_4+4_‐ACNT at 0.26 V which will be described in the following section). This can be regarded as a direct evidence of the positive relationship between the “Co centre wandering” activity and O_2_ hydrogenation kinetics. In other words, 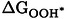 can be controlled by manipulating the ligand to metal charge transfer chemistry. In summary, CoN_4+4_‐ACNT and CoN_4_‐ACNT will witness a “Co wandering” coordination reconstruction if cathodically biased (evidencing by the decrease in 1s to 4p_z_ transition). Such reconstruction induces orbital hybridisations of the Co−N_*x*_ complexes, owing to which the *O_2_ hydrogenation kinetics of the catalysts is altered (evidencing by the variation in 1s to 3d transition intensity), eventually resulting in the shift of the OOH* desorption/dissociation competition selectivity. With above discussions, the electrochemical analysis displayed in Figure 3 and the spectroscopic observation shown in Figure 4 are consistent with the hypothesis proposed in Figure 1.

**Figure 5:**
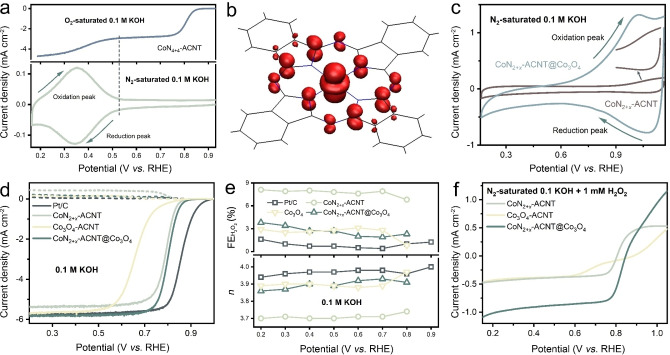
a) ORR polarisation curve (upper panel, measured in O_2_‐saturated 0.1 M KOH at 1600 rpm) and pseudocapacitive behaviour (lower panel, measured in N_2_‐saturated 0.1 M KOH at a scan rate of 20 mV s^−1^) of CoN_4+4_‐ACNT. b) Spin density plot (highlighted in red) of reduced CoN_4+4_‐ACNT from DFT calculations; one unpaired electron is located in the $3d_{z^{2}}$ orbital of Co^2+^, and one delocalised over the porphyrin ring. c) Pseudocapacitive behaviour of CoN_2+*x*_‐ACNT before and after Co_3_O_4_ hybridisation (scan rate: 20 mV s^−1^). d), e) Comparison of ORR performance of CoN_2+*x*_‐ACNT, Co_3_O_4_‐ACNT, CoN_2+*x*_‐ACNT@Co_3_O_4_ and Pt/C at 1600 rpm and the simultaneous H_2_O_2_ detection current densities at the ring electrode in O_2_‐saturated 0.1 M KOH and (b) the calculated ${\mathrm{FE}}_{H_{2}O_{2}}$ and *n* as a function of the applied potential. f) H_2_O_2_RR polarisation curves of CoN_2+*x*_‐ACNT, Co_3_O_4_‐ACNT and CoN_2+*x*_‐ACNT@Co_3_O_4_ measured in N_2_‐saturated 0.1 M KOH containing 1 mM H_2_O_2_ at 1600 rpm. More electrochemical data is available in Supporting Information Table S18–S22.

Not only can our analysis be applied to optimise the combined activity and selectivity of the 2 e^−^ ORR, but also to better understand open issues in the 4 e^−^ ORR operation. Second shell N in Co supported on N‐doped carbons (Co−N_*x*_−C) as fuel cell cathodes has been identified as a promoter of H_2_O_2_ formation that lowers the fuel cell efficiency,^[46]^ but cannot be avoided during practical synthesis especially for cathodes fabricated by high‐temperature pyrolysis. In our study, second‐shell N is present in the CoN_4+4_ moieties, and we observe a significant suppression in H_2_O_2_ production (decrease in ${\mathrm{FE}}_{H_{2}O_{2}}$ ) at low‐potential (Figures 3 and 5a upper panel). A similar H_2_O_2_ formation suppression would be highly beneficial in fuel cells and its mechanistic origin warrants further investigation. A control experiment under N_2_ atmosphere shows additional redox features in the cyclic voltammetry (CV) plot, centred at ≈0.35V vs. RHE (Figure 5a lower panel). The occurrence of this peak coincides with the onset of 4 e^−^ ORR (Figure 5a upper panel and Figure 3b). This indicates that the ORR selectivity variation in CoN_4+4_‐ACNT can occur when the catalyst is able to further accept additional electrons. Despite the noticeable redox pair, no obvious edge position shift occurs in the Co K‐edge HERFD‐XANES spectra (Figure 4a), which indicates Co centres are not the major electron acceptors. Computational studies using density functional theory (DFT) reveal that the additional electron in the reduced CoN_4+4_ moieties is localised on the surrounding N atoms instead of the metal centres (Figure 5b and Supporting Information Note 4 for more discussion). By comparison, the CoN_4_ molecule without second shell N is more difficult to reduce than CoN_4+4_ (by 1.1 eV in vacuum) and localises the additional electron on the Co centre. Compared to the electrons regulated by the Co centre, electrons on second‐shell N may result in the catalytic behaviour change of the reduced CoN_4+4_‐ACNT that promotes the O_2_ hydrogenation. This result also infers that the weak Co‐K‐edge HERFD‐XANES pre‐edge at 0.26 V vs. RHE in Figure 4a can be attributed to the reduction of the porphyrin ring (which can be seen as another factor besides the “Co wandering” phenomena that influences the O_2_* hydrogenation kinetics). Similar to CoN_4+4_‐ACNT, CoN_6+*x*_‐ACNT shows an obvious redox behaviour (Figure 5c) in a region of potential above 0.9 V vs. RHE, but limited Co edge position shift (only 0.3 eV to lower energy at 0.26 V when referenced to OCP) during the *operando* measurement (Supporting Information Figure S24), suggesting a similar redox mechanism induced by the three‐dimensional polypyrrole chains which surround the Co centres and behave as “pseudo‐second shell N” electron acceptors.

Inspired by the above results, Co_3_O_4_ was introduced into the CoN_2+*x*_‐ACNT system (denoted as CoN_2+*x*_‐ACNT@Co_3_O_4_, more characterisation and discussion available in Supporting Information Figures S27 and S30) as a redox behaviour booster (Figure 5c) for more efficient and flexible in situ electron delocalisation. Measured in O_2_‐saturated 0.1 M KOH (Figures 5d, e and Supporting Information Figure S31), the CoN_2+*x*_‐ACNT@Co_3_O_4_ shows ultra‐low ${\mathrm{FE}}_{H_{2}O_{2}}$ (≈3%) which is only one third of CoN_2+*x*_‐ACNT (≈9%) and comparable to commercial Pt/C catalyst (≈2%). A similar selectivity enhancement towards the 4 e^−^ ORR can also be observed on CoN_4+4_‐ACNT and CoN_4_‐ACNT after Co_3_O_4_ hybridisation (Supporting Information Figures S28–S31). Although enhancing 4 e^−^ ORR performance by hybridising Co−N_*x*_−C with cobalt oxides has been reported previously,[^47^, ^48^] the exact origin of the synergy mechanism is still unclear. The selectivity shift was first attributed to the average contribution from the two active centres (i.e. CoN_*x*_ and Co_3_O_4_). That is to say Co_3_O_4_ should be able to show comparable ORR kinetic activity but more 4 e^−^ ORR dominated selectivity than CoN_*x*_ moieties, changing the overall reaction selectivity. Nevertheless, as Supporting Information Figures S31a, d and g display, the selectivity shift of CoN_*x*_‐ACNT@Co_3_O_4_ materials can be witnessed prior to the ORR onset/half‐wave potential of the Co_3_O_4_‐ACNT catalyst. The almost identical ECD before and after Co_3_O_4_ hybridisation (Supporting Information Figures S31c, f and i) also disproves a contribution by Co_3_O_4_ alone. The diminished H_2_O_2_ generation after hybridisation could be due to the in situ reduction of the CoN_*x*_‐generated H_2_O_2_ by Co_3_O_4_. However, the assumption was ruled out by the inadequate H_2_O_2_RR activity of Co_3_O_4_‐ACNT (Figure 5f). Interestingly, the impact of Co_3_O_4_ hybridisation on the catalytic behaviour of the CoN_*x*_‐ACNT samples is only reflected by ORR selectivity, leaving the activity almost unchanged as implied by the ECD analysis (Supporting Information Figures S30c, f and i). In other words, the catalysts with and without Co_3_O_4_ share the same rate‐limiting step (i.e. hydrogenation of O_2_) and reaction kinetics, but the relative contribution of the desorption and dissociation of OOH* varies. This points to the possibility that Co_3_O_4_ does not directly involve in the initial reduction of O_2_ (otherwise an alteration in ORR reaction kinetics should be observed), but can regulate the catalytic process. Combining the discoveries on CoN_4+4_ and the high‐potential redox feature of CoN_*x*_‐ACNT@Co_3_O_4_ (redox reaction occurs at a potential higher than the ORR onset potential as can be inferred in Figure 5c), herein, the remaining possibility is proposed that the redox‐active Co_3_O_4_ and the N‐rich electron acceptor structures (i.e. tertiary polypyrrole chains, pyrrole N π system) firstly induce an electron delocalisation phenomenon; the delocalised charge then participates in the ORR process on the nearby CoN_*x*_ active sites and shifts the reaction selectivity (i.e. by making 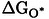 <${\Delta G}_{H{O_{2}}^{-}}$ to facilitate the dissociation of OOH*). Such conclusion was supported by a more moderate ${\mathrm{FE}}_{H_{2}O_{2}}$ drop of CoN_4+4_‐ACNT@Co_3_O_4_ compared to that of CoN_4+4_‐ACNT in its redox active potential window (Supporting Information Figure S31e). In other words, the Co_3_O_4_ advances (evidencing by the decreased H_2_O_2_ selectivity between 0.5–0.8 V vs. RHE) but does not enhance (evidencing by a similar H_2_O_2_ selectivity at 0.2 V vs. RHE) the redox‐induced electron delocalisation mechanism observed in the CoN_4+4_‐ACNT ORR system. A weakened pseudocapacitive behaviour along with a rebound in ${\mathrm{FE}}_{H_{2}O_{2}}$ can be observed for CoN_2+*x*_‐ACNT@Co_3_O_4_ after ADT measurement (Supporting Information Figure S32) which further validates the above explanation.

## Conclusion

In summary, this work firstly identified the limitations of the popular Sabatier principle‐driven 2 e^−^ ORR electrocatalyst design, owing to which current ORR electrocatalysts are restricted by activity‐selectivity compromise. A “dynamic active site saturation” model was then predicted to synergise the activity and selectivity of the 2 e^−^ ORR electrocatalysts. A series of CoN_*x*_‐ACNT materials were designed and manufactured to correlate ORR selectivity with Co−N atomic coordination and evaluate the possibility of boosting both activity and selectivity simultaneously. The CoN_4+4_‐ACNT material exhibits a ≈100% ${\mathrm{FE}}_{H_{2}O_{2}}$ and a high $E_{{@1\;\mathrm{mA}\;\mathrm{cm}}^{-2}}$ which is the best‐performing 2 e^−^ ORR electrocatalyst reported to date, and approaches the theoretical thermodynamic limit. ECD analysis suggests manipulation of selectivity is attainable without sacrificing activity. Through *operando* spectroscopic studies, the excellent 2 e^−^ ORR activity and selectivity can be attributed to the optimised O_2_ hydrogenation kinetics of the CoN_4+4_ active centre, validating the as‐proposed hypothesis. Finally, a redox‐induced electron delocalisation mechanism was identified via analysing the pseudocapacitive and ORR behaviours of the CoN_4+4_‐ACNT and CoN_2+*x*_‐ACNT samples. Such a mechanism was then applied for regulating the H_2_O_2_ formation of 4 e^−^ ORR catalysts. The above discoveries provide fresh insights for electrocatalyst design in both fuel cell and H_2_O_2_ generation applications.

## Conflict of interest

There is no conflicts of interests to declare.

## Supporting information

As a service to our authors and readers, this journal provides supporting information supplied by the authors. Such materials are peer reviewed and may be re‐organized for online delivery, but are not copy‐edited or typeset. Technical support issues arising from supporting information (other than missing files) should be addressed to the authors.

Supporting Information
