# Supplementary material for: Approaching Theoretical Performances of Electrocatalytic Hydrogen Peroxide Generation by Cobalt‐Nitrogen Moieties
Source: Angew Chem Int Ed Engl. 2023 Apr 18;62(21):e202301433. doi: 10.1002/anie.202301433 (PMC10962607; doi:10.1002/anie.202301433)
Supplement: Supplementary file 1 — Supporting Information [file ANIE-62-0-s001.pdf]

Angewandte Chemie
Eine Zeitschrift der Gesellschaft Deutscher Chemiker

Supporting Information

Approaching Theoretical Performances of Electrocatalytic Hydrogen Peroxide Generation by Cobalt-Nitrogen Moieties

*R. Lin, L. Kang, K. Lisowska, W. He, S. Zhao, S. Hayama, G. J. Hutchings, D. J. L. Brett, F. Corà, I. P. Parkin, G. He**

Supplementary Information

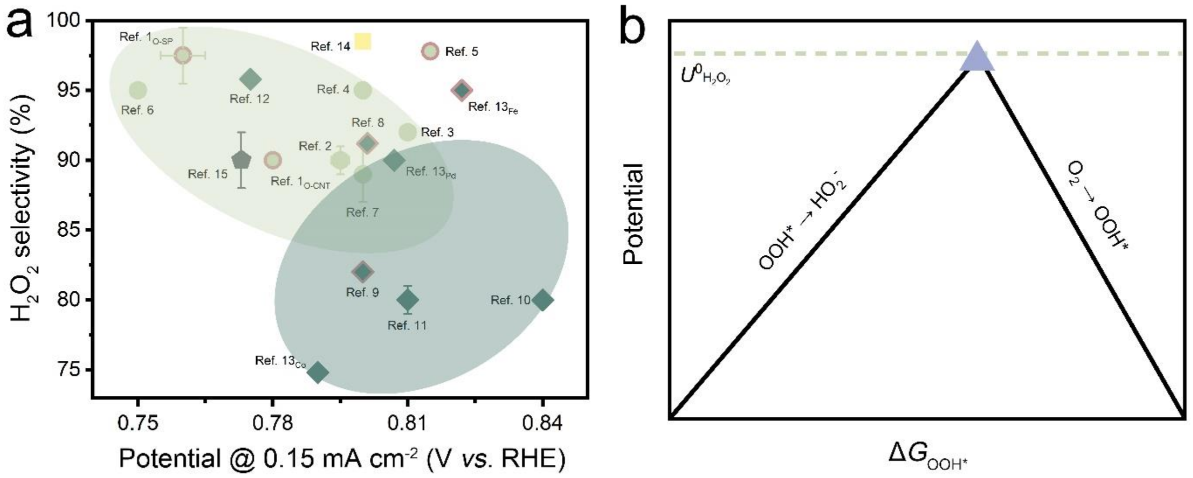

Supplementary Figure 1 a Comparison of the onset potential (the potential at which ring current density reaches 0.15 mA cm^{-2}) and corresponding selectivity for H_2O_2 electrosynthesis on recently-reported benchmark electrocatalysts (in alkaline medium).[1-15] Dark green diamonds represent metal-based catalysts; light green circles, yellow squares and grey pentagons represent metal-free oxygen-functionalized, nitrogen-doped and boron-doped carbon materials, respectively. Note that Ref. 1-15 in the supplementary information correspond to Ref. 11-25 in the manuscript. **b** Schematic diagram of a volcano plot used for designing 2e^- ORR catalyst as seen in many previous works. The green dash line represents the 2e^- ORR equilibrium potential. The dots with pink outer shells in Supplementary Figure 1a represent the catalysts that claim the reaching of the volcano peak (blue triangle in Supplementary Figure 1b).

The electrochemical reduction of O_2 to H_2O_2 consists of the hydrogenation of O_2 ($O_2 + H_2O + e^- \rightarrow OOH^* + OH^-$) and the reduction of OOH^* to HO^{2-} ($OOH^* + e^- \rightarrow HO_2^-$).[1, 9] On the basis of the Sabatier principle, the adsorption energy of the OOH^* intermediate should be neither too strong (otherwise the reaction is limited by the secondary desorption step) nor too weak (otherwise the reaction is limited by the elementary adsorption step) to optimize the reaction kinetics. Hence, lifting catalysts to the volcano peak (blue triangle in Supplementary Figure 1b) becomes a moral law for current 2e^- ORR catalyst design.[1, 5, 9, 15] In other words, in most of the literature, attention is mainly paid on the adjustment of the OOH^* adsorption energy.

Supplementary Table 1 Comparisons of alkaline (0.1 M KOH) 2e$^-$-ORR performance (measured by RRDE system) of recently reported state-of-the-art electrocatalysts.

Sample	Active site	Metal loading	E_{onset} (V *vs.* RHE)	H_2O_2 selectivity (%)	$E_{@1\ mA\ cm^{-2}}$ (V *vs.* RHE)	H_2O_2 selectivity (%)	Reference
**CoN_{4+4}-ACNT**	Co-N_x	0.105 wt%	0.857	~100	0.82	~100	This work
**O-CNT**	O dopant	N.A.	0.78	90	0.70	90	Ref. 1
**O-SP**	O dopant	N.A,	0.76	97.5	0.67	93	Ref. 1
**GOMC**	O dopant	N.A.	0.80	~90	0.75	~90	Ref. 2
**O-GOMC**	O dopant	N.A.	0.81	~92	0.72	~93	Ref. 3
**CB+CTAB**	O dopant	N.A.	0.8	~95	0.765	~90	Ref. 4
**GNO_{C-O-C}**	O dopant	N.A,	0.815	97.8	0.77	97	Ref. 5
**N-O-P-C**	O, N, P dopants	N.A.	0.75	~95	0.63	~95	Ref. 6
**CMK3**	O dopant	N.A.	0.8	~89	0.6	~90	Ref. 7
**Co-N_2-C/HO**	Co-N_x-O	0.88 wt%	0.801	91.2	0.76	91	Ref. 8
**Co_1-NG (O)**	Co-N_x-O	1.4 wt%	0.8	82	0.72	79	Ref. 9
**Co–POC–O**	Co-N_x-O	0.7 at%	0.84	80	0.78	84	Ref. 10
**NiN_x/C-$AQNH_2$**	Ni-N_x	1.82 wt%	0.81	80	0.71	83	Ref. 11
**Mo_1/OSG-H**	Mo-S-O	13.47 wt%	0.775	95.8	0.74	95	Ref. 12
**Co-CNT**	Co-O	0.1 at%	0.79	74.8	0.7	75	Ref. 13
**Pd-CNT**	Pd-O	0.1 at%	0.807	90	0.75	87.5	Ref. 13
**Fe-CNT**	Fe-O	0.1 at%	0.822	95	0.77	95.4	Ref. 13
**N-mFLG-8**	Pyrrolic N dopant	N.A.	0.8	98.5	0.685	95	Ref. 14
**B-C**	B dopant	N.A.	0.773	90	0.68	83	Ref. 15

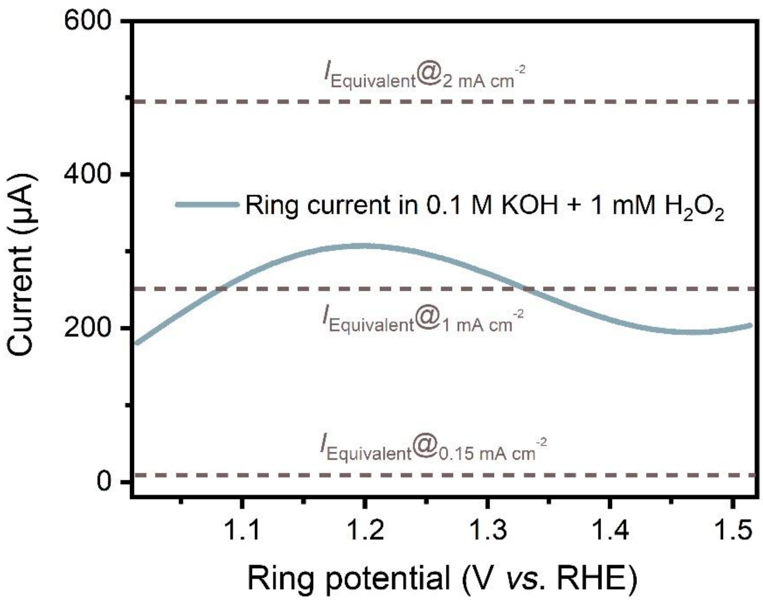

Supplementary Figure 2 Ring current recorded in N_2-saturated 0.1 M KOH containing 1 mM H_2O_2 at 1600 rpm (disk was disconnected throughout the test) and the calculated equivalent ring current ($I_{Equivalent}$: a hypothetical ring current which is calculated by assuming all of the disk-generated H_2O_2 can be detected and collected by the ring electrode, brown dash lines) at which the actual ring current density of a rotating ring-disk electrode (RRDE) would reach 0.15, 1 and 2 mA cm^{-2} in practical ORR tests.

It is noteworthy that 89% of the works in Supplementary Figure 1a claim that their catalysts show an onset potential higher than the thermodynamic limit of the 2e$^-$ ORR (O_2 + H_2O + 2e$^-$ → HO^{2-} + OH^-, E^0 = 0.76 V *vs.* RHE at 298 K, pKa_1 = 11.69 and pKa_2 = 20) which can be ascribed to the potential shift according to Nernst equation (due to lower concentration of the H_2O_2 product and higher O_2 partial pressure compared to standard condition). Hence, to comprehensively evaluate a catalyst, it is important to compare its electrocatalytic performance with the thermodynamic limits that are calculated at practical conditions. According to the McCloskey group's result, $E = E^0 - \frac{0.059}{2} \times \log\left(\frac{[HO_2^-][OH^-]}{p_{O_2}}\right)$.[9]

It is also vital that attention is paid to the catalytic performance not only near the onset potential (at which the H_2O_2 generation rate is relatively low), but also under operation conditions similar to that in practical devices. Hence, assuming a product (H_2O_2) concentration of 1 mM (equivalent to the disk-generated H_2O_2 is 1 mM near the disk electrode at the time of its production before its diffusion to the whole electrolyte), electrolyte pH = 13 and O_2 partial pressure of 800 torr, the thermodynamic ORR 2e$^-$ potential is calculated as 0.825 V *vs.* RHE.[16] To correlate the as-assumed 1 mM H_2O_2 production rate with the ring current density in practical ORR tests, the ring electrode of a bare RRDE is scanned in the electrolyte containing 1 mM H_2O_2 and the result is presented in Supplementary Figure 2. It is found the ring current obtained in 1 mM H_2O_2 is similar to the $I_{Equivalent}$ of a 1 mA cm^{-2} ring current density monitored in a practical RRDE test (Supplementary Figure 2). Please note the $I_{Equivalent}$ is calculated by the following equation:

$$I_{Equivalent} = I_{ring}/N_c$$

Where I_{ring} represents the monitored ring current in an ORR RRDE test, N_c represents the collection efficiency of the RRDE. The instant H_2O_2 concentration near the disk electrode when the ring electrode current density reaches 1 mA cm^{-2} is approximately 1 mM. Therefore, the thermodynamic disk potential for reaching a 1 mA cm^{-2} ring current density is 0.825 V *vs.* RHE.

Supplementary Note 1

Figure 1c (in the manuscript) demonstrates the $\Delta E_{@1\ mA\ cm^{-2}}$ (the voltage required to lift the $2e^-$ ORR current density to 1 mA cm^{-2} since the reaction onset) of the electrocatalysts displayed in Figure 1a (in the manuscript) and Supplementary Figure 1a. It can be found that samples (*i.e.* ref. 15, 18 and 22 in Figure 1a which correspond to ref. 5, 8 and 12 in Supplementary Figure 1a) must have both good activity and selectivity (> 90%), should they exhibit small $\Delta E_{@1\ mA\ cm^{-2}}$ (< 50 mV). Samples with high selectivity but poor activity (*i.e.* ref. 16 and 24 in Figure 1a which correspond to ref. 6 and 14 in Supplementary Figure 1a) show large $\Delta E_{@1\ mA\ cm^{-2}}$ due to the uncoordinated O_2 hydrogenation and OOH^* desorption collaboration. It is worth pointing out that some metal-based catalysts with excellent onset still display large $\Delta E_{@1\ mA\ cm^{-2}}$ (*i.e.* ref. 17, 21 and 25 in Figure 1a which correspond to ref. 7, 11 and 15 in Supplementary Figure 1a). This can possibly be attributed to the diverted OOH^* stream at the desorption/dissociation step of their $2e^-$ ORR process.

An ideal $2e^-$ ORR electrocatalyst should meet the following criteria: first, an optimal ΔG_{OOH^*} for facile O_2 to H_2O_2 conversion; second, a favourable OOH^* desorption kinetics over that of OOH^* dissociation. In practice, the two criteria normally cannot be satisfied at the same time. From the discussion in Fig. 1 (in the manuscript) and Supplementary Note 1, one can tell that optimizing the trade-off between the reaction rate of $^*O_2$ hydrogenation, OOH^* desorption and OOH^* dissociation is a more efficient and doable strategy for a rational $2e^-$ ORR electrocatalyst design.

Methodology

Chemicals. Cobalt(II) acetate tetrahydrate ($Co(CH_3COO)_2{\cdot}4H_2O$, ≥98%), pyrrole ($C_4H_5N$, ≥98%), cobalt(II) phthalocyanine ($C_{32}H_{16}CoN_8$, β-form, dye content 97%), 2,3,7,8,12,13,17,18-octaethyl-21*H*,23*H*-porphine cobalt(II) ($C_{36}H_{44}CoN_4$, dye content 85%), carbon nanotube (multi-walled), nano carbon (nanopowder, <100 nm particle size), tannic acid (ACS reagent), urea (99.0-100.5%), boric acid (ACS reagent, ≥99.5%), commercial Pt/C (20 wt% Pt loading), absolute ethanol (99.8%), hydrogen peroxide (H_2O_2, 30% (w/w) in H_2O, contains stabilizer), hydrochloric acid (HCl, ACS reagent, 37%), concentrated nitric acid (HNO_3, 65%) and Nafion 117 containing solution (~5%) were purchased from Sigma Aldrich. Potassium hydroxide (KOH, 85%) was purchased from Fisher Scientific. Hydrophilic carbon paper (TGP-H-060) was purchased from Toray.

Synthesis of ACNT and OCNT. To manufacture ACNT, multi-walled CNT (5 g) was calcinated at 500 °C (ramp rate: 10 °C min^{-1}) under N_2 flow for 1h. The powder was then boiled in 50 mL 10% hydrochloric acid for 30 min at 90 °C. After overnight freeze-dry, ACNT was obtained. OCNT was fabricated by boiling ACNT (5 g) in 50 mL 3 M nitric acid at 80 °C for 12 h. After overnight freeze-dry, OCNT was obtained.

Synthesis of CoN_x-carbon sample series. CoN_{4+4}-ACNT was manufactured by firstly mixing 2 mg ACNT and 4 mg cobalt phthalocyanine in 30 mL absolute ethanol. The mixture was tip-sonicated for 15 min and then stirred at room temperature for at least 2 days. CoN_{4+4}-ACNT was finally obtained after centrifuging at 4500 rpm (until a clear supernatant was observed)

and freeze-drying for overnight. CoN_4-ACNT was synthesised *via* a similar method except the use of cobalt porphine instead of cobalt phthalocyanine. To synthesise CoN_{2+x}-ACNT, a cobalt precursor solution was first manufactured by dissolving 0.5 g $Co(CH_3COO)_2 \cdot 4H_2O$ and 560 µL pyrrole in 50 mL absolute ethanol. The solution was then transferred into a 100 mL autoclave (before any synthesis, the autoclave was cleaned by boiling in concentrated nitric acid overnight and washed several times by DI water). After hydrothermal treatment at 120 °C for 12 h, the resulted solution was centrifuged at 4500 rpm and the supernatant was collected as the cobalt precursor for CoN_{2+x}-ACNT synthesis. Then, 2 mg ACNT was added into the as-prepared precursor solution and tip-sonicated for 15 min. After stirring at room temperature for at least 2 days, the solution was centrifuged at 4500 rpm (until a clear supernatant was observed). The precipitate was then boiled in 10% hydrochloric acid for 5 min at 70 °C to remove any residue metal species. After centrifuge and free-dry treatment, CoN_{2+x}-ACNT was obtained. Other CoN_x-carbon hybrids were prepared following the corresponding similar methods except the use of different carbon materials.

Synthesis of *x*OCoN$_4$-ACNT sample series. *x*OCoN$_4$-ACNTs were prepared by boiling cobalt porphine (4 mg) in 50 mL 3 M nitric acid for different period of time (*x* stands for treatment time in hour). The precursors were then mixed with 2mg of ACNT and stirred for at least 2 days. After centrifuge and free-dry treatment, the products were obtained.

Synthesis of BNC. BNC was fabricated by dissolving 0.6 g boric acid, 0.4 g urea and 5 g tannic acid in 10 mL Milli-Q deionized (DI) water (18.2 MΩ•cm). After free-drying, the loose powder was annealed at 850 °C (ramp rate: 10 °C min^{-1}) under N_2 atmosphere for 2 h and the product was denoted as BNC.

Synthesis of CoN$_x$-ACNT@Co$_3$O$_4$ sample series. To prepare a Co_3O_4 precursor solution, 0.5 g $Co(CH_3COO)_2 \cdot 4H_2O$ was first dissolved in 50 mL absolute ethanol. The solution was then transferred into a 100 mL autoclave. After hydrothermal treatment at 120 °C for 12 h, the resulted solution was centrifuged at 4500 rpm and the supernatant was collected as the Co_3O_4 precursor. The hybridisation of CoN_x-ACNT with Co_3O_4 was accomplished by stirring the CoN_x-ACNT samples (2 mg) in the Co_3O_4 precursor solution at room temperature for at least 2 days. After centrifuging and free-drying treatment, the product was finally obtained.

Electrochemical measurements. Electrochemical tests were carried out using a three-electrode configuration with a RRDE set-up (Pine Instruments Corporation) and Gamry Interface 1010E potentiostat (unless specified). Prior to measurement, the RRDE was polished mechanically with alumina suspension and rinsed with DI water. A graphite rod and a Ag/AgCl electrode with saturated KCl filling solution were used as the counter and reference electrode, respectively. The working electrode was prepared as follows. 1 mg of the catalyst was first dispersed in 500 µL solution that contained 495 µL ethanol and 5 µL 5% Nafion solution. After tip sonication for 15 min, 12 µL of the ink was dropped (6 µL each time) onto the disk (0.2475 cm^2) of the RRDE (Au and Pt rings were used for measurement in alkaline and neutral electrolytes, respectively). All potentials were converted to the RHE scale and *iR*-corrected (95%) by the resistance of the electrolyte unless specified. Conversion between the potentials *vs.* RHE and *vs.* saturated Ag/AgCl was performed by the following equation: E (*vs.* RHE) = E (*vs.* Ag/AgCl) + $E_{Ag/AgCl}$ (*vs.* standard hydrogen electrode (SHE)) + 0.059 × pH. At 25 °C, $E_{Ag/AgCl}$ (*vs.* SHE) = 0.197 V *vs.* SHE. Current densities for all electrochemical measurements were normalised to the geometric surface area of the corresponding current collectors used for their respective electrochemical measurement. Electrochemical impedance spectra were recorded at open-circuit potential under 10 mV of amplitude from 100 kHz to 0.1 Hz. LSV and CV were performed at a scan rate of 5 mV s^{-1} unless specified. Before the ORR measurement, the working electrode was cycled from 0.164-0.964 V *vs.* RHE (scan rate: 20 mV s^{-1}) with continuous N_2 flow purging into the electrolyte until a stable CV shape was obtained; the ring

of the RRDE was activated by biasing at 0.1 V *vs.* RHE for 60 s. Then, the ORR activity and selectivity were obtained from polarization curves in O_2-saturated conditions between 0.1 and 1.0 V at a scan rate of 5 mV s^{-1} and rotating rate of 1600 rpm while holding the potential of the Au ring electrode at 1.2 V *vs.* RHE or the Pt ring at 1.35 V *vs.* RHE. The ORR current was corrected by subtracting the current obtained in a N_2-saturated electrolyte from that measured in O_2-saturated conditions. All the measured ring currents were corrected using collection efficiency (N) of the RRDE set-up to obtain the overall current density as all the generated H_2O_2 was detected. The ring collection efficiency was calibrated as 0.375 for the Au ring RRDE and 0.385 for the Pt ring RRDE by the redox reaction of $[Fe(CN)_6]^{4-}/[Fe(CN)_6]^{3-}$. The $FE_{H_2O_2}$, H_2O_2 selectivity and n were calculated using the following equations: $FE_{H_2O_2}$ (%) = $100 \times \frac{j_{ring}}{j_{disk}}$, H_2O_2 selectivity (%) = $200 \times \frac{j_{ring}}{j_{disk} + j_{ring}}$, $n = 4 \times \frac{j_{disk}}{j_{disk} + j_{ring}}$. Where j_{disk} and j_{ring} represent the normalised disk and ring current density ($j_{disk} = \frac{I_{disk}}{A_{disk}}$; $j_{ring} = \frac{I_{ring}}{A_{disk} \times N_c}$). For bulk H_2O_2 generation characterization, a H-cell configuration was employed (catalyst was coated onto a carbon paper with a loading of 1mg cm^{-2}), a Ce^{4+} titration method was used to quantitatively analyse the produced H_2O_2 (more details can be found in Supplementary Figure 13 and ref. 14).

Physicochemical characterization. The XRD patterns were obtained using a STOE SEIFERT diffractometer with a Mo X-ray radiation source. The HAADF-STEM were taken on a JEOL JEM ARM200F microscope. The XPS measurements were performed on a Thermo scientific K-alpha photoelectron spectrometer. Data processing of XPS results were achieved by Casa XPS with calibration of adventitious carbon binding energy at 284.8 eV. Microwave plasma atomic emission spectrometry (MP-AES, 4210 Agilent Technologies) was employed to determine the Co mass loading (samples were first annealed in the quartz tube at 800 °C in air for 3 h; the residue was then dissolved into 10 mL 4 wt% nitric acid solution; standard samples including 0, 0.4, 0.8, 1.2, 1.6 and 2.0 ppm were used for linear calibration).

X-ray absorption spectroscopy. The NEXAFS measurement was performed at B07-B beamline of Diamond Light Source.[17] Co $L_{3,2}$-edge and O K-edge NEXAFS spectra were acquired in total electron yield (TEY) mode with the ES-2 endstation (designed for ambient pressure NEXAFS measurement). The catalysts powder and reference materials were dispersed on Indium film by pressing, which ensured good conductivity and prevented sample contamination. The samples were illuminated by incident beam sourced from a bending magnet and plane grating monochromator (PGM) with a spot size of ~200 × 200 μm. The pressure in the specimen chamber is controlled at 1×10^{-7} mbar. For each sample at each absorption edge, 3 repetitions of NEXAFS spectrum were collected and merged to improve the signal-to-noise ratio. The Co K-edge XAFS measurements were conducted at the B18 beamline of Diamond Light Source.[18-19] A quick extended X-ray absorption fine structure (QEXAFS) mode was set-up though a fast-scanning Si(111) double crystal monochromator and Pt-coated branch of collimating and focus mirrors. A couple of Pt-coated harmonic rejection mirrors were inserted to suppress the unfocused high energy 3rd and higher harmonics. XAFS data were acquired in transmission mode under room temperature using ionisation chambers. Co catalysts were diluted with boron nitride and pressed into pellets (8 mm diameter). Three scans of each sample were acquired and merged to improve the signal to noise ratio. For each single scan, the energy range was from 7509-8559 eV with a step size of 0.3 eV. Co foil was measured simultaneously for energy calibration by shifting the maximum of the first derivative of Co foil XAFS spectrum to 7709 eV, which is defined to be the absorption edge position of metallic cobalt. XAFS data were analysed using the Demeter software package (version 0.9.26, including Athena and Artemis software).[20] The Athena software was used to subtract the background and normalise the edge jump. The k^3-weighted $\chi(k)$ data were Fourier-transformed to R space using a Hanning

window function (3<k<13, Δk=1). The Fourier-Transformed R-space EXAFS spectra were fitted using Artemis software (1<R<3.2). The amplitude reduction factor was calculated to be 0.9 through the fitting of Co foil EXAFS spectrum, and this value was used as a constant for the EXAFS fitting of all the Co catalysts. High-energy-resolution fluorescence detection (HERFD)-XANES measurements were carried out at the I20-Scanning beamline of Diamond light source.[21-22] The incident beam energy was monochromatized using a Si(111) 4-bounce scanning monochromator[23]. The Co K_{β} X-ray emissions were analysed using three Ge(444) spherical analysers operated in a 1 meter diameter Rowland circle Johann configuration. HERFD-XANES spectra for each sample were measured by monitoring the intensity of $K_{\beta1,3}$ emission line (fixed to the peak emission intensity measured with the incident energy at 8000 eV. The peak position shifted between 7649.5 and 7650.5 eV depending on the sample) as a function of incident beam energy (between 7600 and 8000 eV, step size = 0.3 eV). A Co foil was measured for calibrating the energy of the monochromator and XES spectrometer and an elastic peak was measured to estimate the energy resolution of measurements, approx. 1.2eV FWHM. Analysis of HERFD-XANES data including background subtraction and normalisation are performed using the xraylarch package (version 0.9.58).[24] See Supplementary Figure 23 for more details of the *operando* measurement.

Computational methods. Density functional theory (DFT) calculations on the adsorption structure of the catalysts (Supplementary Figure 25) were performed using Vienna ab initio simulation package (VASP) with the basis generated by projector augmented waves (PAW).[25] The Perdew, Burke and Ernzerhof (PBE)[26] functional was used for the description of the exchange and correlation potential. Only gamma point was used in the reciprocal zone. The criteria of convergence were set to 1×10^{-5} eV and 0.02 eV $Å^{-1}$ for electronic and ionic steps, respectively. The DFT-D3 method of Grimme was adopted to describe vdW dispersion.[27] The models of porphyrin and phthalocyanine were used for CoN_{4+4} and CoN_4, respectively. The model contains a vacuum layer of 15 Å to avoid the interaction between periodic images.

The computational study on the electronic structure of the catalysts (Figure 5, Supplementary Figure 26, Supplementary Table 17 and Supplementary Note 4) was carried out using DFT implemented in the CRYSTAL17 code. The calculations were carried out using the B3LYP functional augmented with the D3 dispersion correction, calculated as the sum of the two- and three-body contributions. Atomic orbital Gaussian basis sets available from the CRYSTAL online database were selected and are indicated by the following labels: N_m-6-311G(d)_Heyd_2005, C_m-6-311G(d)_Heyd_2005, O_m-6-311G(d)_Heyd_2005, H_3-1p1G_gatti_1994, Co_86-411d41G_towler_1992. Non-standard 7 7 7 9 30 TOLINTEG truncation were applied to ensure smooth numerical convergence. Reciprocal space has been sampled using a Pack-Monkhorst net with the shrinking factor of 12 for CoN_x molecules and shrinking factor of 1 for complexes deposited on the 8×8 supercell of the pristine graphene, N doped graphene and graphene oxide supports. The model for CoN_x complex deposited on the support is a structure with periodic boundary conditions in two dimensions with the 8×8 supercell expansion of the Graphene cell consisting of 128 carbon atoms. Spin polarised calculations have been performed for all systems with unpaired electrons, fixing the required number of unpaired electrons for the first 50 self-consistent field (SCF) cycles to enforce the desired electronic state (Supplementary Table 17).

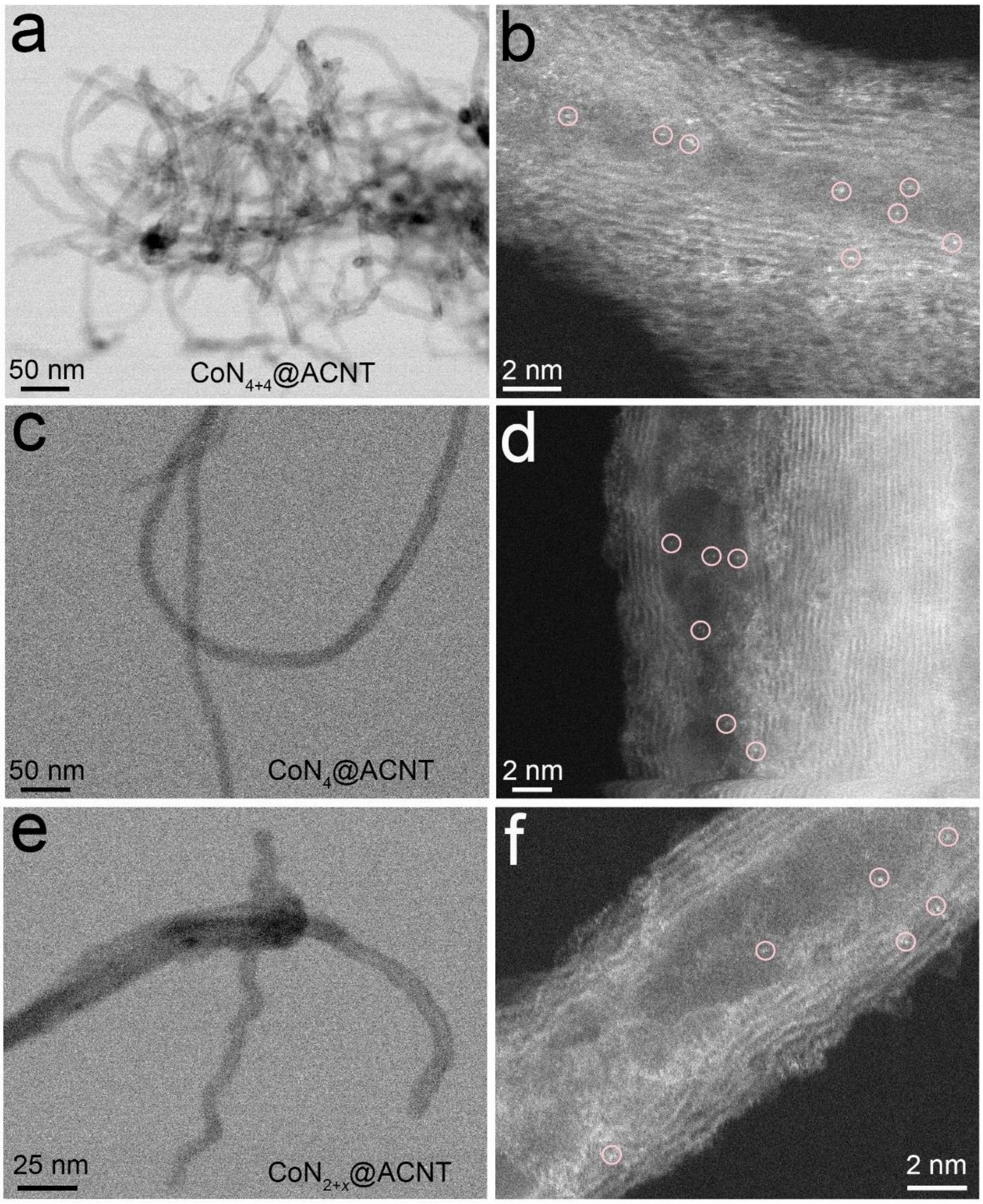

Supplementary Figure 3 a, b (**a**) Scanning transmission electron microscopy (STEM) and (**b**) high-angle annular dark-field scanning transmission electron microscopy (HAADF-STEM) images of CoN_{4+4}-ACNT. **c, d** (**c**) STEM and (**d**) HAADF-STEM images of CoN_4-ACNT. **e, f** (**e**) STEM and (**f**) HAADF-STEM images of CoN_{2+x}-ACNT. The bright dots highlighted with pink circles are believed to be cobalt single atoms.

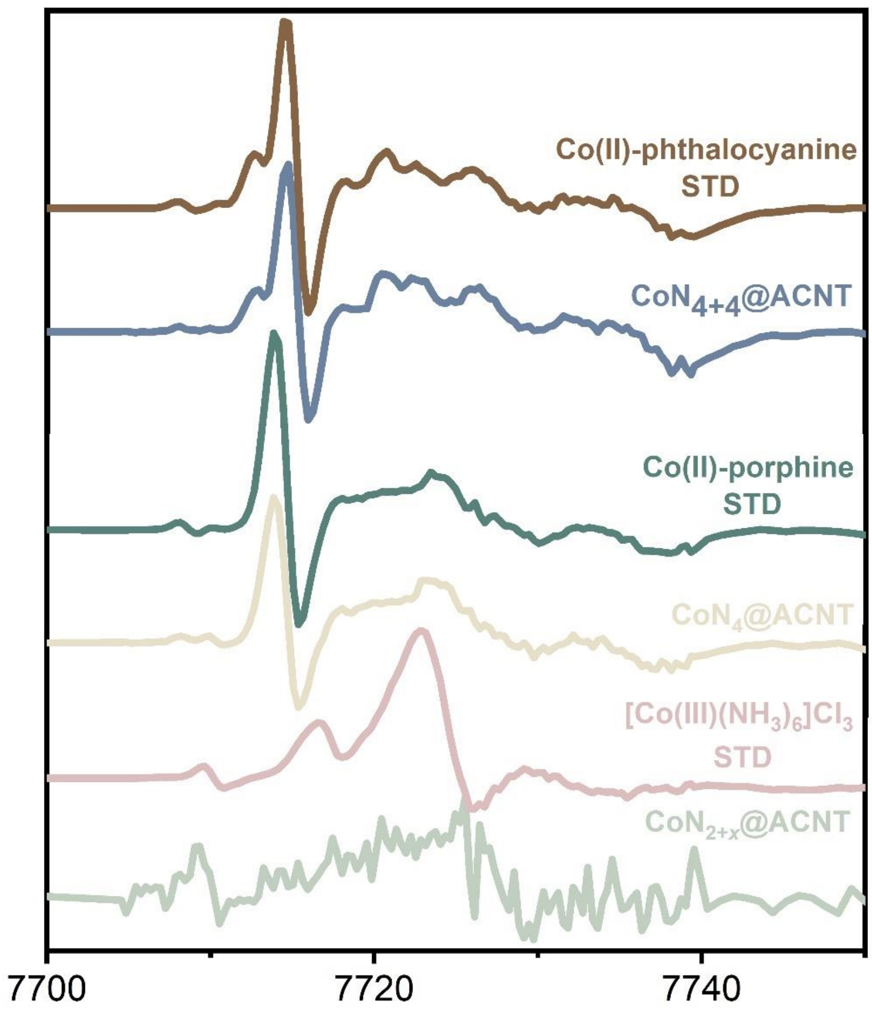

Supplementary Figure 4 First derivative Co K-edge HERFD-XANES spectra of CoN_x-ACNT and reference samples (derived from Figure 2d in the manuscript).

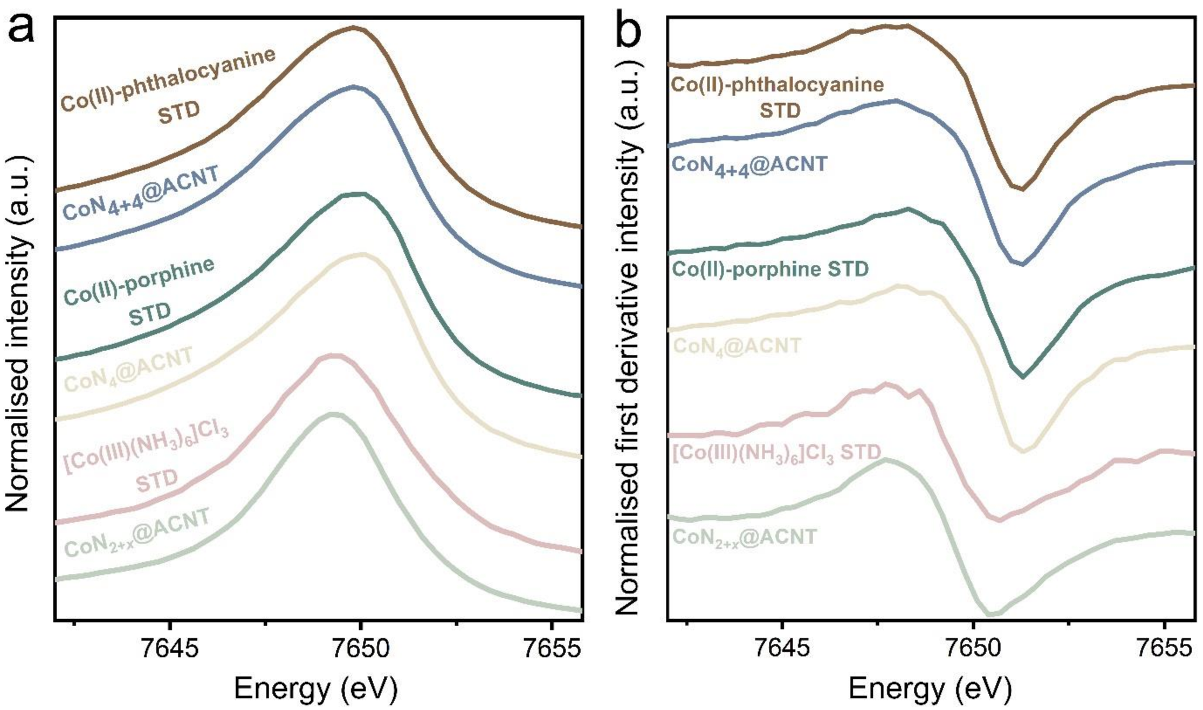

Supplementary Figure 5 a Co Kβ X-ray emission spectroscopy (XES) and **b** first derivative XES of CoN_x-ACNT and reference samples.

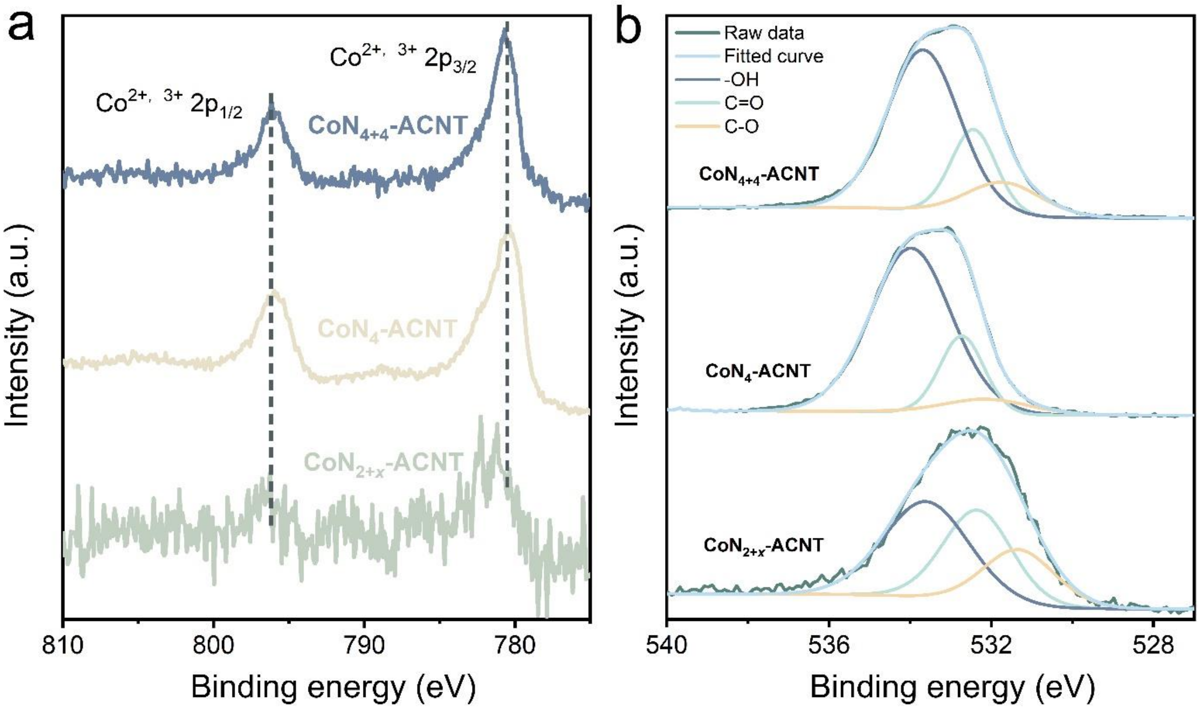

Supplementary Figure 6 X-ray photoelectron spectroscopy (XPS) (**a**) Co 2p and (**b**) O 1s spectra of CoN_x-ACNT samples. The XPS Co 2p characteristic peaks of CoN_{2+x}-ACNT shift to higher binding energy when referenced to CoN_{4+4}-ACNT and CoN_4-ACNT, revealing a

higher Co oxidation state. No sign of metal-O bonding can be observed in the XPS O 1s spectra. The characteristic peaks in Supplementary Figure 6b can be attributed to oxygen species of the CNT substrate. It is noteworthy that the stronger C=O and C-O signals of the CoN_{2+x}-ACNT sample can possibly be ascribed to the residue acetyl group of the cobalt(II) acetate precursor.

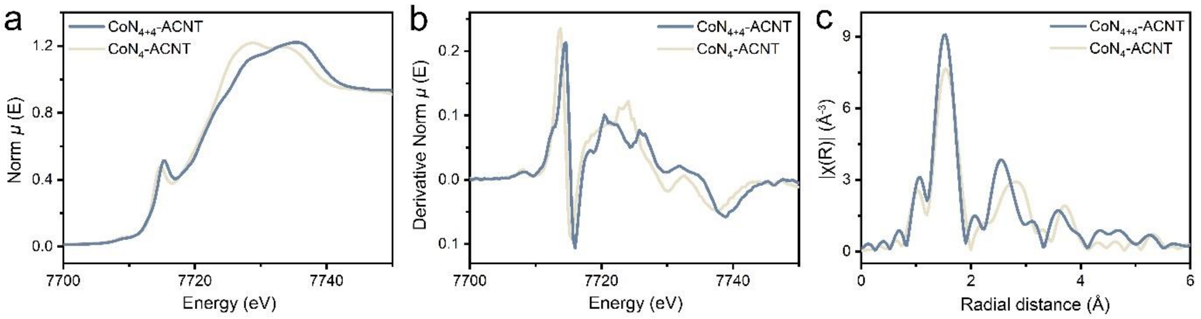

Supplementary Figure 7 Co K-edge X-ray absorption fine structure (XAFS) spectra of CoN_x-ACNT sample series. **a** X-ray absorption near edge structure (XANES), **b** second derivative XANES and **c** k^2-weighted R-space extended X-ray absorption fine structure (EXAFS) of CoN_{4+4}-ACNT and CoN_4-ACNT.

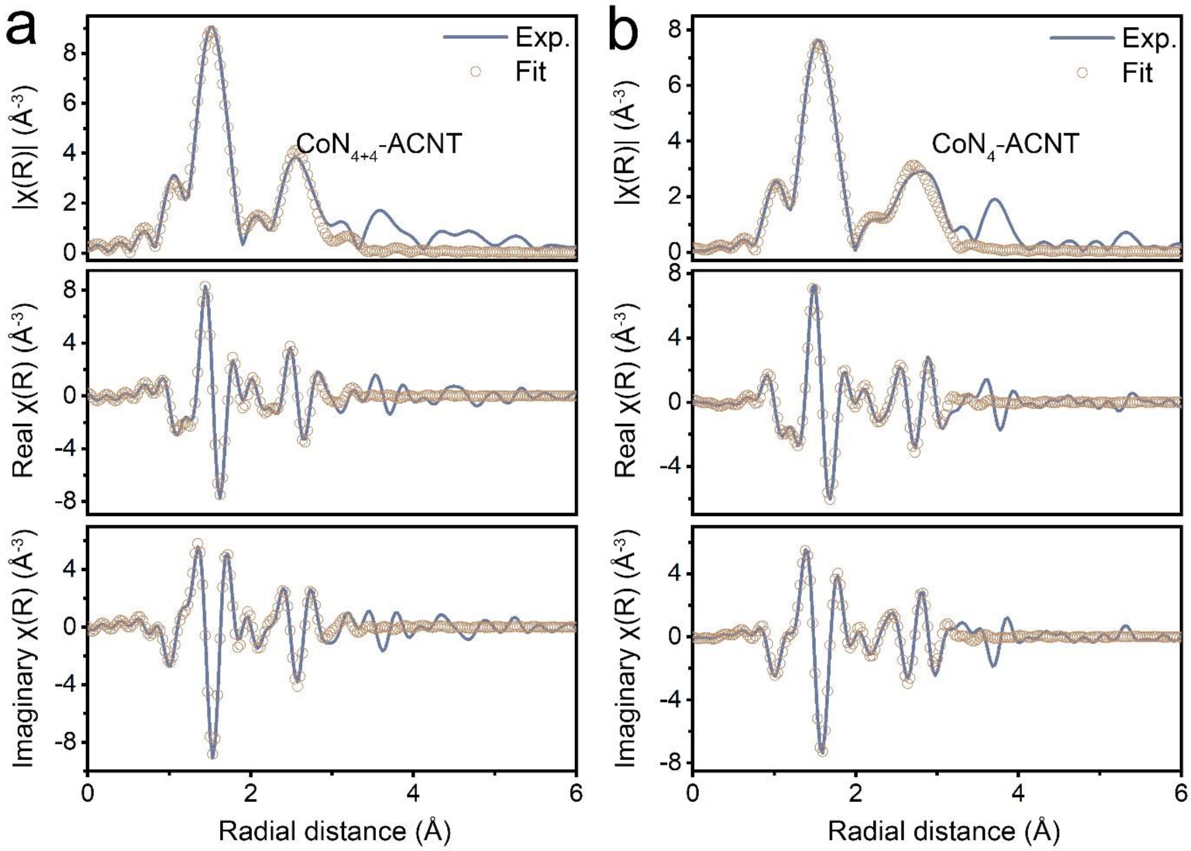

Supplementary Figure 8 Fitting results of Co K-edge EXAFS spectra of **a** CoN_{4+4}-ACNT and **b** CoN_4-ACNT. The raw experiment data are plotted in circles, and the fitting results are plotted as curves. The corresponding fitting parameters are presented in Supplementary Table 1.

Coordination structures of CoN_{4+4}-ACNT and CoN_4-ACNT are revealed by EXAFS fitting to differ their atomic structures. Considering their high energy resolution fluorescence detected (HERFD)-XANES and near edge X-ray absorption fine structure (NEXAFS) spectra are similar to molecular Co(II)-phthalocyanine and Co(II)-porphine (Figure 2c and d in the manuscript); and the moiety anchoring procedure did not involve any extreme conditions such as high-temperature pyrolysis, the EXAFS fitting was performed based on the hypothesis that the geometric structure of the molecular precursors were well preserved. It is worth mentioning that C, N and O atoms are very similar scatterers for X-rays and cannot be distinguished by fitting the EXAFS spectra regardless of coordination distances. Three single scattering paths are evaluated based on the corresponding molecular complexes to fit the experiment data, including (1) the four direct bonding pyrrolic N atoms, (2) the eight carbon atoms next to the pyrrolic N, and (3) the four C or N atoms that bridge the four pyrrolic ligands. Both catalysts have first shell coordination of Co-N with a coordination number of 3.9 (CoN_{4+4}-ACNT) and 4.2 (CoN_4-ACNT). The Co-N bond length in CoN_{4+4}-ACNT was calculated to be 1.91 Å, which is slightly shorter than that in CoN_4-ACNT (1.98 Å) and in line with their corresponding complexes.[28] CoN_{4+4}-ACNT has a second shell N coordination number of 3.9. Despite the slight variations from fitting errors, both the coordination numbers and the interatomic distances of the second shell Co-C and the third shell Co-C/N in the two catalysts are in good agreement with the molecular complexes.

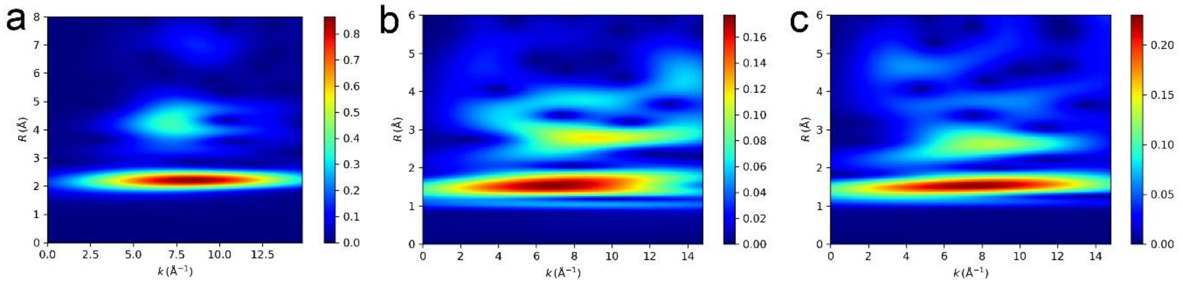

Supplementary Figure 9 Co K-edge k^3-weighted wavelet transform EXAFS (WT-EXAFS) of **a** Co foil, **b** CoN_4-ACNT and **c** CoN_{4+4}-ACNT.

Supplementary Table 2 Fitting parameters of Co K-edge EXAFS of CoN_{4+4}-ACNT and CoN_4-ACNT.

**Sample**		**CoN_{4+4}-ACNT**	**CoN_4-ACNT**
**amp**		0.9	0.9
**ΔE_0 (eV)**		0.8±2.0	1.8±2.4
**Co-N(1)**	**C.N.**	3.9±0.4	4.2±0.5
	**R (Å)**	1.91±0.01	1.98±0.01
	**σ^2**	0.003±0.001	0.004±0.001
**Co-C(1)**	**C.N.**	7.9±0.9	8.3±1.0
	**R (Å)**	2.94±0.01	3.02±0.01
	**σ^2**	0.004±0.001	0.005±0.002
**Co-N(2)**	**C.N.**	3.9±0.4	N.A.
	**R (Å)**	3.29±0.03	N.A.
	**σ^2**	0.007±0.003	N.A.
**Co-C(2)**	**C.N.**	N.A.	4.2±0.5
	**R (Å)**	N.A.	3.35±0.02
	**σ^2**	N.A.	0.006±0.002
**R-factor**		0.016	0.021

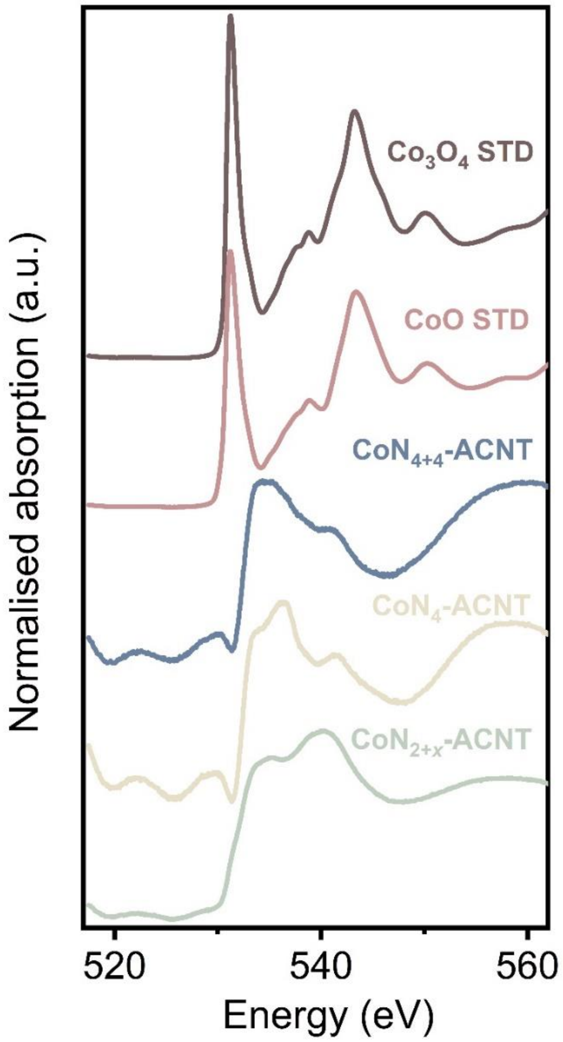

Supplementary Figure 10 O K-edge NEXAFS spectra of CoO, Co_3O_4 and CoN_x-ACNT. CoN_x-ACNT sample series share similar O K-edge NEXAFS spectra. It is believed the O signals come from the O-functional groups of the ACNT substrates. No metal-O peak can be detected which agrees with the XPS analysis.

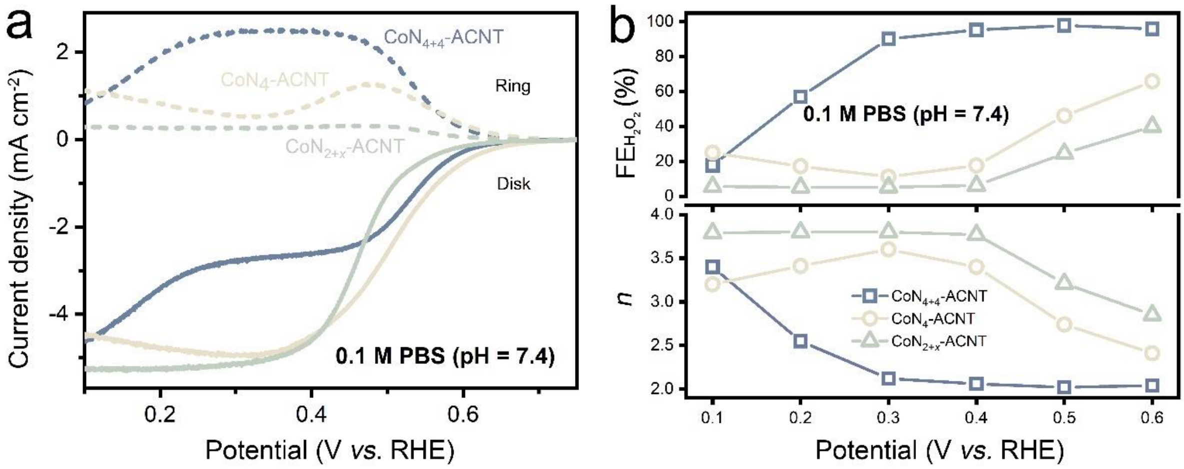

Supplementary Figure 11 a Comparison of ORR performance of the CoN$_x$-ACNT series catalysts at 1600 rpm in O_2-saturated 0.1 M potassium buffer solution (PBS, pH = 7.4) and **b** the calculated H_2O_2 production faraday efficiency ($FE_{H_2O_2}$) and electron transfer number (*n*) as a function of the applied potential. More details are available in Supplementary Table 12-14.

As can be learned from Supplementary Figure 11, the selectivity trend of CoN$_x$-ACNT series catalysts examined in neutral electrolyte is similar to the results observed in alkaline medium. CoN$_{4+4}$-ACNT exhibits the highest $FE_{H_2O_2}$ (over 95% in a wide potential window from 0.4 to 0.6 V *vs.* RHE). The decreased $FE_{H_2O_2}$ in the low-potential region (0.1-0.4 V *vs.* RHE) is induced by the Co^+/Co^{2+} redox chemistry of CoN$_{4+4}$ moiety which will be discussed in detail later. CoN$_4$-ACNT shows a mixed and fluctuating ORR selectivity, ranging from 11% (observed at ~0.33 V *vs.* RHE) to 68% (observed at ~0.62 V *vs.* RHE). CoN$_{2+x}$-ACNT tends to reduce O_2 to H_2O, demonstrating a < 10% $FE_{H_2O_2}$ when the applied potential is lower than 0.4 V *vs.* RHE.

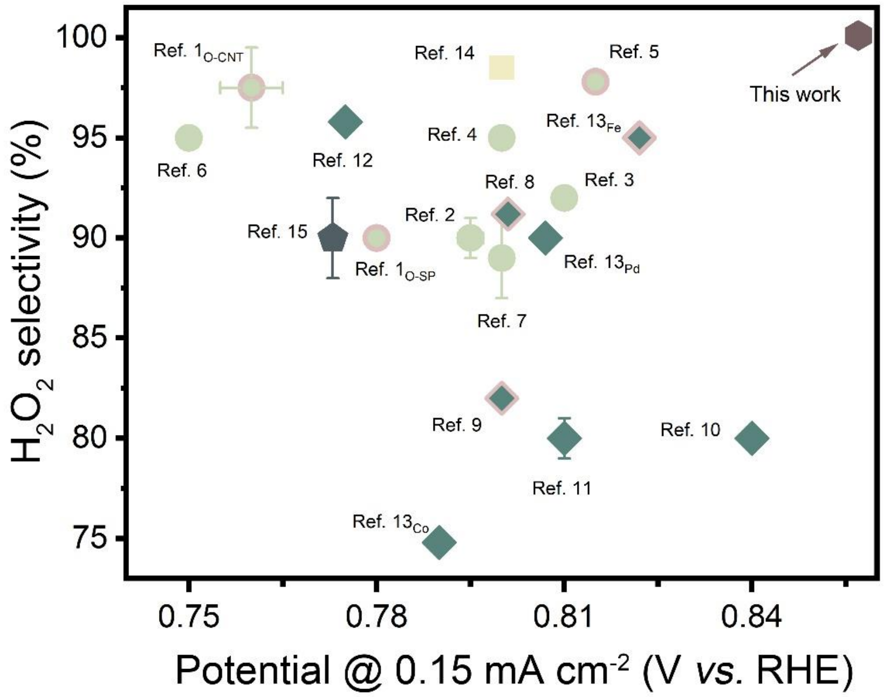

Supplementary Figure 12 Comparison of the onset potential and corresponding selectivity for H_2O_2 electrosynthesis on CoN_{4+4}-ACNT and recently-reported benchmark electrocatalysts (in alkaline medium). The CoN_{4+4}-ACNT shows way better onset potential (0.857 V *vs.* RHE) and selectivity (~100%) over previously reported works to our knowledge.

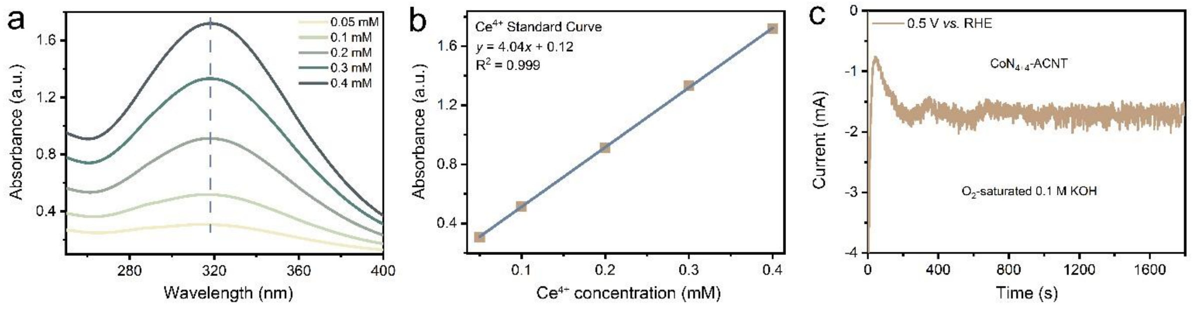

Supplementary Figure 13 Bulk H_2O_2 electrosynthesis. a UV-Vis spectra of a series of standard solutions with Ce^{4+} concentrations of 0.05 mM, 0.1 mM, 0.2 mM, 0.3 mM, and 0.4 mM, respectively. **b** Calibration curve used for calculation of Ce^{4+} concentrations (derived from Supplementary Figure 13a). **c** Chronoamperometry test of CoN_{4+4}-ACNT at 0.5 V *vs.* RHE for 30 min in 0.1 M KOH with a H-cell configuration. The H_2O_2 generation rate is calculated to be 2.2 mg cm^{-2} h^{-1} and the $FE_{H_2O_2}$ is 98%.

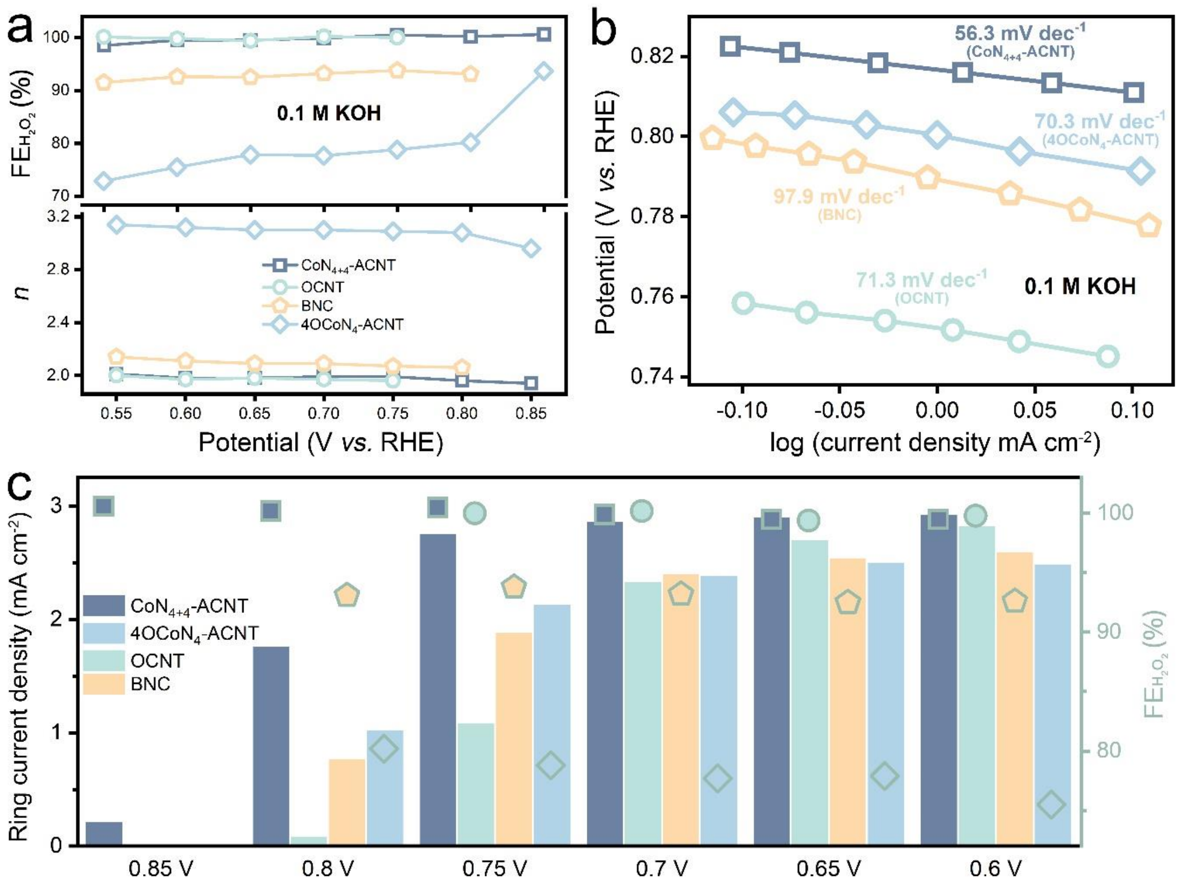

Supplementary Figure 14 Comparison of ORR performance of CoN4+4-ACNT, OCNT, BNC and 4OCoN4-ACNT. a The calculated $FE_{H_2O_2}$ and *n* (from Figure 3d in the main text) as a function of the applied potential. **b** Tafel plots of ring current density derived from Figure 3d in the main text. **c** Ring current density (columns) and corresponding $FE_{H_2O_2}$ (dots) in the potential range of 0.6-0.85 V *vs.* RHE.

As can be inferred from Figure 3e and Supplementary Figures 14a and c, oxygen-functionalized CNT (OCNT) shows high H_2O_2 selectivity ($FE_{H_2O_2}$ closes to 100% ranging from 0.55-0.79 V *vs.* RHE) but poor activity (E_{onset} = 0.79 V *vs.* RHE; $E_{@1\ mA\ cm^{-2}}$ = 0.75 V *vs.* RHE; $E_{@2\ mA\ cm^{-2}}$ = 0.72 V *vs.* RHE; $j_{@0.8\ V}$ = 0.084 mA cm^{-2}; $j_{@0.7\ V}$ = 2.33 mA cm^{-2}). Boron and nitrogen co-doped carbon (BNC) exhibits improved activity (E_{onset} = 0.84 V *vs.* RHE; $E_{@1\ mA\ cm^{-2}}$ = 0.79 V *vs.* RHE; $E_{@2\ mA\ cm^{-2}}$ = 0.74 V *vs.* RHE; $j_{@0.8\ V}$ = 0.767 mA cm^{-2}; $j_{@0.7\ V}$ = 2.4 mA cm^{-2}) but inferior selectivity ($FE_{H_2O_2}$ ~ 93% ranging from 0.55-0.8 V *vs.* RHE) compared to that of OCNT. HNO_3 treated CoN4-ACNT (4OCoN4-ACNT) displays even better activity (E_{onset} = 0.841 V *vs.* RHE; $E_{@1\ mA\ cm^{-2}}$ = 0.8 V *vs.* RHE; $E_{@2\ mA\ cm^{-2}}$ = 0.76 V *vs.* RHE; $j_{@0.8\ V}$ = 1.02 mA cm^{-2}; $j_{@0.7\ V}$ = 2.38 mA cm^{-2}) over that of BNC but its $FE_{H_2O_2}$ goes down below 80%. It can be concluded that a "selectivity-activity dilemma" exists in the current ORR 2e$^-$ catalysts design, leaving a vacancy for electrocatalysts with both high activity and selectivity for H_2O_2 synthesis from O_2 reduction.

The CoN4+4-ACNT shows not only the best activity (E_{onset} = 0.857 V *vs.* RHE; $E_{@1\ mA\ cm^{-2}}$ = 0.82 V *vs.* RHE; $E_{@2\ mA\ cm^{-2}}$ = 0.79 V *vs.* RHE; $j_{@0.8\ V}$ = 1.757 mA cm^{-2}; $j_{@0.7\ V}$ = 2.86 mA cm^{-2}) and 2e$^-$ ORR reaction kinetics (a low Tafel slope of 56.3 mV dec^{-1}, Supplementary Figure 14b) among all the four candidates, but also the highest selectivity ($FE_{H_2O_2}$ ~100% ranging

from 0.55-0.85 V *vs.* RHE). The superior performance of CoN_{4+4}-ACNT is considered a breakthrough of the 2e$^-$ ORR "selectivity-activity dilemma" that researchers have been exploring for in the past decades.

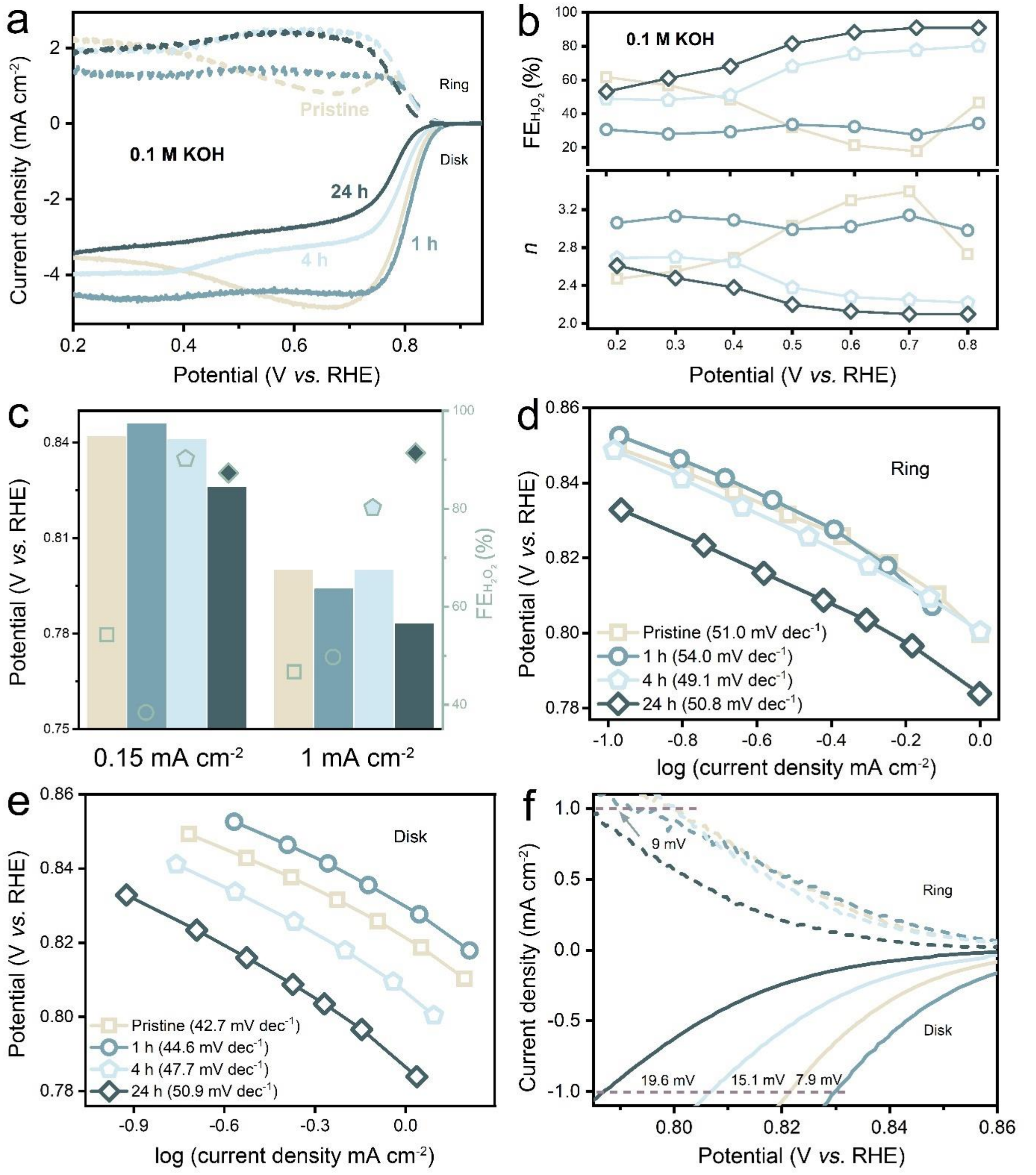

Supplementary Figure 15 Comparison of ORR performance of ACNTs that are composited with pristine CoN_4 or HNO_3 modified-CoN_4 molecules. a, b (**a**) Comparison of ORR performance of $OCoN_4$-ACNT series samples at 1600 rpm in O_2-saturated 0.1 M KOH and (**b**) the calculated $FE_{H_2O_2}$ and *n* as a function of the applied potential. **c** Potentials (columns)

and corresponding $FE_{H_2O_2}$ (dots) at ring current densities of 0.15, 1 and 2 mA cm^{-2}. **d, e** Tafel plots of (**d**) ring and (**e**) disk current densities derived from Supplementary Figure 15a. **f** Disk and ring current densities of the $OCoN_4$-ACNT series samples near onset potentials.

To make CoN_4 a $2e^-$-oriented ORR active centre, HNO_3 treatment was applied on the metal-nitrogen moieties for 1, 4 and 24 h, respectively. After hybridizing with ACNT, the resulted materials are labelled as *x*$OCoN_4$-ACNT (*x* represents the acid wash time in units of hours). MP-AES was employed to examine their metal loading (results shown in Supplementary Table 3). It is found CoN_4-ACNT, $1OCoN_4$-ACNT and $4OCoN_4$-ACNT show similar Co mass loading (indicating an almost identical active site population of the three samples). Whereas, $24OCoN_4$-ACNT exhibits a smaller Co content (indicates a reduced active site population of $24OCoN_4$-ACNT compared to CoN_4-ACNT). The different linear sweep voltammetry (LSV) shapes of $1OCoN_4$-ACNT and pristine CoN_4-ACNT (Supplementary Figure 15a) indicate changes in the Co-N coordination of the CoN_4 molecule after the oxidation treatment. Consequently, $1OCoN_4$-ACNT ($FE_{H_2O_2}$ ~30% throughout the entire scan) and CoN_4-ACNT ($FE_{H_2O_2}$ varies a lot with the applied potential, ranging from 16-63%) exhibit differing ORR selectivity (Supplementary Figure 15b). The $FE_{H_2O_2}$ witnessed a dramatic boost (from ~30% of $1OCoN_4$-ACNT to ~75% of $4OCoN_4$-ACNT) after a longer period of (4 h) HNO_3 treatment. Further acid wash (up to 24 h) can lift the $FE_{H_2O_2}$ to 90% but lead to sluggish (due to removal of Co active site as implied by the MP-AES measurement discussed above) reaction kinetics (E_{onset} = 0.841 V *vs.* RHE and $E_{@1\ mA\ cm^{-2}}$ = 0.8 V *vs.* RHE for $4OCoN_4$-ACNT but E_{onset} = 0.826 V *vs.* RHE and $E_{@1\ mA\ cm^{-2}}$ = 0.783 V *vs.* RHE for $24OCoN_4$-ACNT) as can be learned from Supplementary Figure 15c. Therefore, from the perspective of both activity and selectivity, $4OCoN_4$-ACNT is regarded as the most optimized sample among the $OCoN_4$-ACNT series catalysts for H_2O_2 electrosynthesis.

It is worth to note that no obvious enhancement can be found in the E_{onset} and $E_{@1\ mA\ cm^{-2}}$ of pristine CoN_4-ACNT and 1 or $4OCoN_4$-ACNT (Supplementary Figure 15c). Moreover, CoN_4-ACNT and 1 or $4OCoN_4$-ACNT share almost identical Tafel slope from the ring current density (Supplementary Figure 15d) but gradually decreased Tafel slope from the disk current density (Supplementary Figure 15e), implying the improved $FE_{H_2O_2}$ of the oxidized samples is mainly due to the weakened $4e^-$ reaction kinetics rather than advanced $2e^-$ activity. The enlarged polarization curves near the onset potential shown in Supplementary Figure 15f clearly demonstrate an underdeveloped ring current densities but shrank disk current densities of the $OCoN_4$-ACNT samples, which agrees with the Tafel plot results.

Based on the above results, there are three points to emphasize: (1) ORR selectivity can be manipulated by tailoring active centre configuration or its surrounding coordination. (2) Owing to the incomprehensive understanding of $2e^-$ ORR catalyst/process at an atomic level, catalysts made by the "active site/surrounding coordination tailoring strategy" tend to exhibit poor selectivity. Consequently, as stated in Figure 1b in the main text, the activity of the $OCoN_4$-ACNT samples is still nowhere near the thermodynamic limit due to the diverted "OOH^* stream" at the ORR secondary stage (desorption and dissociation of OOH^*) despite the well-optimized O_2 adsorption and OOH^* desorption collaboration. (3) Active site/surrounding coordination modification methods such as acid treatment, ammonia atmosphere annealing, *etc.* are intractable, thus leading to over-adjustment (*i.e.* $24OCoN_4$-ACNT).

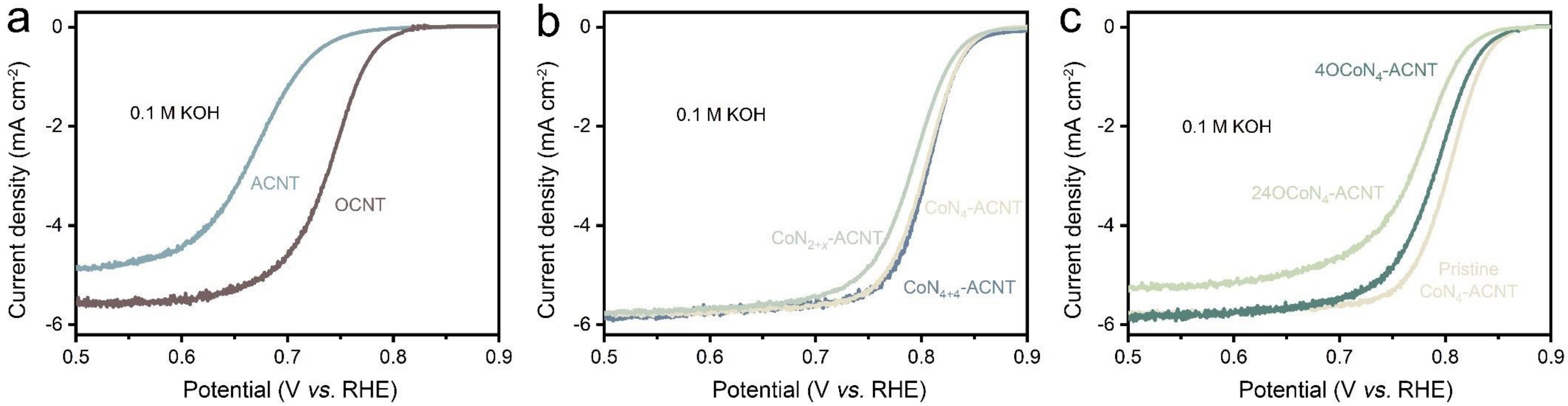

Supplementary Figure 16 ECD studies of **a** ACNT and OCNT, **b** CoN$_x$-ACNT series samples and **c** OCoN$_4$-ACNT series samples.

Many researchers compare the observed disk current density to the theoretical mass transfer limitation when evaluating 4e$^-$ ORR catalysts to check if the catalysts can deliver full-capacity of oxygen reduction performance. However, this comparison has seldom been made when testing 2e$^-$ ORR catalysts which might be attributed to the hybridized selectivity that leads to difficulty in setting a current density comparison standard. For further clarification, assuming catalyst A shows full 4e$^-$ ORR selectivity which converts x O_2 to H_2O and delivers a disk current density of -4x; catalyst B shows mixed ORR selectivity which converts 0.5x O_2 to H_2O, 0.5x O_2 to H_2O_2 and delivers a disk current density of -3x, accordingly a ring current density of x; catalyst C shows full 2e$^-$ ORR selectivity which converts x O_2 to H_2O_2 and delivers a disk current density of -2x, accordingly a ring current density of 2x; catalyst D shows full 4e$^-$ ORR selectivity but only converts 0.5x O_2 to H_2O and delivers a disk current density of -2x. Catalyst A, B and C reduced x O_2 during the ORR process (indicating all of the three catalysts exhibit exact the same oxygen reduction activity) but delivered different disk current densities (owing to the varied selectivity). Whereas, catalyst D was only able to reduce 0.5x O_2, revealing its inferior activity compared to the other three catalysts. Hence, the ORR activity of the four catalysts should be in the order of A = B = C > D. Whereas, the ORR activity order would be A > B > C = D if disk current density is the metric. On the above assumptions, one can tell that current density cannot always reflect the real intrinsic ORR activity.

Equivalent current density (ECD), a hypothetical ORR current density which is calculated assuming all the O_2 molecules that are reduced to H_2O_2 during ORR (in other words the O_2 molecules that are underwent the 2e$^-$ reaction pathway) will finally be converted to H_2O (ECD = $-|j_{disk}| - |j_{ring}|$), is proposed in this work to better measure the intrinsic ORR activity of the catalyst. For example, the ECD of catalyst A, B, C and D can be calculated as $ECD_A = -4x$, $ECD_B = -3x - x = -4x$, $ECD_C = -2x - 2x = -4x$ and $ECD_D = -2x$, respectively. The ECD trend ($ECD_A = ECD_B = ECD_C > ECD_D$) agrees with the intrinsic activity trend (A = B = C > D), confirming the rationality of ECD as an ORR activity metric.

The ECDs of OCNT/ACNT, CoN_x-ACNT and xOCoN_4-ACNT are calculated and shown in Supplementary Figure 16. The noticeable improvement in ECD of OCNT compared to that of ACNT (Supplementary Figure 16a) suggests the oxidation treatment generates fresh ORR active moieties onto the nanotube that correspondingly results in the enhanced activity of OCNT (Supplementary Figure 19a). Hence, the oxidation treatment of catalytically-inert carbons is regarded as a construction-driven modification method. The calculated ECDs of CoN_x-ACNT series samples are almost identical (Supplementary Figure 16b) which explains their similar onset ORR potentials (Figure 3a in the main text), indicating their varied disk current densities are due to differences in selectivity rather than activity. As for the xOCoN_4-ACNT samples, the ECDs of 4OCoN_4-ACNT shows no obvious change when compared with that of pristine CoN_4-ACNT (Supplementary Figure 16c). Interestingly, a significant drop in ECD of 24OCoN_4-ACNT can be witnessed (light green line in Supplementary Figure 16c) which indicates the strong HNO_3 wash not only modifies, but also partially eliminates the active sites of the CoN_4 molecule. In other words, the weaker activity of 24OCoN_4-ACNT can be ascribed to over-modification (that is to say active site/surrounding coordination tailoring tools such as acid treatment can be a destruction-driven technique if overruns).

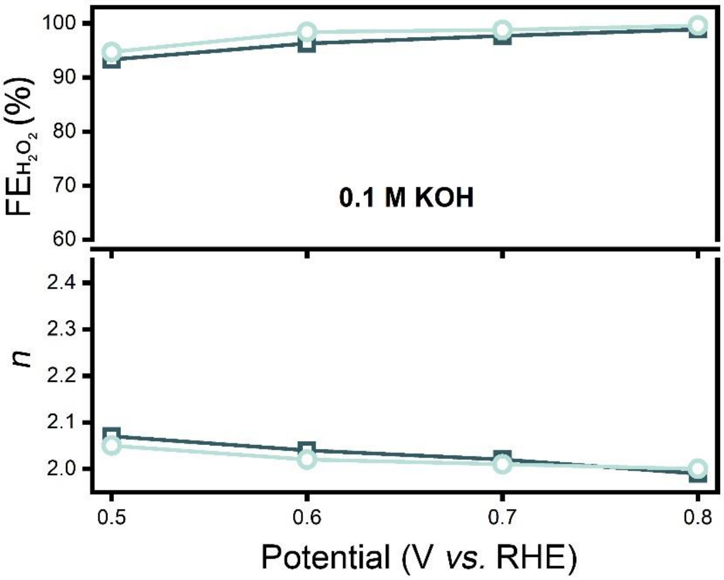

Supplementary Figure 17 The calculated $FE_{H_2O_2}$ and n (from Figure 3f in the manuscript) as a function of the applied potential of CoN_{4+4}-ACNT before (dark green lines) and after (cyan blue lines) 10,000 cycles of cyclic voltammetry (CV) operation (between -0.8-0 V *vs.* Ag/AgCl) in O_2-saturated 0.1 M KOH.

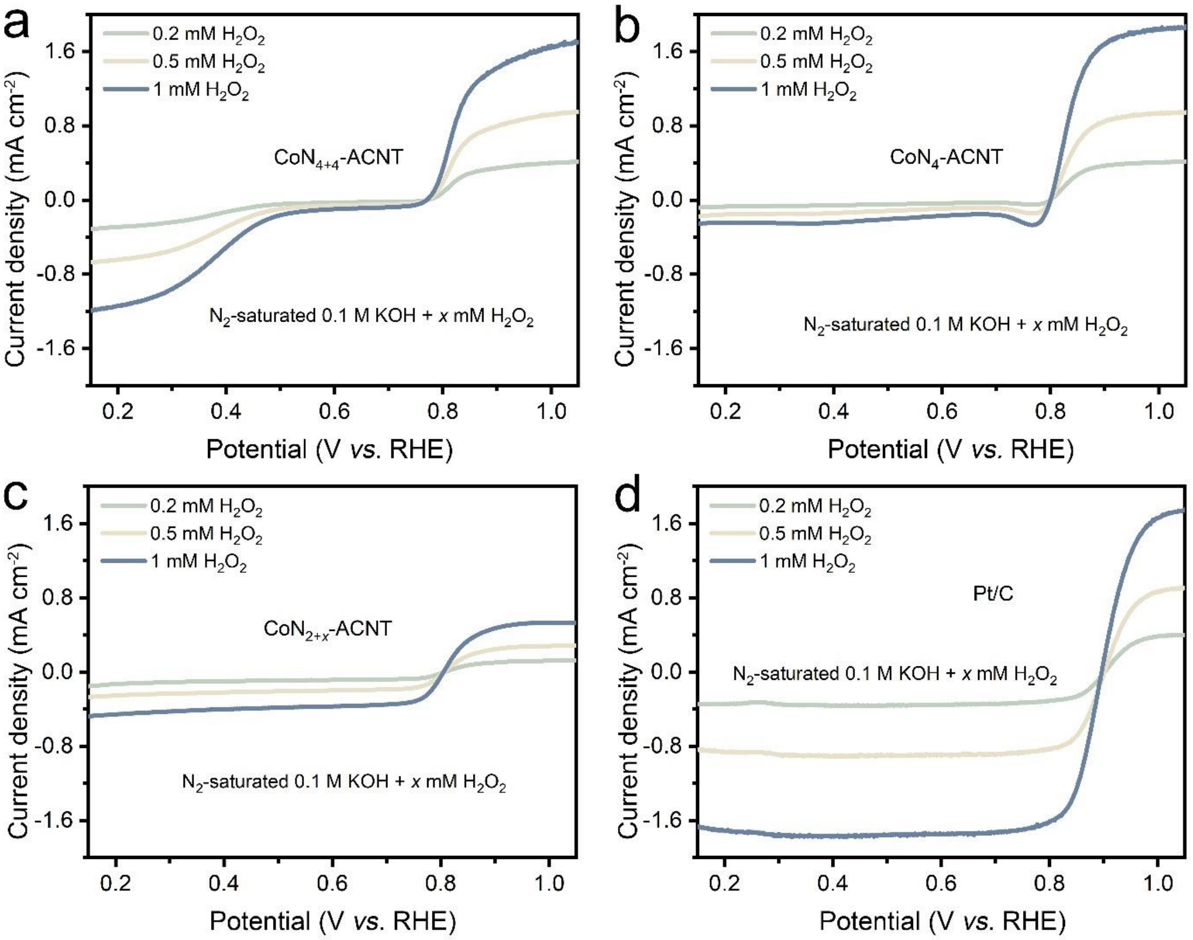

Supplementary Figure 18 H_2O_2 reduction reaction (H_2O_2RR) studies of **a** CoN_{4+4}-ACNT, **b** CoN_4-ACNT, **c** CoN_{2+x}-ACNT and **d** commercial 20 *wt.*% Pt/C in N_2-saturated 0.1 M KOH with various concentrations of H_2O_2 at 1600 rpm.

On one hand, it would be ideal for a 4e$^-$ ORR catalyst to be capable of reducing H_2O_2 to H_2O. Whereas, the H_2O_2RR may also terminate at midway, generating OH·, which may consequently damage the catalyst and "mute" its activity.[29] On the other hand, electroreduction of H_2O_2 by a 2e$^-$ ORR catalyst is not desirable for efficient production of H_2O_2.

H_2O_2RR tests were performed to check the catalytical behaviour of CoN_x-ACNT samples towards the 2e$^-$ ORR product. As demonstrated in Supplementary Figure 18, H_2O_2 oxidation/reduction current density plateau can be observed in the LSVs of all the four catalysts, suggesting H_2O_2 oxidation/reduction reaction might be a mass transfer limited process. It is noteworthy that the H_2O_2RR current density of Pt/C (Supplementary Figure 18d) is proportional to the H_2O_2 concentration (*i.e.* at 0.6 V *vs.* RHE, j = 0.35, 0.89 and 1.75 mA cm^{-2} when the H_2O_2 concentration is 0.2, 0.5 and 1 mM, respectively), double confirming that H_2O_2RR is mass-transfer limited. Assuming Pt/C can reduce H_2O_2 all the way to H_2O, its H_2O_2RR behaviour can be used as a metric. CoN_{4+4}-ACNT exhibits no H_2O_2RR activity at potentials higher than ~0.5 V *vs.* RHE (Supplementary Figure 18a). The almost negligible H_2O_2RR current agrees with its ~100% 2e$^-$ ORR selectivity. Similarly, as can be learned from Supplementary Figure 18b, the H_2O_2RR current density of CoN_4-ACNT (at 0.6 V *vs.* RHE, j = 0.04, 0.09 and 0.17 mA cm^{-2} when the H_2O_2 concentration is 0.2, 0.5 and 1 mM, respectively) is way smaller than that of Pt/C (which is assumed as the theoretical mass-transfer limitation), indicating CoN_4-ACNT can be regarded as H_2O_2RR-inactive. As for CoN_{2+x}-ACNT, its low but traceable H_2O_2RR current density (at 0.6 V *vs.* RHE, j = 0.09, 0.20 and 0.37 mA cm^{-2} when

the H_2O_2 concentration is 0.2, 0.5 and 1 mM, respectively) reveals it can catalyse the reduction of H_2O_2.

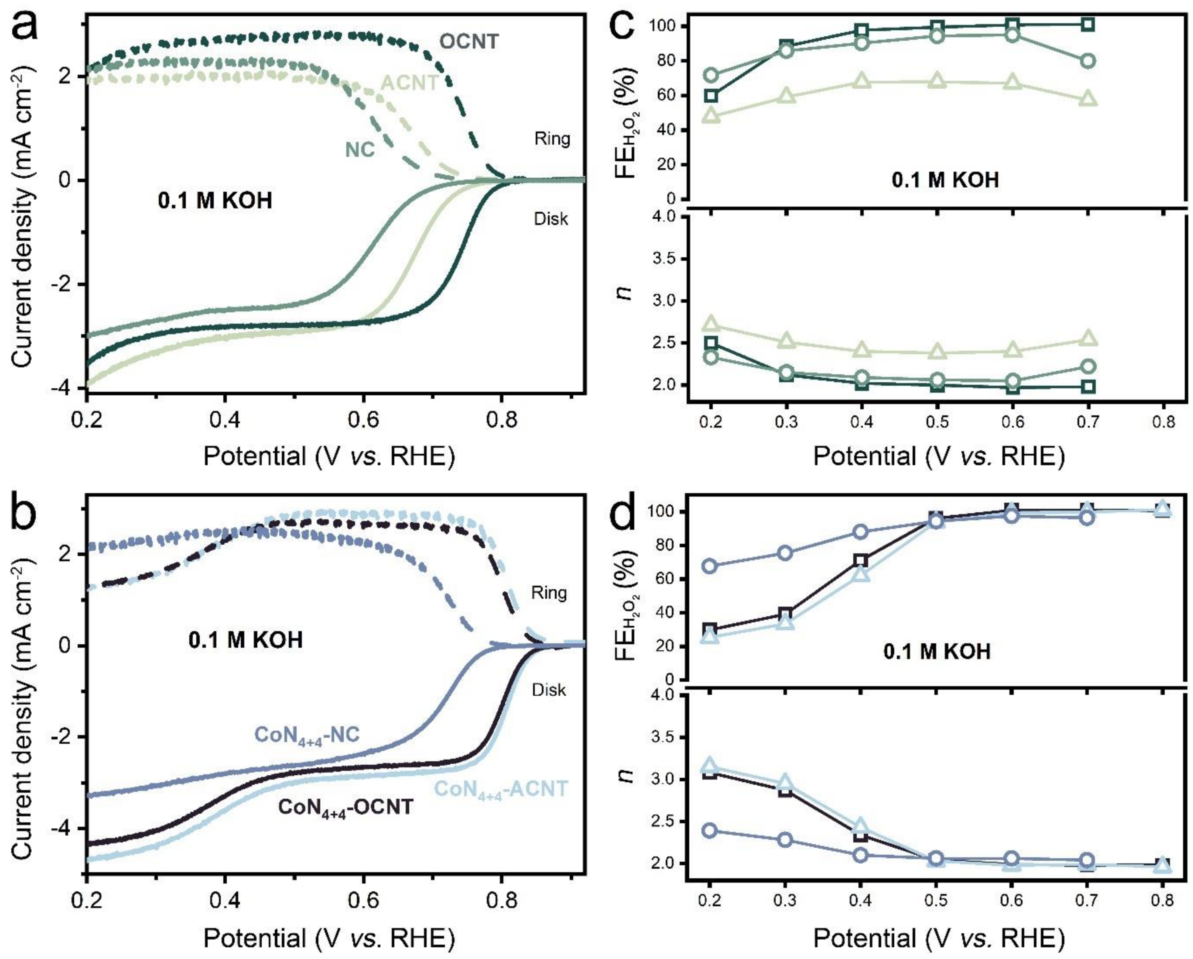

Supplementary Figure 19 a, b (**a**) Comparison of ORR performance of ACNT, OCNT and NC at 1600 rpm in O_2-saturated 0.1 M KOH and (**b**) the calculated $FE_{H_2O_2}$ and *n* as a function of the applied potential. **c, d** (**c**) Comparison of ORR performance of CoN_{4+4}-ACNT, CoN_{4+4}-OCNT and CoN_{4+4}-NC at 1600 rpm in O_2-saturated 0.1 M KOH and (**d**) the calculated $FE_{H_2O_2}$ and *n* as a function of the applied potential.

The varied selectivity of CoN_x-ACNTs was first attributed to the different interaction between the O-groups of the carbon supports and the CoN_x moieties. To clarify the role of the oxygen-containing ACNT substrates, we first consider the CoN_{4+4}-ACNT that shows the best $2e^-$ ORR performance. The ORR performances of three different carbon materials with or without CoN_{4+4} decoration were examined. The ORR performance of ACNT, OCNT and commercial nano-carbon (NC) was evaluated in alkaline medium. As can be found in Supplementary Figures 17a and b, the bare carbon samples exhibit differing ORR activity and selectivity which can be attributed to their varied O-content (O composition around 0.6, 6.39 and 0.91 wt. % in ACNT, OCNT and NC, respectively based on XPS analysis) according to the previous literature. On the contrary, the carbons that are composited with CoN_{4+4} show nearly identical

ORR selectivity ($FE_{H_2O_2}$ ~100% in high-potential region ranging from ~0.50-0.85 V *vs.* RHE), suggesting the ultra-high H_2O_2 production selectivity of the CoN_{4+4}/carbon hybrids comes from the CoN_{4+4} active sites and is independent from the carbon supports (Supplementary Figures 19c and d). It is noteworthy that the carbon substrate may influence the activity of the composites (*i.e.* the inferior activity of CoN_{4+4}-NC compared to that of CoN_{4+4}-ACNT and CoN_{4+4}-OCNT) which can possibly be ascribed to the varied active site-carbon framework interaction that alters the electrical conductivity, specific surface area, active site density, *etc.* of the composites.

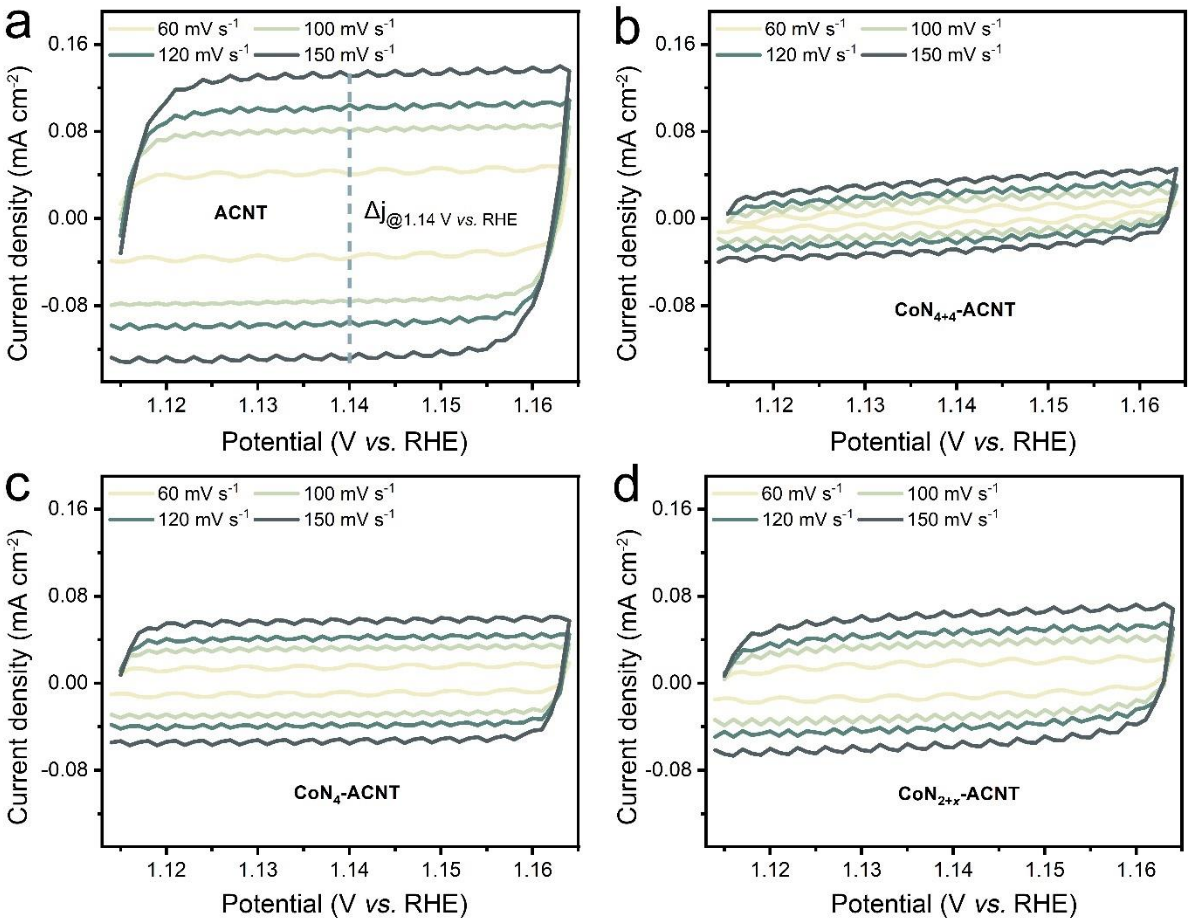

Supplementary Figure 20 Cyclic voltammetry (CV) scanning curves of **a** ACNT, **b** CoN_{4+4}-ACNT, **c** CoN_4-ACNT and **d** CoN_{2+x}-ACNT catalysts in 0.1 M KOH solution at different scan rates in the non-Faradaic potential region (1.115-1.165 V *vs.* RHE).

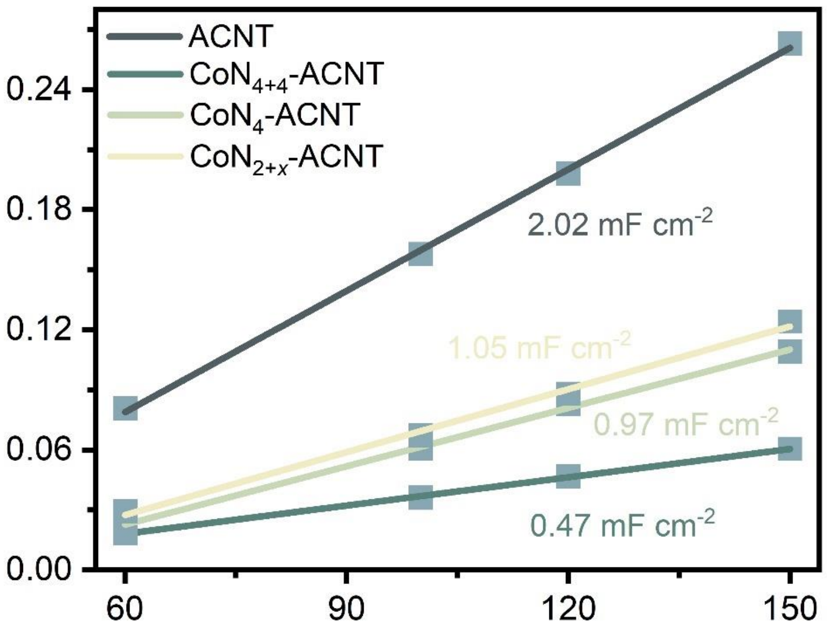

Supplementary Figure 21 Capacitive current density differences at 1.14 V *vs.* RHE as a function of scan rate (data obtained from Supplementary Figure 20). The linear slope is equivalent to the electrochemical active surface area (ECSA) of the electrodes. As can be inferred from Supplementary Figures 20 and 21, the ECSAs of all three metal/carbon composites decreased compared to bare ACNT owing to the poor electrical conductivity of the CoN_x molecules. It can be concluded that the main function of the ACNT substrate is to provide mechanical and electron-conductive support for the semi-conductive CoN_x active sites.

Supplementary Table 3 Mass loading of Co measured by MP-AES

**Sample**	CoN_{4+4}-ACNT (0.25-1)	CoN_{4+4}-ACNT (0.5-1)	CoN_{4+4}-ACNT (1-1)	CoN_{4+4}-ACNT (2-1)	CoN_{4+4}-ACNT (4-1)	CoN_4-ACNT	1OCoN_4-ACNT	4OCoN_4-ACNT	24OCoN_4-ACNT	CoN_{2+x}-ACNT
**Co mass loading (wt%)**	0.009	0.018	0.042	0.105	0.165	0.122	0.116	0.142	0.083	0.011

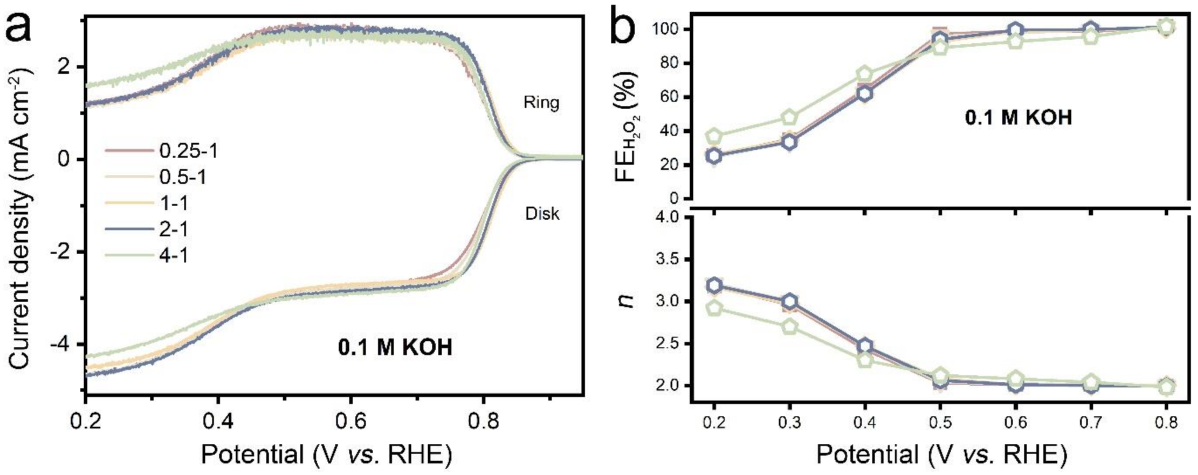

Supplementary Figure 22 a, b (**a**) Comparison of ORR performance of CoN_{4+4}-ACNT prepared with different metal/carbon precursor mass ratios (*i.e.* 0.25-1 represents the CoPc/ACNT mass ratio is 1/4) at 1600 rpm in O_2-saturated 0.1 M KOH and (**b**) the calculated $FE_{H_2O_2}$ and *n* as a function of the applied potential. A series of CoN_{4+4}-ACNT catalysts with different metal loadings (measured by MP-AES, Supplementary Table 3) were synthesised. Slightly better ORR onset and half-wave potentials can be witnessed when the metal precursor to carbon substrate ratio increases from 1:4 to 2:1 (Supplementary Figure 22a). No obvious ORR selectivity (Supplementary Figure 22b) alteration can be observed, indicating the impact of metal loading on ORR selectivity of CoN_x-ACNT samples (measured by RRDE system at 1600 rpm rotating rate) is almost negligible. Such discovery is in accordance with previous literature in which the catalysts are prepared by a metal active site immobilisation method similar to this work.[30] A noticeable activity and selectivity variation can be observed with further increases of the CoN_{4+4}/ACNT ratio to 4:1. This can possibly be attributed to aggregation of Co-N moieties (as a result, some of the Co would not remain in single atom state). To avoid the influence of metal loading on selectivity and to allow the catalyst to deliver the best activity, we set the metal precursor to carbon support ratio as 2:1 in this work.

Supplementary Table 4 ORR performance of ACNT in 0.1 M KOH.

**Potential (V *vs.* RHE)**	**$FE_{H_2O_2}$ (%)**	**H_2O_2 selectivity (%)**	***n***
0.7	57.4	73	2.54
0.6	67	80	2.4
0.5	67.9	80.8	2.38
0.4	67.8	80.7	2.4
0.3	59.1	74.3	2.51
0.2	47.7	64.6	2.71

Supplementary Table 5 ORR performance of OCNT in 0.1 M KOH.

**Potential (V *vs.* RHE)**	**$FE_{H_2O_2}$ (%)**	**H_2O_2 selectivity (%)**	***n***
0.7	101.1	101	1.98
0.6	100.8	100.6	1.97
0.5	99.5	99.8	2
0.4	97.7	98.8	2.02
0.3	88.4	93.9	2.12
0.2	59.8	74.8	2.5

Supplementary Table 6 ORR performance of NC in 0.1 M KOH.

**Potential (V *vs.* RHE)**	**$FE_{H_2O_2}$ (%)**	**H_2O_2 selectivity (%)**	***n***
0.7	80	88.9	2.22
0.6	95	98	2.05
0.5	94.4	97.1	2.06
0.4	90.2	94.8	2.09
0.3	85.8	92.4	2.15
0.2	71.7	83.5	2.33

Supplementary Table 7 ORR performance of CoN_{4+4}-ACNT in 0.1 M KOH.

**Potential (V *vs.* RHE)**	**$FE_{H_2O_2}$ (%)**	**H_2O_2 selectivity (%)**	***n***
0.8	101.2	101	1.96
0.7	99.9	100	1.99
0.6	99.5	99.7	1.98
0.5	93.9	96.8	2.03
0.4	62.1	76.6	2.43
0.3	33.5	50.2	2.95
0.2	25.4	40.5	3.15

Supplementary Table 8 ORR performance of CoN_{4+4}-OCNT in 0.1 M KOH.

**Potential (V *vs.* RHE)**	**$FE_{H_2O_2}$ (%)**	**H_2O_2 selectivity (%)**	***n***
0.8	100.7	100.4	1.98
0.7	101	100.5	1.98
0.6	100.9	100.5	1.99
0.5	96.1	98	2.04
0.4	71.1	83.1	2.34
0.3	39.2	56.4	2.87
0.2	29.9	46.1	3.08

Supplementary Table 9 ORR performance of CoN_{4+4}-NC in 0.1 M KOH.

**Potential (V *vs.* RHE)**	**$FE_{H_2O_2}$ (%)**	**H_2O_2 selectivity (%)**	***n***
0.7	96.4	98.2	2.04
0.6	97.5	98.7	2.06
0.5	94.4	97.1	2.06
0.4	88.1	93.7	2.1
0.3	75.5	86.1	2.28
0.2	67.7	80.7	2.39

Supplementary Table 10 ORR performance of CoN_4-ACNT in 0.1 M KOH.

**Potential (V *vs.* RHE)**	**$FE_{H_2O_2}$ (%)**	**H_2O_2 selectivity (%)**	***n***
0.8	46.7	63.7	2.73
0.7	17.8	30.3	3.39
0.6	21.3	35.1	3.3
0.5	32.4	48.9	3.02
0.4	48.5	65.3	2.69
0.3	56.9	72.6	2.55
0.2	61.7	76.3	2.47

Supplementary Table 11 ORR performance of CoN_{2+x}-ACNT in 0.1 M KOH.

Potential (V *vs.* RHE)	$FE_{H_2O_2}$ (%)	H_2O_2 selectivity (%)	*n*
0.8	6.8	12.8	3.74
0.7	7.9	14.6	3.71
0.6	7.8	14.5	3.71
0.5	8.2	15.2	3.7
0.4	8.0	14.9	3.7
0.3	7.9	14.6	3.71
0.2	8.1	15	3.7

Supplementary Table 12 ORR performance of BNC in 0.1 M KOH.

Potential (V *vs.* RHE)	$FE_{H_2O_2}$ (%)	H_2O_2 selectivity (%)	*n*
0.8	93.1	96.4	2.06
0.7	93.2	96.5	2.09
0.6	92.6	96.1	2.11
0.5	90.1	94.8	2.16
0.4	88.3	93.8	2.27
0.3	81.2	89.6	2.42
0.2	71.9	83.7	2.68

Supplementary Table 13 ORR performance of $OCoN_4$-ACNT in 0.1 M KOH.

Potential (V *vs.* RHE)	$FE_{H_2O_2}$ (%)	H_2O_2 selectivity (%)	*n*
0.8	80.2	89	2.22
0.7	77.7	87.4	2.25
0.6	75.5	86	2.28
0.5	68	80.9	2.38
0.4	51	67.6	2.65
0.3	48.1	65	2.7
0.2	48.8	65.6	2.69

Supplementary Table 14 ORR performance of CoN_{4+4}-ACNT in 0.1 M PBS (pH = 7.4).

**Potential (V *vs.* RHE)**	**$FE_{H_2O_2}$ (%)**	**H_2O_2 selectivity (%)**	***n***
0.6	95.9	97.9	2.04
0.5	97.9	99	2.02
0.4	95.3	97.1	2.06
0.3	90.2	94.2	2.12
0.2	57	72.4	2.55
0.1	17.7	30.1	3.4

Supplementary Table 15 ORR performance of CoN_4-ACNT in 0.1 M PBS (pH = 7.4).

**Potential (V *vs.* RHE)**	**$FE_{H_2O_2}$ (%)**	**H_2O_2 selectivity (%)**	***n***
0.6	65.8	79.4	2.41
0.5	46.1	63.1	2.74
0.4	17.7	30	3.4
0.3	11.2	20.1	3.6
0.2	17.2	29.3	3.41
0.1	25	40	3.2

Supplementary Table 16 ORR performance of CoN_{2+x}-ACNT in 0.1 M PBS (pH = 7.4).

**Potential (V *vs.* RHE)**	**$FE_{H_2O_2}$ (%)**	**H_2O_2 selectivity (%)**	***n***
0.6	40	57.2	2.85
0.5	24.6	39.5	3.21
0.4	6.2	11.7	3.77
0.3	5.3	10	3.8
0.2	5.2	9.9	3.8
0.1	5.6	10.6	3.79

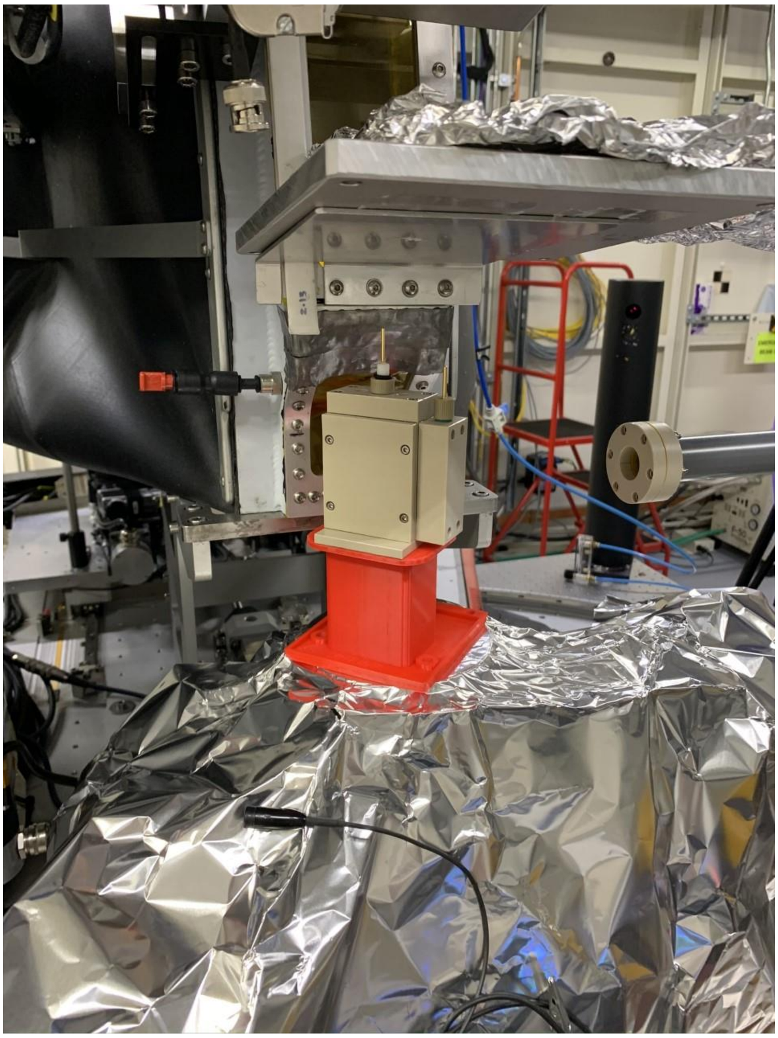

Supplementary Figure 23 Design of the electrochemical cell for *operando* HERFD-XANES measurement.[31]

Supplementary Figure 23 shows the *operando* electrochemical cell operating in fluorescence mode. The cell was equipped with a three-electrode system. The catalyst was loaded on the carbon cloth as the working electrode and sealed at hole on the wall by a Kapton foil. The cell was filled with electrolyte solution (0.1 M KOH), and the Pt counter electrode and saturated Ag/AgCl reference electrode were used. The cell was placed 45 degrees to incident X-ray beam direction. The K_β X-ray emissions were collected using three Ge(444) spherical analysers operated in Johann configuration with a 1 meter diameter Rowland circle.

Synthetic air was continuously purged into the electrolyte before and during the *operando* measurement. It is noteworthy that pure O_2 was not used due to special safety regulation from Diamond Light Source during COVID period. The authors acknowledge pure O_2 could potentially allow the catalysts to deliver better electrochemical response, accordingly leading to more pronounced alterations in HERFD-XANES signals. The working electrode was held at open circuit potential (OCP), 0.61 V and 0.26 V *vs.* RHE during each HERFD-XANES scan.

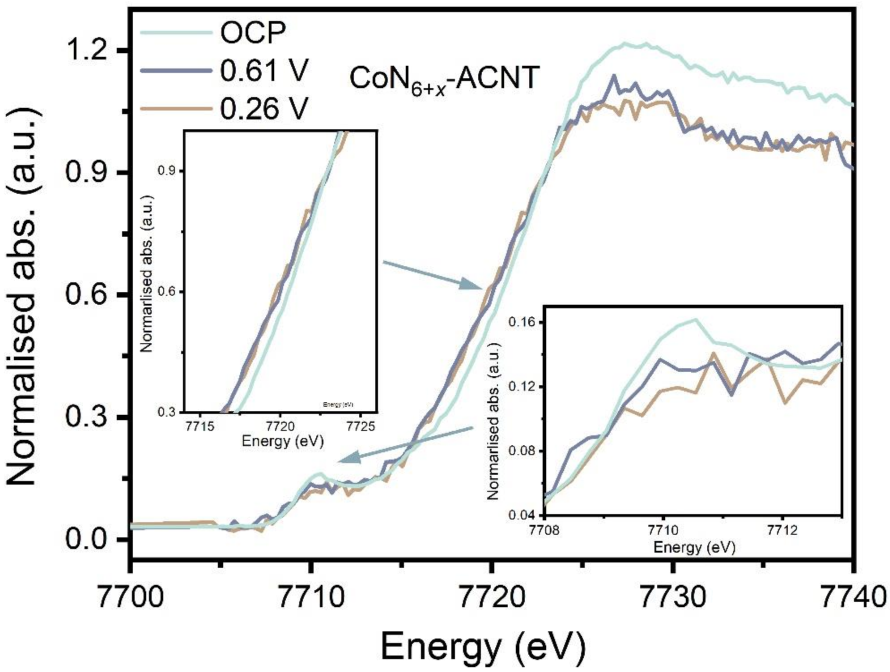

Supplementary Figure 24 *Operando* HERFD-XANES Co K-edge spectra of CoN_{2+x}-ACNT acquired during chronoamperometry test at OCP, 0.61 and 0.26 V *vs.* RHE. The shrank first transition and shifted edge position with the decreased cathodic bias point to the reduced valence state of the Co in CoN_{2+x}-ACNT. It is noteworthy that the alterations in the HERFD-XANES Co K-edge spectra are relatively limited which can be explained by the following two reasons. Firstly, only a small amount of the catalysts are able to establishing contact with the electrolyte. Secondly but the most likely, part of the negative charge is accepted by the polypyrrole chain electron acceptor system. Further experimental and computational studies are demanded to validate the hypothesis.

Supplementary Note 2

The HERFD measurement is much more advanced and totally different from the conventional total fluorescence yield (TFY) XANES or the partial fluorescence yield (PFY) mode XANES which are usually acquired by solid state detector. In conventional TFY/PFY-XANES the different fluorescence lines are not resolved; thus the energy resolution of the final spectra is limited by the Co 1s core-hole lifetime broadening. In comparison, the HERFD-XANES spectra in our experiment were collected by monitoring the intensity of Co Kβ1,3 mainline as a function of incident beam energy, which corresponds to the 3p core-hole final states with much less lifetime broadening. Therefore, the final spectra have much higher energy resolution, and could provide more resolved spectroscopic features that are usually hidden/overlapped in the conventional TFY/PFY-XANE. Such high energy resolution is extremely essential in our experiment, as the subtle changes of the electronic structure could only be resolved with HERFD under the reaction conditions. Also, considering with hard X-rays at Co K-edge that penetrate the whole electrode, all different Co intermediates formed though dynamic equilibrium will contribute to the final spectra under reaction conditions, which means the difference between the active and silent Co intermediates will be averaged, consequently leads

to smaller changes in the spectroscopy data.

The price for the higher energy resolution to resolve the subtle changes is the difficulties in the measurement. Since Co $K_{\beta1,3}$ has intensity of only approx. 1/15 of the Co Kα lines, and only the maximum intensity at the Co $K_{\beta1,3}$ peak is measured instead of full integration of the Co Kα regions, the signal intensity of final HERFD-XANES is more than one order of magnitude weaker than that of the conventional TFY/PFY-XANES. In addition, benefiting from the 4-bounce Si(111) scanning monochromator at I20-scanning beamline in Diamond Light Source, the energy resolution of the incident beam is also higher than the other spectroscopic beamlines which have the Si(111) double crystal monochromator. This ensured that the energy resolution of the final HERFD-XANES spectra were not limited by the broadening of the incident beam energy but reduced the total photon flux significantly, which resulted in less signal intensity. In brief summary, the relatively poor signal quality of HERFD-XANES is because what usually reported in the published papers are the results from conventional TFY/PFY-XANES. Instead, the more advanced HERFD-XANES is still not popularised due to the technical challenges and more strict requirements of samples, as well as less numbers of beamlines that can perform such measurement. But we believe HERFD-XANES is essential to our work as the changes in the 1s to 3d and 1s to $4p_z$ peaks of our catalysts can only be observed should the *operando* measurements be performed in HERFD mode.

In this work, we do not have the k-space spectra of the HERFD-XANES data as we only measured till 250 eV after the absorption edge (converted to k_{max} = 8). This k-range is not long enough to do EXAFS analysis but sufficient for background subtraction and normalisation of XANES spectra. For a reference, the *ex-situ* XAFS data were acquired till 900eV after the edge (converted to k_{max} = 15). In the EXAFS region, the oscillations after the absorption edge are caused by the back scattering of the photoelectrons and are not relevant to the Co 1s core-hole lifetime broadening at all. We did not measure the full *operando* EXAFS under HERFD mode because it would not give any additional information but would suffer significantly of the poor signal to noise ratio especially at high k region. Meanwhile, due to the mechanism of HERFD-XANES experiment that requires stepwise measurement of the Co $K_{\beta1,3}$ intensity with enough acquisition time (dwell time) at each incident energy point, such measurement will take more than 5 hours if long k-range EXAFS region is included. Typically the EXAFS would not be measured using HERFD mode, and to acquire the full EXAFS it has to be completely different beamline configuration than the HERFD experiment.

Supplementary Note 3

The exact accumulated intermediates can not be identified by XAS measurement due to the following reasons. First of all, as explained in Supplementary Note 2, measuring EXAFS under HERFD mode was unrealistic. Second, oxygen coordination in the first shell is difficult to be distinguished from the other four nitrogen atoms since both O and N have very similar back-scattering intensity in EXAFS (meanwhile, the coordination number of first shell N/O calculated from the fitting of EXAFS spectra also have an uncertainty/fitting error of 10-20%). Third, the Co-O distances in the intermediate species varies between 1.73A-1.91A according to the DFT calculation, which means even if the Co-O coordination could be extracted from the EXAFS fitting, the Debye-Waller factor of this Co-O scattering path would be very big and the coordination number would have a huge fitting error. Fourth, as EXAFS measured the average coordination environment of all Co species and not all Co species were in the form of having oxygen species adsorbed, the final EXAFS spectra would not be able to reveal the actual coordination structure of the active Co species during reaction. In fact, identifying ORR reaction intermediates by *operando* characterizations still remain challenging.[32] As O_2^* hydrogenation is normally regarded as the most thermodynamically unfavourable ORR step (for both $2e^-$ and $4e^-$ ORR as reported by literatures[11-12]), we believe the accumulated intermediate observed in our *operando* HERFD-XANES measurement is most likely to be O_2^*.

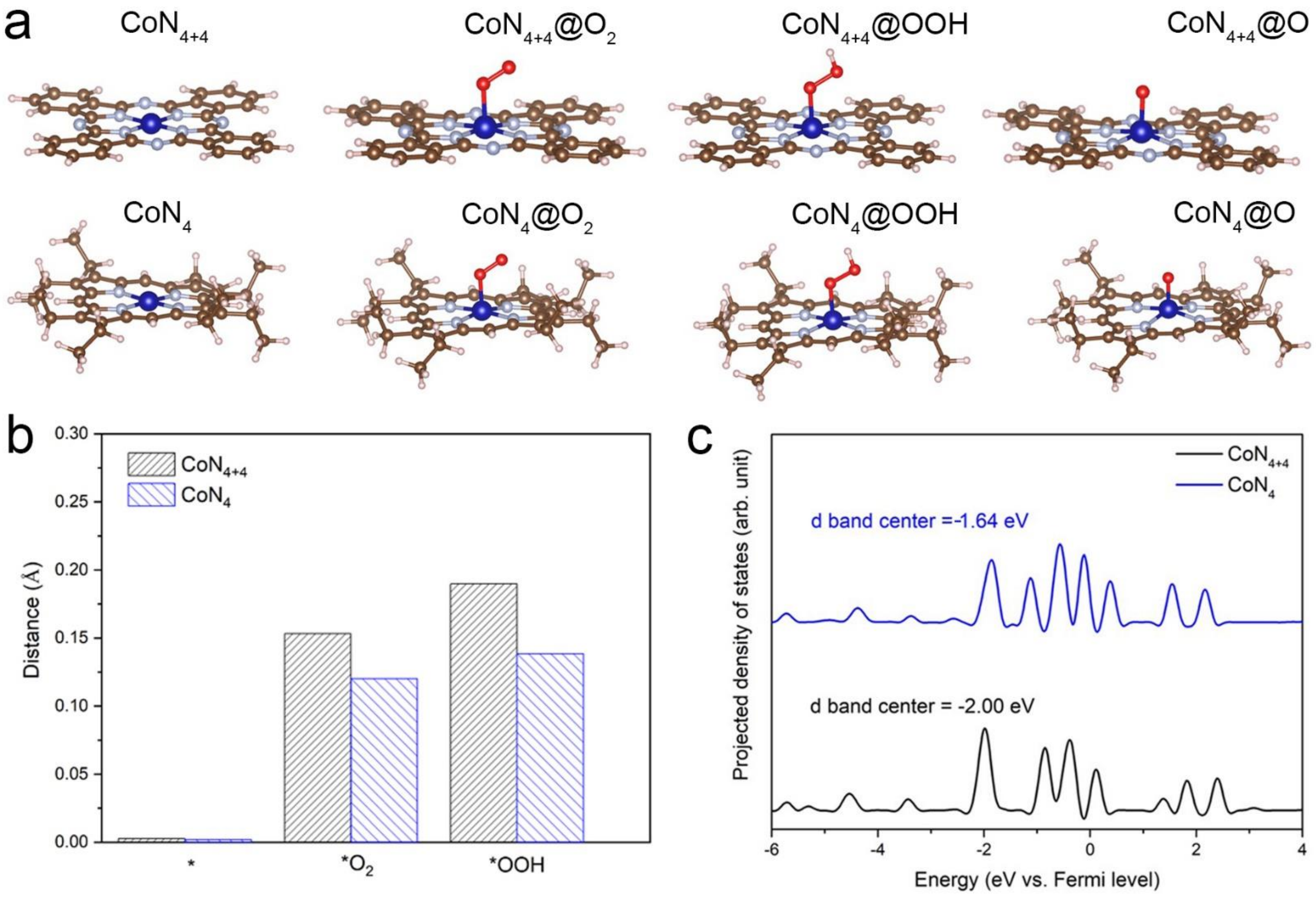

Supplementary Figure 25 a DFT-optimized ORR intermediate adsorption structures of CoN_{4+4} and CoN_4 models. **b** Simulated Co displacement (refers to the distance between the central Co atom and the plane consisting of the coordinated N atoms) with different adsorbed intermediates. **c** Projected density of states (PDOS) of Co 3d orbital of CoN_{4+4} and CoN_4 models.

To validate the hypothesis proposed in Figure 1 as well as to correlate the electrochemical (Figure 3) and the *operando* HERFD-XANES (Figure 4) results, DFT calculations were performed. The simulated adsorption structures of various ORR intermediates on both the CoN_{4+4} and CoN_4 active sites are displayed in Supplementary Figure 25a. It is found that, for deserted CoN_{4+4} and $CoN_{4,}$ the central Co atom and its coordinated N atoms are in the same plane. However, significant Co displacements can be witnessed (on both models) when adsorbed by intermediates. Such discovery is consistent with the *operando* HERFD-XANES measurement (Figure 4), that is, an applied potential can change the coordination environment of Co from square planar structure to a nonplanar penta-coordinated structure. Supplementary Figure 25b indicates that CoN_{4+4} possesses a higher Co displacement than CoN_4 (when adsorbed by O_2^* and OOH^*, which are the two intermediates associated with $2e^-$-ORR). Accordingly, at 0.61 V, a more pronounced decrease in the HERFD-XANES 1s to $4p_z$ peak of CoN_{4+4}-ACNT can be observed.

The projected density of states (PDOS) of CoN_{4+4} and CoN_4 were also calculated. As shown in Supplementary Figure 25c, CoN_{4+4} possesses a more negative d band center than CoN_4. A more negative d band center implies a weaker adsorption.

Supplementary Note 4

In this simulation, cobalt(II) phthalocyanine and cobalt(II) porphine shown in Supplementary Figure 26 are used as models for CoN_{4+4} and CoN_4 moieties, respectively. The structures of the cobalt(II) phthalocyanine and cobalt(II) porphine complexes and the accompanying reactants were optimised using density functional theory (DFT) implemented in the CRYSTAL17 code (please see the methodology section in the manuscript for more description). The electronic structure has been calculated for the molecules isolated and supported on different graphene substrates: perfect, N-doped and singly oxidised graphene oxide.

We have considered the electronic configuration of the active Co centres and its variation upon reduction of the molecule, as required upon application of a negative bias. Results are reported in Supplementary Table 17. In the reduced CoN_{4+4} moiety, however, the additional electron is hosted not on Co but on the porphyrin ring, with large contribution from the second shell neighbours. The corresponding spin density is illustrated in Figure 5b of the manuscript. This configuration is 0.200 eV more stable than the one where the additional electron is localised on Co, yielding a solution with no unpaired electrons.

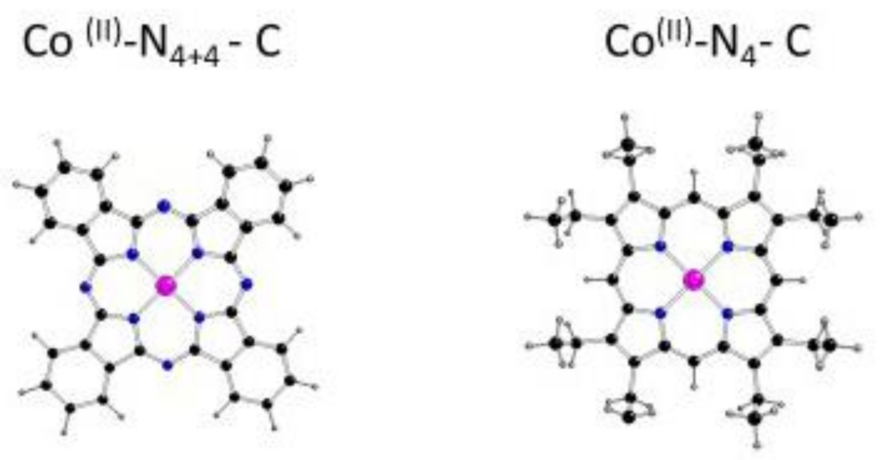

Supplementary Figure 26 Schematic illustration of the CoN_{4+4} and CoN_4 complexes. The pink, blue, black and grey spheres represent cobalt, nitrogen, carbon and hydrogen atoms.

Supplementary Table 17 Mulliken spin charge on Co for the high and low spin configuration of the CoN_4 and CoN_{4+4} complexes, and relative energy of the two electronic states.

	Total atomic spin on Co ($n_\alpha - n_\beta$)		ΔE/eV = $E_{high\ spin} - E_{low\ spin}$
	**Low spin**	**High spin**	
**$Co^{(II)}$-N_{4+4}-C**	1.035	2.690	+1.049
**$Co^{(II)}$-N_4-C**	1.012	2.696	+0.894
**$Co^{(I)}$-N_{4+4}-C**	0.000	1.063	-0.200
**$Co^{(I)}$-N_4-C**	0.000	2.295	+0.068

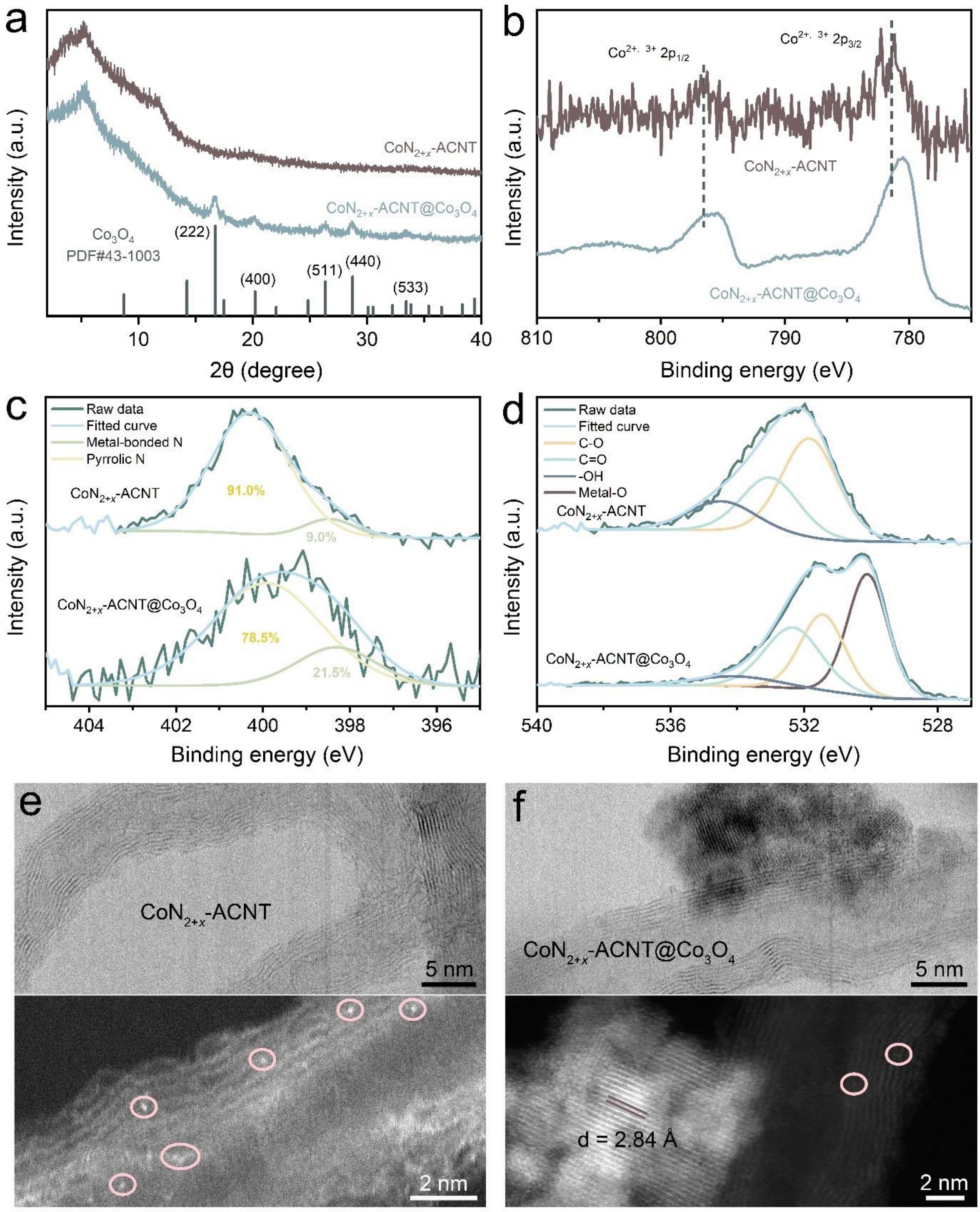

Supplementary Figure 27 a-d (**a**) X-ray powder diffraction (XRD), (**b**) XPS Co 2p, (**c**) XPS N 1s and (**d**) XPS O 1s spectra of CoN_{2+x}-ACNT with and without Co_3O_4 hybridisation. **e, f** STEM (upper panel) and HAADF-STEM (lower panel) images of (**e**) CoN_{2+x}-ACNT and (**f**) CoN_{2+x}-ACNT@Co_3O_4. The Co single sites are highlighted in pink circles.

Supplementary Figure 27 shows the chemical/physical characterizations of CoN_{2+x}-ACNT and Co_3O_4-composited CoN_{2+x}-ACNT (denoted as CoN_{2+x}-ACNT@Co_3O_4). The XRD patterns of CoN_{2+x}-ACNT and CoN_{2+x}-ACNT@Co_3O_4 are displayed in Supplementary Figure 27a, from which the existence of Co_3O_4 in the latter sample can be confirmed. The STEM images of CoN_{6+x}-ACNT@Co_3O_4 (Supplementary Figure 27f) reveal the decoration of the ACNT substrates by small Co_3O_4 nanoparticles (~20 nm). Metal single atoms can be identified in the HAADF-STEM images of both samples (the lower panels in Supplementary Figures 27e and f). XPS analysis was carried out to investigate the surface chemical information. The way more intense XPS Co 2p signal (Supplementary Figure 27b) of CoN_{2+x}-ACNT@Co_3O_4 than that of CoN_{2+x}-ACNT indicates most of the Co in CoN_{2+x}-ACNT@Co_3O_4 exist in metal oxides rather than Co-N_6 moieties. The larger proportion of metal-bonded N (Supplementary Figure 27c) in CoN_{2+x}-ACNT@Co_3O_4 (21.5 at%) than CoN_{2+x}-ACNT (9 at%) suggests part of the Co of the Co_3O_4 is bonded with N (either from the polypyrrole chains or the CoN_6 moieties). Such observation indicates that Co_3O_4 might be surrounded by N-rich electron acceptor systems. Metal-O bond can be identified in the XPS O 1s spectra of CoN_{2+x}-ACNT@Co_3O_4 (Supplementary Figure 27d).

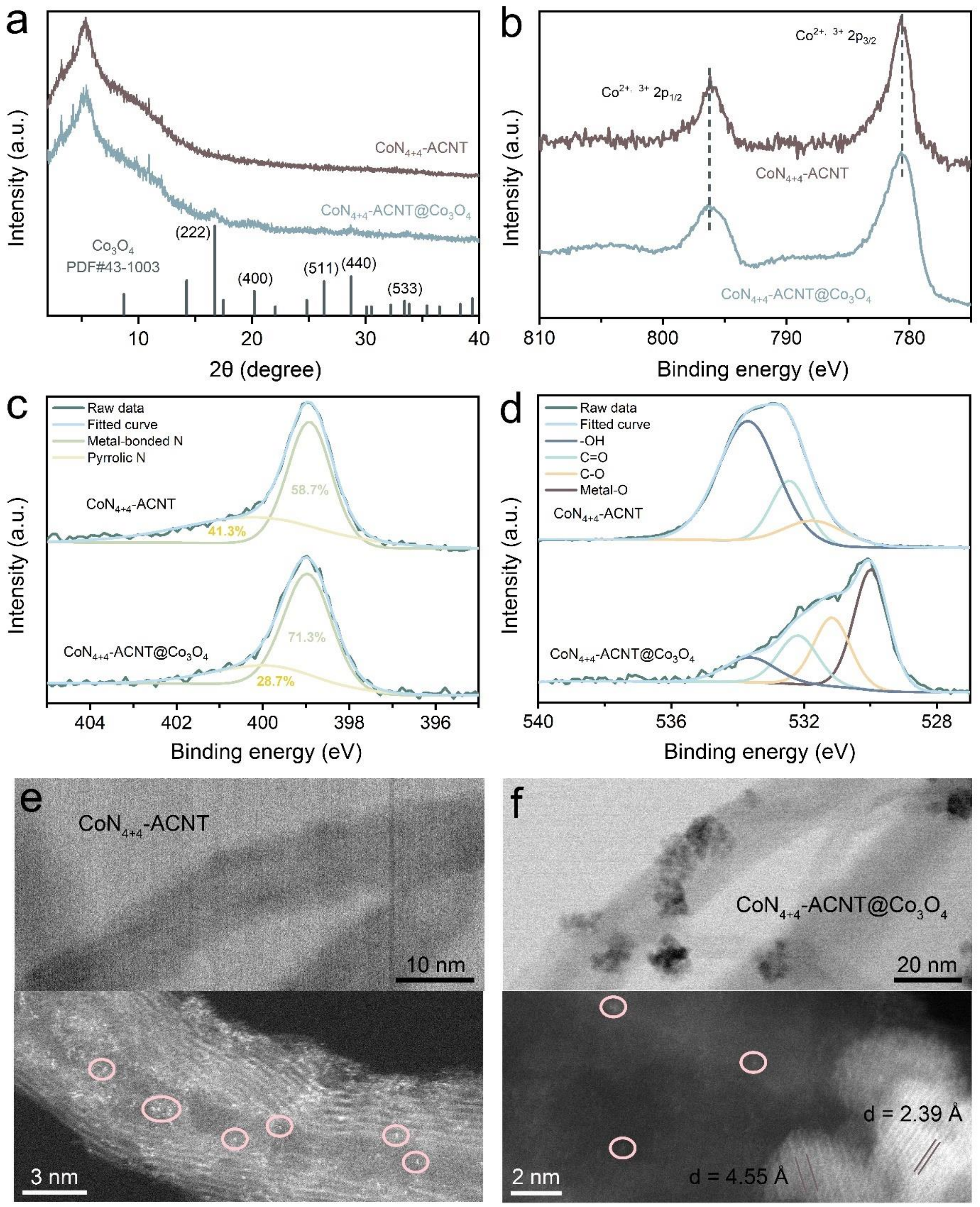

Supplementary Figure 28 a-d (**a**) XRD, (**b**) XPS Co 2p, (**c**) XPS N 1s and (**d**) XPS O 1s spectra of CoN_{4+4}-ACNT with and without Co_3O_4 hybridisation. **e, f** STEM (upper panel) and HAADF-STEM (lower panel) images of (**e**) CoN_{4+4}-ACNT and (**f**) CoN_{4+4}-ACNT@Co_3O_4. The Co single sites are highlighted in pink circles.

Supplementary Figure 28 shows the chemical/physical characterizations of CoN_{4+4}-ACNT and Co_3O_4-composited CoN_{4+4}-ACNT (denoted as CoN_{4+4}-ACNT@Co_3O_4). The XRD (Supplementary Figure 28a), STEM and HAADF-STEM (Supplementary Figures 28e and f) measurement show similar results with that in the case of CoN_{6+x}-ACNT@Co_3O_4, confirming the hybridisation of Co_3O_4 with CoN_{4+4}-ACNT. XPS analysis (Supplementary Figures 28b-d) suggests chemical bonds have been established between the Co_3O_4 and the CoN_{4+4} moieties and/or polypyrrole chains (evidencing by the increased metal-bonded N in Supplementary Figure 28c).

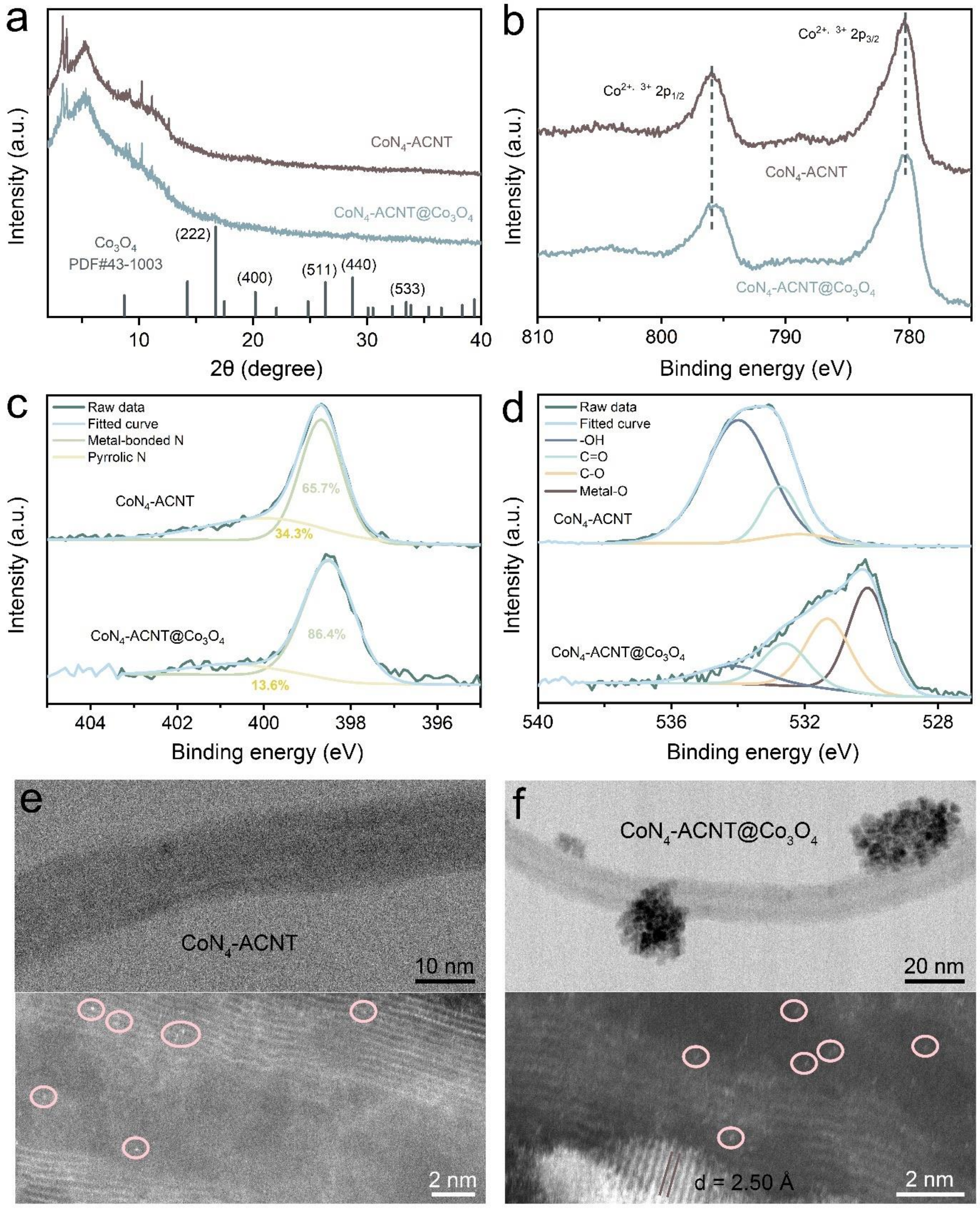

Supplementary Figure 29 a-d (**a**) XRD, (**b**) XPS Co 2p, (**c**) XPS N 1s and (**d**) XPS O 1s spectra of CoN_{4+4}-ACNT with and without Co_3O_4 hybridisation. **e, f** STEM (upper panel) and HAADF-STEM (lower panel) images of (**e**) CoN_{4+4}-ACNT and (**f**) CoN_{4+4}-ACNT@Co_3O_4. The Co single sites are highlighted in pink circles.

Supplementary Figure 29 shows the chemical/physical characterizations of CoN_{4+4}-ACNT and Co_3O_4-composited CoN_4-ACNT (denoted as CoN_4-ACNT@Co_3O_4). The XRD (Supplementary Figure 29a), STEM and HAADF-STEM (Supplementary Figures 29e and f) measurement show similar results with that in the case of CoN_{6+x}-ACNT@Co_3O_4 and CoN_{4+4}-ACNT@Co_3O_4, confirming the hybridisation of Co_3O_4 with CoN_4-ACNT. XPS analysis (Supplementary Figures 29b-d) suggests chemical bonds have been established between the Co_3O_4 and the CoN_4 moieties and/or polypyrrole chains (evidencing by the increased metal-bonded N in Supplementary Figure 29c).

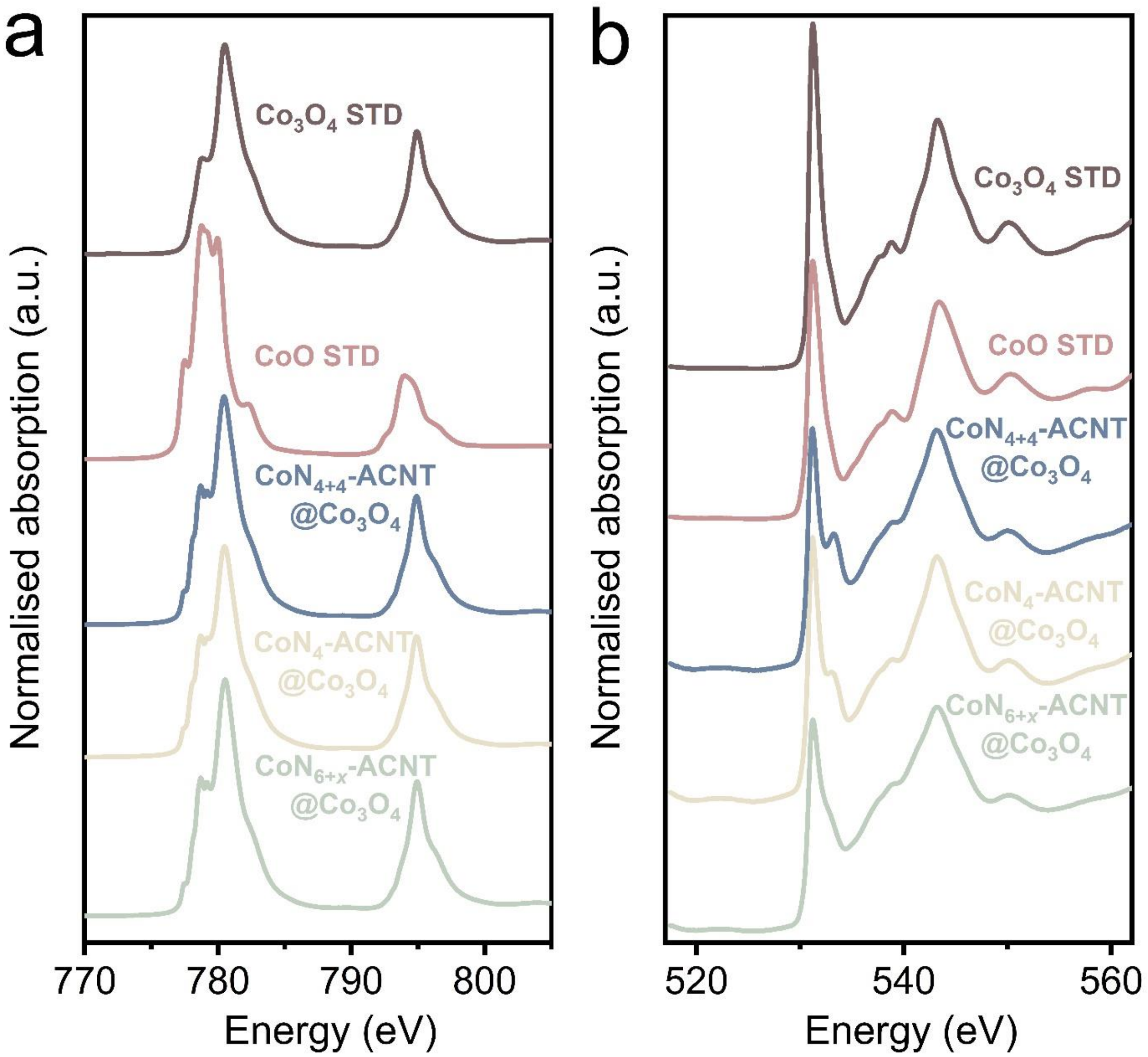

Supplementary Figure 30 a Co L_3-edge and **b** O K-edge NEXAFS spectra of CoO, Co_3O_4 and CoN_x-ACNT@Co_3O_4 series samples.

Co L_3-edge and O K-edge NEXAFS measurement was performed to explore the surface chemical information of the Co_3O_4-composited CoN_x-ACNT sample series. Two standard materials CoO and Co_3O_4 were measured as the reference. In the Co L_3-edge NEXAFS, there are four characteristic features for the high-spin Co^{2+} in octahedral coordination geometry in CoO: 777.5, 778.7, 779.1 and 780.0 eV (corresponding to the electron transition to unfilled t_{2g} and e_g orbitals).[33-36] Two characteristic features are shown for Co_3O_4: 778.7, 780.5 and 782.8 eV, corresponding to the electron transition to the half-filled e orbital of high-spin Co^{2+} in tetrahedral coordination and the e_g orbitals of low-spin Co^{3+} in octahedral coordination respectively.[33-36] Compared to the two reference samples, it is clearly to see that the catalysts with CoO_x nanoparticles is a mixture of Co^{2+} and Co^{3+} species, and the spectroscopic features indicate that both Co^{2+} and Co^{2+} are present. As for the O K-edge NEXAFS spectra, all the CoN_x-ACNT@Co_3O_4 samples show almost identical features which can be ascribed to the O from the Co_3O_4 and/or ACNT.

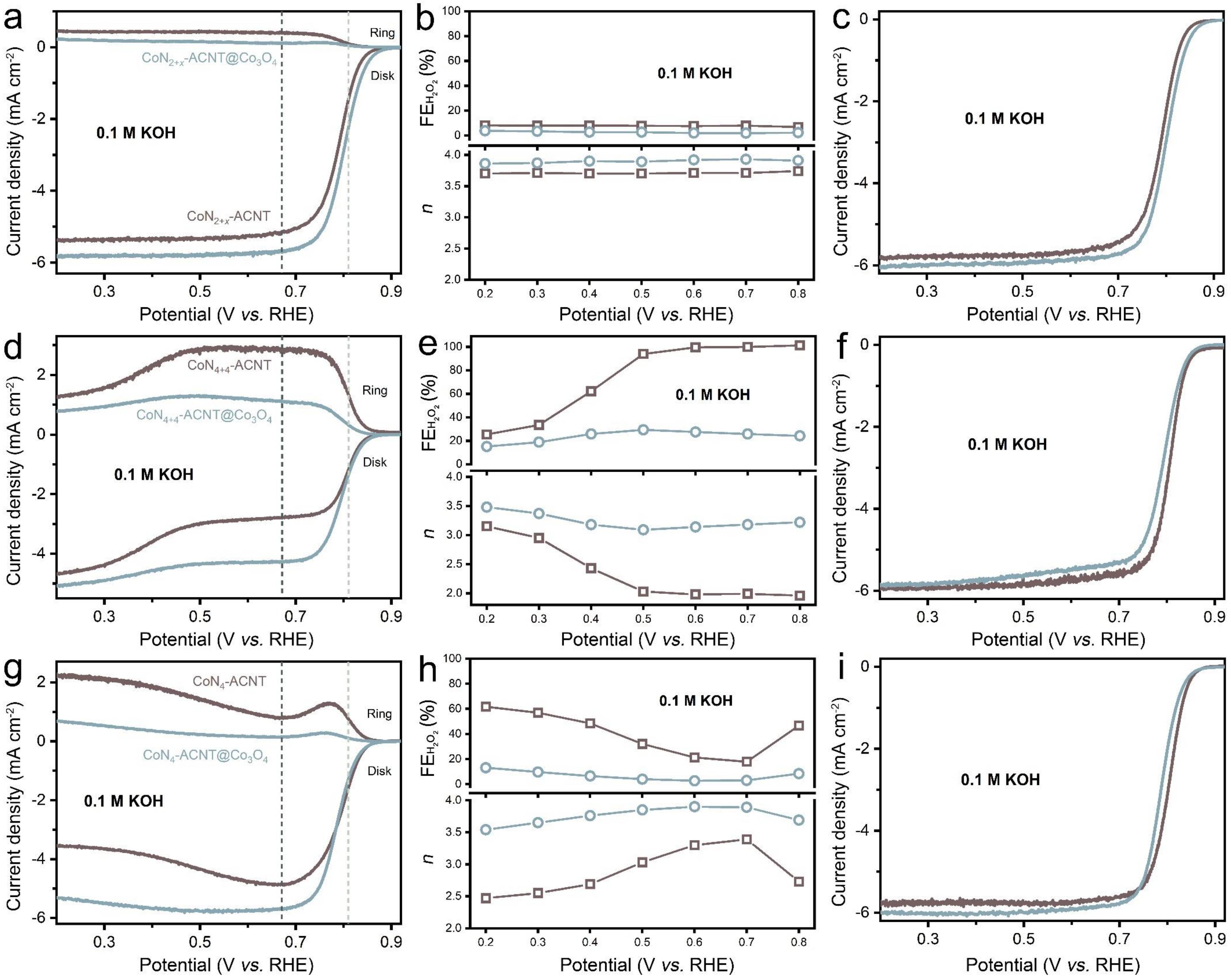

Supplementary Figure 31 a-c (**a**) ORR polarisation curves measured in O_2-satruated 0.1 M KOH at 1600 rpm (the grey and black dash lines represent the onset and half-wave potential of Co_3O_4-ACNT, the same in Supplementary Figures 31d and g), (**b**) the calculated $FE_{H_2O_2}$ and n as a function of the applied potential and (**c**) ECD analysis of CoN_{2+x}-ACNT and CoN_{2+x}-ACNT@Co_3O_4. **d-f** (**d**) ORR polarisation curves measured in O_2-satruated 0.1 M KOH at 1600 rpm, (**e**) the calculated $FE_{H_2O_2}$ and n as a function of the applied potential and (**f**) ECD analysis of CoN_{2+x}-ACNT and CoN_{2+x}-ACNT@Co_3O_4. **g-i** (**g**) ORR polarisation curves measured in O_2-satruated 0.1 M KOH at 1600 rpm, (**h**) the calculated $FE_{H_2O_2}$ and n as a function of the applied potential and (**i**) ECD analysis of CoN_{2+x}-ACNT and CoN_{2+x}-ACNT@Co_3O_4.

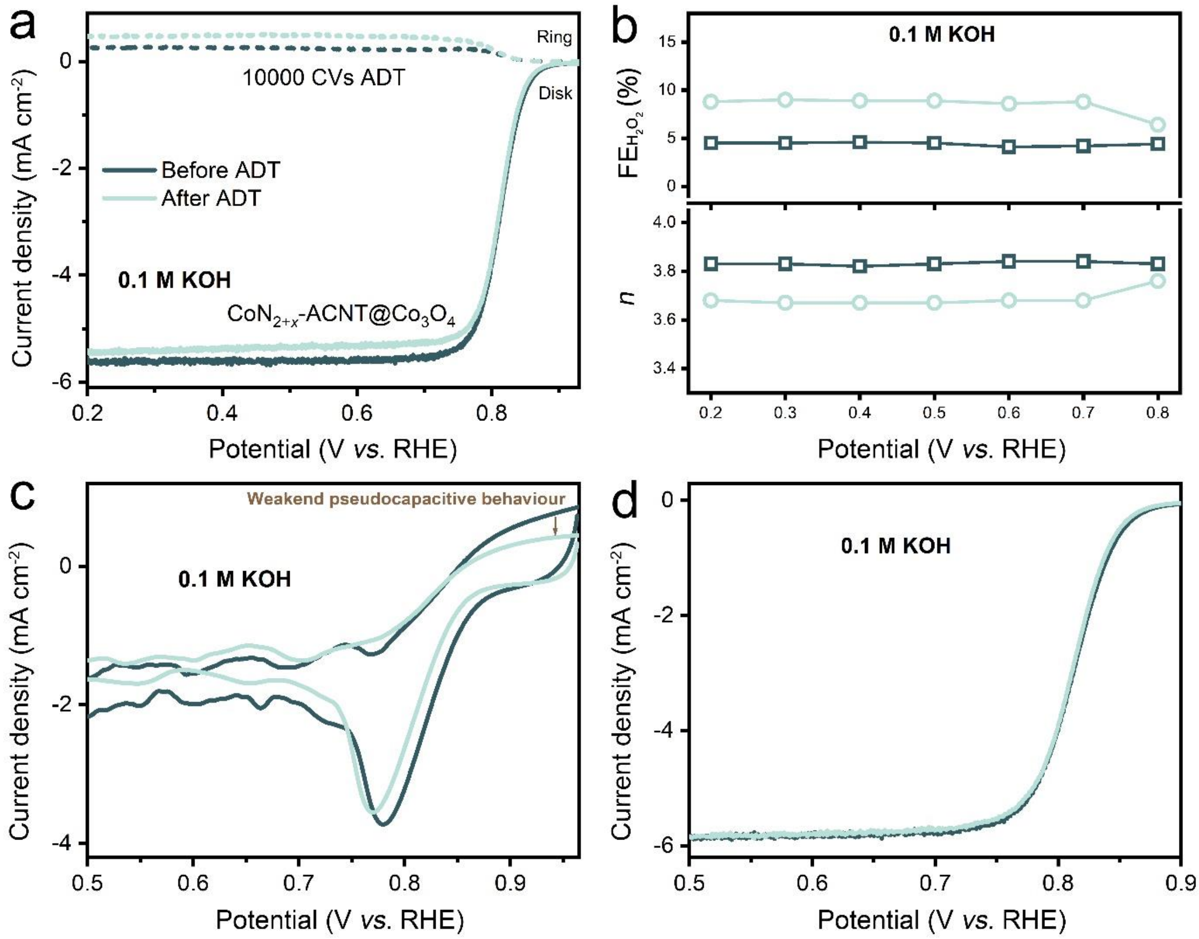

Supplementary Figure 32 ADT analysis of CoN_{2+x}-ACNT@Co_3O_4. a ORR polarisation curves before (dark green lines) and after (cyan blue lines) 10,000 cycles of CV operation (-0.8~0 V *vs.* Ag/AgCl) in O_2-saturated 0.1 M KOH. **b** The calculated $FE_{H_2O_2}$ and *n* as a function of the applied potential. **c, d** (**c**) CVs and (**d**) ECD analysis measured before and after ADT. An increase in $FE_{H_2O_2}$ can be witnessed for CoN_{2+x}-ACNT@Co_3O_4 after ADT (from ~3% to ~8%). This can be correlated with the weakened pseudocapacitive behaviour after cycling (Supplementary Figure 32c), pointing to less effective redox-induced electron delocalisation. It is noteworthy that the ECD shows no obvious change which implies the reserved O_2 reduction activity.

Supplementary Table 18 ORR performance of CoN_{4+4}-ACNT@Co_3O_4 in 0.1 M KOH.

Potential (V *vs.* RHE)	$FE_{H_2O_2}$ (%)	H_2O_2 selectivity (%)	*n*
0.8	24.18	38.94	3.22
0.7	25.72	40.91	3.18
0.6	27.45	43.08	3.14
0.5	29.29	45.31	3.09
0.4	25.79	41.01	3.18
0.3	18.82	31.68	3.37
0.2	15.1	26.24	3.48

Supplementary Table 19 ORR performance of CoN_4-ACNT@Co_3O_4 in 0.1 M KOH.

**Potential (V *vs.* RHE)**	**$FE_{H_2O_2}$ (%)**	**H_2O_2 selectivity (%)**	***n***
0.8	8.4	15.4	3.69
0.7	3	5.8	3.89
0.6	2.7	5.3	3.9
0.5	4	7.7	3.85
0.4	6.5	12.2	3.76
0.3	9.7	17.6	3.65
0.2	13.1	23.2	3.54

Supplementary Table 20 ORR performance of CoN_{2+x}-ACNT@Co_3O_4 in 0.1 M KOH.

**Potential (V *vs.* RHE)**	**$FE_{H_2O_2}$ (%)**	**H_2O_2 selectivity (%)**	***n***
0.8	2.3	4.5	3.91
0.7	1.9	3.7	3.93
0.6	2	3.9	3.92
0.5	2.7	5.3	3.89
0.4	2.7	5.2	3.9
0.3	3.4	6.6	3.87
0.2	3.8	7.2	3.86

Supplementary Table 21 ORR performance of Co_3O_4-ACNT in 0.1 M KOH.

**Potential (V *vs.* RHE)**	**$FE_{H_2O_2}$ (%)**	**H_2O_2 selectivity (%)**	***n***
0.7	2.8	5.4	3.89
0.6	3.1	6	3.88
0.5	2.8	5.5	3.89
0.4	2.6	5	3.9
0.3	2.5	4.9	3.9
0.2	2.9	5.6	3.89

Supplementary Table 22 ORR performance of Pt/C in 0.1 M KOH.

**Potential (V *vs.* RHE)**	**$FE_{H_2O_2}$ (%)**	**H_2O_2 selectivity (%)**	***n***
0.9	1.24	2.47	4
0.8	1.01	2	3.96
0.7	0.4	0.9	3.98
0.6	0.5	1	3.98
0.5	0.7	1.4	3.97
0.4	0.7	1.4	3.97
0.3	1	1.9	3.96
0.2	1.6	3.2	3.94

Reference

[1] Z. Lu, G. Chen, S. Siahrostami, Z. Chen, K. Liu, J. Xie, L. Liao, T. Wu, D. Lin, Y. Liu, T. F. Jaramillo, J. K. Nørskov, Y. Cui, *Nat. Catal.* **2018**, *1*, 156-162.
[2] J. S. Lim, J. H. Kim, J. Woo, D. S. Baek, K. Ihm, T. J. Shin, Y. J. Sa, S. H. Joo, *Chem* **2021**, *7*, 3114-3130.
[3] Y. J. Sa, J. H. Kim, S. H. Joo, *Angew. Chem. Int. Ed.* **2019**, *58*, 1100-1105.
[4] K.-H. Wu, D. Wang, X. Lu, X. Zhang, Z. Xie, Y. Liu, B.-J. Su, J.-M. Chen, D.-S. Su, W. Qi, S. Guo, *Chem* **2020**, *6*, 1443-1458.
[5] G.-F. Han, F. Li, W. Zou, M. Karamad, J.-P. Jeon, S.-W. Kim, S.-J. Kim, Y. Bu, Z. Fu, Y. Lu, S. Siahrostami, J.-B. Baek, *Nat. Commun.* **2020**, *11*, 2209.
[6] H.-X. Zhang, S.-C. Yang, Y.-L. Wang, J.-C. Xi, J.-C. Huang, J.-F. Li, P. Chen, R. Jia, *Electrochim. Acta* **2019**, *308*, 74-82.
[7] Y.-L. Wang, S.-S. Li, X.-H. Yang, G.-Y. Xu, Z.-C. Zhu, P. Chen, S.-Q. Li, *J. Mater. Chem. A* **2019**, *7*, 21329-21337.
[8] H. Gong, Z. Wei, Z. Gong, J. Liu, G. Ye, M. Yan, J. Dong, C. Allen, J. Liu, K. Huang, R. Liu, G. He, S. Zhao, H. Fei, *Adv. Funct. Mater.* **2021**, 2106886.
[9] E. Jung, H. Shin, B.-H. Lee, V. Efremov, S. Lee, H. S. Lee, J. Kim, W. Hooch Antink, S. Park, K.-S. Lee, S.-P. Cho, J. S. Yoo, Y.-E. Sung, T. Hyeon, *Nat. Mater.* **2020**, *19*, 436-442.
[10] B.-Q. Li, C.-X. Zhao, J.-N. Liu, Q. Zhang, *Adv. Mater.* **2019**, *31*, 1808173.
[11] X. Li, S. Tang, S. Dou, H. J. Fan, T. S. Choksi, X. Wang, *Adv. Mater.* **2021**, 2104891.
[12] C. Tang, Y. Jiao, B. Shi, J.-N. Liu, Z. Xie, X. Chen, Q. Zhang, S.-Z. Qiao, *Angew. Chem. Int. Ed.* **2020**, *59*, 9171-9176.
[13] K. Jiang, S. Back, A. J. Akey, C. Xia, Y. Hu, W. Liang, D. Schaak, E. Stavitski, J. K. Nørskov, S. Siahrostami, H. Wang, *Nat. Commun.* **2019**, *10*, 3997.
[14] L. Li, C. Tang, Y. Zheng, B. Xia, X. Zhou, H. Xu, S.-Z. Qiao, *Adv. Energy Mater.* **2020**, *10*, 2000789.
[15] Y. Xia, X. Zhao, C. Xia, Z.-Y. Wu, P. Zhu, J. Y. Kim, X. Bai, G. Gao, Y. Hu, J. Zhong, Y. Liu, H. Wang, *Nat. Commun.* **2021**, *12*, 4225.
[16] H. W. Kim, M. B. Ross, N. Kornienko, L. Zhang, J. Guo, P. Yang, B. D. McCloskey, *Nat. Catal.* **2018**, *1*, 282-290.
[17] G. Held, F. Venturini, D. C. Grinter, P. Ferrer, R. Arrigo, L. Deacon, W. Quevedo Garzon, K. Roy, A. Large, C. Stephens, A. Watts, P. Larkin, M. Hand, H. Wang, L. Pratt, J. J. Mudd, T. Richardson, S. Patel, M. Hillman, S. Scott, *J. Synchrotron Radiat.* **2020**, *27*, 1153-1166.
[18] A. J. Dent, G. Cibin, S. Ramos, A. D. Smith, S. M. Scott, L. Varandas, M. R. Pearson, N. A. Krumpa, C. P. Jones, P. E. Robbins, *J. Phys. Conf. Ser.* **2009**, *190*, 012039.
[19] A. J. Dent, G. Cibin, S. Ramos, S. A. Parry, D. Gianolio, A. D. Smith, S. M. Scott, L. Varandas, S. Patel, M. R. Pearson, L. Hudson, N. A. Krumpa, A. S. Marsch, P. E. Robbins, *J. Phys. Conf. Ser.* **2013**, *430*, 012023.
[20] B. Ravel, M. Newville, *J. Synchrotron Radiat.* **2005**, *12*, 537-541.
[21] S. Diaz-Moreno, S. Hayama, M. Amboage, A. Freeman, J. Sutter, G. Duller, *J. Phys. Conf. Ser.* **2009**, *190*, 012038.
[22] S. Hayama, R. Boada, J. Chaboy, A. Birt, G. Duller, L. Cahill, A. Freeman, M. Amboage, L. Keenan, S. Diaz-Moreno, *J. Phys. Condens. Matter* **2021**, *33*, 284003.
[23] S. Hayama, G. Duller, J. P. Sutter, M. Amboage, R. Boada, A. Freeman, L. Keenan, B. Nutter, L. Cahill, P. Leicester, B. Kemp, N. Rubies, S. Diaz-Moreno, *J. Synchrotron Radiat.* **2018**, *25*, 1556-1564.
[24] M. Newville, *J. Phys. Conf. Ser.* **2013**, *430*, 012007.
[25] P. E. Blöchl, *Phys. Rev. B* **1994**, *50*, 17953-17979.
[26] J. P. Perdew, K. Burke, M. Ernzerhof, *Phys. Rev. Lett.* **1996**, *77*, 3865-3868.
[27] S. Grimme, S. Ehrlich, L. Goerigk, *Journal of Computational Chemistry* **2011**, *32*, 1456-1465.
[28] V. Bambagioni, C. Bianchini, J. Filippi, A. Lavacchi, W. Oberhauser, A. Marchionni, S. Moneti, F. Vizza, R. Psaro, V. Dal Santo, A. Gallo, S. Recchia, L. Sordelli, *J. Power Sources* **2011**, *196*, 2519-2529.
[29] T. Hayashi, K. Nakamura, T. Suzuki, N. Saito, Y. Murakami, *Chem. Phys. Lett.* **2020**, *739*, 136958.
[30] P. Peng, L. Shi, F. Huo, C. Mi, X. Wu, S. Zhang, Z. Xiang, *Sci. Adv.* **2019**, *5*, eaaw2322.
[31] C. Genovese, M. E. Schuster, E. K. Gibson, D. Gianolio, V. Posligua, R. Grau-Crespo, G. Cibin, P. P. Wells, D. Garai, V. Solokha, S. Krick Calderon, J. J. Velasco-Velez, C. Ampelli, S. Perathoner, G. Held, G. Centi, R. Arrigo, *Nat. Commun.* **2018**, *9*, 935.
[32] M. Zhou, Y. Yu, K. Hu, M. V. Mirkin, *J. Am. Chem. Soc.* **2015**, *137*, 6517-6523.
[33] D. K. Bora, X. Cheng, M. Kapilashrami, P. A. Glans, Y. Luo, J. H. Guo, *J. Synchrotron Radiat.* **2015**, *22*, 1450-1458.
[34] J. Wang, J. Zhou, Y. Hu, T. Regier, *Energy Environ. Sci.* **2013**, *6*, 926-934.
[35] F. Morales, F. M. F. de Groot, P. Glatzel, E. Kleimenov, H. Bluhm, M. Hävecker, A. Knop-Gericke, B. M. Weckhuysen, *J. Phys. Chem. B* **2004**, *108*, 16201-16207.

[36] A. M. Hibberd, H. Q. Doan, E. N. Glass, F. M. F. de Groot, C. L. Hill, T. Cuk, *J. Phys. Chem. C* **2015**, *119*, 4173-4179.
